# Long-term neurodevelopmental outcomes of children perinatally infected with chikungunya: the CHIK13+ matched cohort study on Reunion Island

**DOI:** 10.1016/j.eclinm.2026.103975

**Published:** 2026-05-18

**Authors:** Raphaëlle Sarton, Marie Odile Mery, Magali Carbonnier, Michel Renouil, Camille Morice, Samir Medjane, Marc Bintner, Brahim Boumahni, Patrick Gérardin

**Affiliations:** a Department of Paediatrics, Centre Hospitalier Universitaire (CHU) de La Réunion, Saint Pierre, Reunion, France; b Centre d’Action Médico-Sociale Précoce Christian Isautier, Fondation Père Favron, Saint Louis, Reunion, France; c Department of Ophtalmology, CHU Réunion, Saint Pierre, Reunion, France; d Direction de la Recherche Clinique et de l’Innovation, CHU Réunion, Saint Pierre, Reunion, France; e Department of Neuroradiology, CHU de La Réunion, Saint Pierre, Reunion, France; f Department of Neonatology, CHU Réunion, Saint Pierre, Reunion, France; g Clinical Investigation Center, Institut National de la Santé et de la Recherche Médicale (INSERM), CHU Réunion, Saint Pierre, Reunion, France; h Plateforme de Recherche Clinique et Translationnelle, CHU Réunion, Saint Pierre, Reunion, France

**Keywords:** Arbovirus, Chikungunya, Congenital infection, Encephalitis, Neurodevelopment, Cohort study

## Abstract

**Background:**

Perinatal mother-to-child chikungunya is a rare but potentially severe infection. We assessed the long-term neurodevelopmental outcomes of perinatal mother-to-child chikungunya.

**Methods:**

In an observational matched cohort study, we evaluated children born with virologically or serologically confirmed chikungunya previously enrolled in the CHIMERE or hospital-based cohorts on Reunion Island. At the age of 13 and above, children underwent assessments of neurocognitive outcomes, adaptive functioning and behavioural skills by a neuropsychologist blinded of the exposure status, using the Wechsler Intelligence Scale for Children (WISC-V), the Vineland Adaptive Behaviour Scale (VABS-II) and parent-reported Strength and Difficulties questionnaires (SDQ), respectively. Infected children were compared with propensity score matched uninfected children born in the same epidemic context. Matching was performed a priori in birth registries on perinatal variables and month of exposure. The study was registered in ClinicalTrials (NCT04909411).

**Findings:**

Of 161 children eligible to participate in the study, 42 were enrolled between January 13, 2020, and January 14, 2021. Of them, 19 infected children (6 encephalitic; 13 non-encephalitic) were matched with 19 uninfected peers. Infected children exhibited lower mean Full-Scale Intelligence Quotient (FSIQ) than uninfected peers (82.1 ± 18.4 *versus* 91.8 ± 11.3, *p* = 0.043). Among them, 6 (32%) presented a mild to moderate cognitive deficit (FSIQ 71–85) and five (26%) a severe cognitive deficit (FSIQ ≤70) indicating intellectual disability. Infected children exhibited lower adaptive functioning and scored higher for hyperactivity/inattention.

**Interpretation:**

These results shed light on the long-term persistent disabilities of children perinatally infected with chikungunya.

**Funding:**

Centre Hospitalier Universitaire Réunion.

**Research in context**

**Evidence before this study**

Mother-to-child perinatal transmission (MTCT) of chikungunya virus was first evidenced by paediatricians during the large scale 2005–2006 outbreak in Reunion Island. We searched PubMed on March 17, 2024, for cohort studies, case series or case reports published since Jan 1, 1954, of long-term outcomes in children with a history of neonatal chikungunya. Using “chikungunya”, “chikungunya encephalitis”, “chikungunya encephalopathy”, “neurochikungunya”, “outcome”, “impairment”, “sequelae”, “neurodevelopment” and “cognitive” without added restriction, we found no study on neurodevelopmental outcomes beyond preschool age.

**Added value of this study**

To the best of our knowledge, this study is the first to prospectively evaluate the long-term neurodevelopmental outcomes of MTCT of chikungunya virus in adolescent children. This study is based on unique Reunion Island historical cohorts of children for whom exposure to infection was established ambispectively, from birth registries at the time of the outbreak, and serology at the time of evaluation. We compared the neurocognitive outcomes, adaptive functioning, and behaviour of a representative set of perinatally-infected children with those of propensity score-matched children. We found lower neurocognitive and lower adaptive functioning and more behavioural difficulties in infected children. Overall, poor neurodevelopmental outcome was observed in more than two thirds of infected children. In infected children, cognitive impairment was influenced by a history of encephalitis, small head size, or negative head growth dynamic.

**Implications of all the available evidence**

These results will inform paediatricians and childcare professionals to assist parents, teachers, and caregivers supporting children with perinatal chikungunya. In the preparedness of future chikungunya epidemics, they will serve as a basis for the development of vaccines and immunotherapies for women of childbearing age, neuroprotection of neonates, and long-term follow-up of infected neonates. These results could also motivate multifaceted interventions combining prolonged cognitive-behavioural therapy, tailored education, and special needs support to alleviate the long-term impact of chikungunya-induced disabilities.

## Introduction

*Chikungunya* virus (CHIKV) is an arbovirus of global concern spread by *Aedes* mosquitoes that can be vertically transmitted from the pregnant woman to the foetus incidentally, and to the newborn with a likelihood approaching 50 percent if the virus is present in the mother's blood during childbirth.^1^ Although CHIKV perinatal transmission is rare at community level and it only accounted for 1 in 10,000 infections during the 2005–2006 Réunion island outbreak,^2^, ^3^, ^4^ it may become a daily preoccupation for perinatal care specialists during large outbreaks, when the attack rate is high, especially around epidemic peak.^1^ Furthermore, the consequences of CHIKV-related encephalitis/encephalopathy, its most common complication, may be serious. Indeed, cerebral palsy, optic nerve atrophy, and subtle neurodevelopmental disorders have the potential to cause lifelong disabilities,^5^, ^6^, ^7^ impairing child development, and generating economic burden.^8^

CHIKV has long been regarded as a potential neuroinvasive pathogen, which is supported by abundant evidences, both experimental and clinical, neurochikungunya affecting disproportionally young infants including neonates, and elderly adults.^9^, ^10^, ^11^, ^12^, ^13^, ^14^, ^15^ CHIKV can invade and replicate in glial cells and neurons, trigger astrogliosis, apoptosis of astrocytes and neurons, as well as microglial activation, neuroinflammation,^10^^,^^12^ and to a lesser extent neuron pyroptosis, or mitochondrial dysfunction.^13^ Clinically, neurochikungunya displays a wide range of severe manifestations including encephalitis/encephalopathy, myelitis/myelopathy, Guillain-Barré syndrome, cranial nerve palsies, and neuro-ocular disease, being the most common.^11^ The immune-privileged central nervous system has even been suspected of sheltering the virus for several weeks and causing persistent viraemia and relapses of symptoms in infants.^14^^,^^15^

In the CHIMERE cohort study, we previously evidenced that half of CHIKV-perinatally-infected neonates had multiple neurocognitive dysfunctions by the age of two and that 38% of less severely affected children displayed lower Brunet-Lézine neurodevelopmental scores than uninfected peers, especially in coordination, language and social skills.^6^ This pattern was recently confirmed in a cohort from Rio de Janeiro using the Bayley Scales (BSID-III), the most widely used neurodevelopmental screening scale in the English-speaking world, and was extended to autism spectrum disorders (ASD) using the M-CHAT screening tool.^16^ Moreover, the MRI follow-up of the most severely affected children showed axon loss, gliosis and hypometabolism of N-acetyl aspartate in frontal and prefrontal white matter,^6^ cerebral areas known to regulate the abovementioned cognitive functions, as well as executive functions.

In addition, neurodevelopmental screening of 21 CHIKV-perinatally-infected children up to the age of ten revealed poor performances in verbal, non-verbal, and learning skills including planning, a complex mental function highly dependent on executive functions.^17^ We hypothesised that the neurocognitive impairments observed in children infected with the CHIKV during the perinatal period, as well as deficits in intelligence, adaptive functioning, and behaviour, would persist into adolescence, consistent with the neuroplasticity model, which considers the end of pregnancy and the first month of life to be the periods most vulnerable to brain damage and its adverse effects on neurodevelopment.^18^

Here, we report the long-term neurocognitive outcomes, adaptive functioning and behaviour of adolescent children perinatally infected with CHIKV compared with propensity score matched uninfected children, and we uncover the potential for lifelong disabilities.

## Methods

### Study design and participants

CHIK13+ is an ambispective, observational, matched cohort study in which CHIKV exposure was defined retrospectively and outcomes were assessed prospectively after the thirteenth birthday. All children born and infected through CHIKV perinatal mother-to-child transmission (MTCT) during the 2005–2006 outbreak were eligible for the study. This included the children that participated to the neurodevelopment assessment at the age of two in the CHIMERE cohort study,^3^^,^^6^ and additional children from the same background diagnosed at the time of the epidemic taken from the hospital-based historical cohorts.^1^^,^^2^

Prior to enrolment in the cohort, CHIKV-perinatally-infected children were matched in equal proportions (1:1) with uninfected peers on multiple potential confounding factors: maternal age, maternal education level, month of birth, gender, gestational age, and birthweight using Mahalanobis distance matching within propensity score callipers,^19^^,^^20^ from a pre-established list of eligible children taken from same maternity birth registers. Each infected child was assigned up to six potential uninfected peers who were offered to participate in the study. Uninfected children could have been perinatally exposed to maternal CHIKV viraemia without contracting the infection. In summary, eligible uninfected controls had to be born in the same epidemic context and to share the six matching criteria.

Children with pre-existing conditions likely to hamper the normal acquisition of developmental milestones and neurodevelopmental scores were not eligible for the study.

### Ethics approval

Both parents and children received verbal and written information on study's aims, constraints, expected risks and benefits. Children were asked to participate only if parents had given consent. Both parents and their child were asked for written consent.

The study was approved by the *Comité de Protection des Personnes Sud-Ouest et Outremer 4* (IRB: IORG0009855) and registered in both the French National Agency for Drug Safety (ANSM) database (IDRCB: 2019-A02095-52) Clinical Trials register (NCT04909411).

### Exposure

Exposure was defined at birth in perinatally-infected neonates by the presence of viral RNA using a positive RT-PCR or by the presence of CHIKV-specific IgM antibodies^1^^,^^2^ and this was also verified at the time of enrolment in the CHIK13+ cohort by the presence of CHIKV-specific IgG antibodies. A check was necessary to exclude from the CHIKV-infected group: 1- children who had been exposed before or during delivery, in whom, in the absence of maternal–foetal or postnatal infection, transplacental CHIKV-specific IgG antibodies had been eliminated within 32 months^21^; 2- children who had not been exposed before or during delivery, but who could have been infected during early childhood over the epidemic, as it is also known that early infection during early childhood has adverse effects on neurological development.^11^^,^^22^

### Outcomes

The primary endpoint was the full-scale intelligence quotient (FSIQ) of the Wechsler Intelligence Scale for Children® fifth edition (WISC-V) which provides an overall rating of cognitive performance.^23^ This scale assesses verbal comprehension, visual spatial skills, fluid reasoning, working memory and processing speed. For the FSIQ and each of its indices, cognitive deficit was deemed severe for scores of 55 or less (≤−3 standard deviations), moderate for scores of 56–70 (−3 to ≤ −2 SD), mild for scores 71–85 (−2 to ≤ −1 SD) or absent for scores above 85. A FSIQ of 70 or less (≤−2 SD) indicated intellectual disability (DSM-V).

Secondary endpoints included the adaptive behaviour composite (ABC) score of the Vineland Adaptive Behaviour Scale second edition (VABS-II),^24^ and the total difficulties score of the Strengths and Difficulties Questionnaire (SDQ).^25^

We also assessed a composite binary indicator of all identified impairments in the three abovementioned scales, as done in the French MONALISA cohort.^26^ With this, poor neurodevelopmental outcome was defined as a FSIQ and/or ABC score ≤85 and/or SDQ total difficulties score higher than 13. Severe neurodevelopmental impairment was defined as at least one of the abovementioned scores less than 70 or higher than 16.

Neurocognitive and behavioural assessments were performed by a single skilled neuropsychologist (MOM) blinded to the infectious status.

### Covariates

Neonatal characteristics were retrieved from maternity birth registers and linked with previous cohort data.^1^, ^2^, ^3^^,^^6^^,^^17^ This helped to distinguish the different phenotypes among infected children (encephalitic *vs* non-encephalitic; small-head *vs* non-normal-sized head), or according to head growth dynamic (positive *vs* negative; *i.e.*, centile gain *vs* centile loss).^27^

The data collected included parental professions and highest socio-professional category in the couple, a deprivation index, number of siblings, sibling school grades, maternal education level, pregnancy (parity, gestational diabetes, pregnancy-induced hypertension disorders) and perinatal variables (gestational age, birth height, birthweight, head circumference, low birthweight and small for gestational age, Apgar scores), the medical history of the child, breastfeeding, and growth parameters (height, weight, head circumference, growth dynamics, stunting, wasting, overweight and obesity).

### Statistical analysis

Sample size was based on expected recruitment from the CHIMERE cohort. We anticipated enrolling about two thirds of the 33 CHIKV-perinatally-infected children, each matched 1:1 with an uninfected control using psmatch2 in Stata. A difference of 1 SD (15 FSIQ points) in 21 pairs would provide 88% statistical power (α = 0.05). Primary analyses used propensity score matching from a causal inference perspective. Secondary sensitivity analyses were conducted after unmatching. In primary analyses, means were compared with Wilcoxon signed-rank tests, proportions with McNemar tests. For binary outcomes that were significant, we measured effect sizes using conditional Poisson regression models. Subscale scores were correlated together using Spearman rho (ρ) coefficients.

Finally, we conducted a longitudinal analysis among the infected children on similar IQ scales to test whether the cognitive development continued to change over time between the previous assessment at two years of age in the CHIMERE cohort and the new assessment at adolescence age in the CHIK13+ cohort. To this end, we considered that a long-term outcome could be predicted from an early measure if it met three criteria: a significant correlation between the two score distributions, the absence of a significant score difference in individual pairwise comparisons, and a higher than expected agreement between score classifications (*i.e.*, absence of individual significant change and progression within same standard deviations corridor of performances throughout development). In this, correlations between the two measure distributions were tested using Spearman rho (ρ) coefficients, the difference between two similar pairwise measures using Wilcoxon signed-rank tests, and inter-agreement between the two score categories using Cohen's kappa (κ) tests.

We used Stata (v16.1, StataCorp, College Station, Tx, USA, 2019) for analysis. Observations with missing data were eliminated and a two-tailed *p* less than 0.05 was considered significant.

Full methodological details are provided in the appendix (File S1).

### Role of the funding source

The funder had no role in had no role in study design, data collection, data analysis, data interpretation, or writing of the report.

## Results

**Fig. 1:**
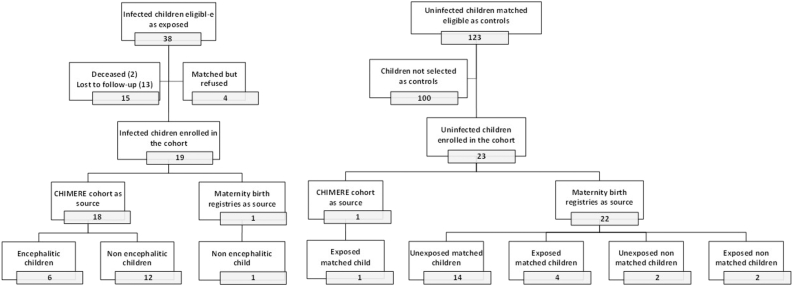
**Study population.** Flow charts of study participants.

Of the 38 CHIKV-perinatally-infected children diagnosed during the chikungunya epidemic between April 28, 2005, and May 17, 2006, all were liveborn. Of the 36 survivors (1 death in the neonatal period, 1 after), 13 were lost-to-follow-up, four were matched but the parents refused their child to participate, which left 19 infected children eligible for neurodevelopmental assessment (Fig. 1). These 19 infected children were matched with 19 uninfected children and constituted the main population, while four uninfected children were left off main analyses and reserved to the sensitivity and subsidiary analyses.

The 42 children (median age 14.4 years, extremes 13.7–15.5), enrolled between January 13, 2020, and January 14, 2021.

CHIKV-perinatally-infected children in the study did not differ in terms of age, gender, maternal and neonatal characteristics, nor in terms of exposure or clinical presentation, from their infected peers lost-to-follow-up or those who declined to participate, which ensured a likely representative case-mix relative to the targeted population (Table S1). The uninfected control group was representative of source population (Table S2).

Baseline data are presented in Table 1 for the main population and Table S3 for the sensitivity analysis population. CHIKV-perinatally-infected children were slightly younger than uninfected children (−3.6 months). However, they did not differ on other matching criteria (maternal age, maternal education level, gender, gestational age, birthweight) nor on other maternal and neonatal characteristics, or breastfeeding.

**Table 1:** Characteristics of 38 matched infected-uninfected children exposed to perinatal mother-to-child transmission of chikungunya, CHIK13+ cohort, Reunion island, 2020–2021.

Exposure group	Total		CHIK−		CHIK+		SMD
	n = 38	(%)	n = 19	(%)	n = 19	(%)	
Environmental characteristics							
Neighbourhood deprivation							
Absent	9	(23.7)	4	(21.1)	5	(26.3)	0.000
Moderate	12	(31.6)	7	(36.8)	5	(26.3)	
High	17	(44.7)	8	(42.1)	9	(47.4)	
Higher socio-professional category of parents							
Executive and upper professions	3	(7.9)	2	(10.5)	1	(5.3)	0.141
Intermediate	7	(18.4)	5	(26.3)	2	(10.5)	
Farmers, craftsmen, entrepreneurs	8	(21.0)	2	(10.5)	6	(31.6)	
Employees	0	(0.0)	0	(0.0)	0	(0.0)	
Workers	5	(13.2)	3	(15.8)	2	(10.5)	
Pensioners	12	(31.6)	6	(31.6)	6	(31.6)	
Unemployed	3	(7.9)	1	(5.3)	2	(10.5)	
Other siblings at home							
0	3	(7.9)	2	(10.5)	1	(5.3)	0.105
1–2	15	(39.5)	7	(36.8)	8	(42.1)	
≥3	20	(52.6)	10	(52.6)	10	(52.6)	
Maternal characteristics							
Age^a^ (years; median, Q_1_–Q_3_)	29.1	(24.7–34.7)	30.2	(24.7–34.2)	28.2	(24.3–36.9)	0.085
Education^a^							
Lower than college	7	(18.4)	3	(15.8)	4	(21.1)	0.000
Lower than baccalaureate	16	(42.1)	9	(47.4)	7	(36.8)	
Baccalaureate or higher	15	(39.5)	7	(36.8)	8	(42.1)	
Parity							
0	13	(34.2)	7	(36.8)	6	(31.6)	−0.043
1–3	23	(60.5)	12	(63.2)	11	(57.9)	
≥4	2	(5.3)	0	(0.0)	2	(10.5)	
Prepregnancy body mass index							
(kg/m^2^; median, Q_1_–Q_3_)	24.4	(21.7–31.2)	26.5	(20.8–32.9)	24.0	(21.7–29.9)	−0.135
<25	19	(51.4)	9	(47.4)	10	(55.6)	0.216
25–29.9	8	(21.6)	4	(21.0)	4	(22.2)	
≥30	10	(27.0)	6	(31.6)	4	(22.2)	
Smoking during pregnancy	4	(10.5)	3	(15.8)	1	(5.3)	0.348
Child characteristics at birth							
Male gender^a^	25	(65.8)	12	(63.2)	13	(68.4)	0.111
Gestational age^a^ (weeks; median, Q_1_–Q_3_)	38	(38–39)	38	(38–39)	38	(37–39)	0.328
Preterm birth	5	(13.2)	2	(10.5)	3	(15.8)	0.156
Birthweight^a^ (kg; median, Q_1_–Q_3_)	3.13	(2.89–3.35)	3.18	(3.03–3.41)	3.10	(2.62–3.34)	0.448
Low birthweight	4	(10.5)	1	(5.3)	3	(15.8)	**−0.348**
Height at birth (cm; median, Q_1_–Q_3_)	49	(48–50)	50	(49–51)	48	(46–50)	**0.975**
Small for gestational age							
10th-3rd centile	4	(10.5)	2	(10.5)	2	(10.5)	**−0.413**
<3rd centile	2	(5.3)	0	(0.0)	2	(10.5)	
Head circumference (cm; median, Q_1_–Q_3_)	34	(33–35)	34	(33–35)	34	(32–35)	0.253
Head circumference z-scores (95% CI)	−0.208	(−0.457–0.042)	−0.143	(−0.454–0.168)	−0.272	(−0.691–0.147)	−0.057
Head circumference categories							
Normal head, −1 SD ≤ to ≤ +2 SD	32	(84.2)	18	(94.7)	14	(73.7)	**−0.394**
Small head, −2 SD ≤ to < −1 SD	6	(15.8)	1	(5.3)	5	(26.3)	
Microcephaly, <−2 SD	0	(0.0)	0	(0.0)	0	(0.0)	
Apgar score at 1 min							
10	29	(76.3)	14	(73.7)	15	(78.9)	**0.231**
7–9	4	(10.5)	3	(15.8)	1	(5.3)	
<7	5	(13.2)	2	(10.5)	3	(15.8)	
Breastfeeding	22	(57.9)	13	(68.4)	9	(47.4)	0.436
Characteristics of exposure							
Month of birth in the epidemic (median, Q_1_–Q_3_)^a^	11	(11–12)	12	(11–13)	11	(9–12)	0.519
Epidemic waves							
1 (March to August 2005)	4	(10.5)	1	(5.3)	3	(15.8)	**0.348**
2 (September 2005 to August 2006)	34	(89.5)	18	(94.7)	16	(84.2)	
Child characteristics at follow-up							
Age (years; medians, Q_1_–Q_3_)	14.4	(14.1–14.6)	14.2	(13.0–14.4)	14.5	(14.4–14.8)	**−0.978**
13–14	7	(18.4)	6	(31.6)	1	(5.3)	**−0.877**
14–15	31	(81.5)	13	(68.4)	18	(94.7)	
Height (cm; medians, Q_1_–Q_3_)	165	(159–175)	170	(159–176)	162	(157–172)	0.415
Stunted (inappropriate height/age)	5	(13.2)	1	(5.3)	4	(21.0)	−0.498
Weight (kg; median, Q_1_–Q_3_)	59	(48–69)	56.5	(49–69)	60	(48–71)	0.091
Wasted (inappropriate weight/height)	7	(18.4)	2	(10.5)	5	(26.3)	**−0.488**
Obese	9	(23.7)	2	(10.5)	7	(36.8)	−0.147
Head circumference (cm; median, Q_1_–Q_3_)	54	(53–56)	56	(54–57.5)	53	(52–54.4)	**1.291**
Head circumference z-scores (95% CI)	−0.041	(−0.383–0.302)	0.517	(−0.097–0.936)	−0.599	(−1.035 to −0.162)	1.291
Head circumference categories							
Large head > + 2 SD	6	(15.8)	5	(26.3)	1	(5.3)	**−1.097**
Normal head, −1 SD ≤ to ≤ +2 SD	18	(47.4)	13	(68.4)	5	(26.3)	
Small head, −2 SD ≤ to < −1 SD	12	(31.6)	1	(5.3)	11	(57.9)	
Microcephaly, <−2 SD	2	(5.2)	0	(0.0)	2	(10.5)	

Data are numbers and column percentages, or medians and interquartile ranges (Q1–Q3) when specified.

Percentages and medians compared using the standardised mean difference (SMD), *i.e.*, Cohen's δ effect size index, as appropriate. SMDs in bold indicate variables that are unbalanced between groups.

a Maternal age, maternal education, month of birth, gender, gestational age and birthweight were used for propensity score matching.

On follow-up, CHIKV-perinatally-infected and their uninfected peers shared similar physical features, nutritional and growth parameters, with exception of head circumference.

CHIKV-perinatally-infected children presented a smaller median head circumference (−3 cm) and two cases of severe microcephaly (<−3 SD) of which one was of post-natal onset, the other congenital (primary) with a sharp reduction in postnatal head growth (Table 1, Table S3). The investigation of head growth has been detailed elsewhere.^27^ CHIKV-perinatally-infected children were also more likely to experience sensorineural disorders during childhood resulting from *sequelae* of neonatal encephalitis, than their uninfected peers, visual acuity deficit (58% *vs* 22%, *p* = 0.016) and strabismus (37% *vs* 4%, *p* = 0.015) being the most noticeable.

Neurodevelopmental outcomes are compared in Tables 2 and 3, Table S4 and S5. Subsidiary outcomes are given as supporting information in Table S6.

**Fig. 2:**
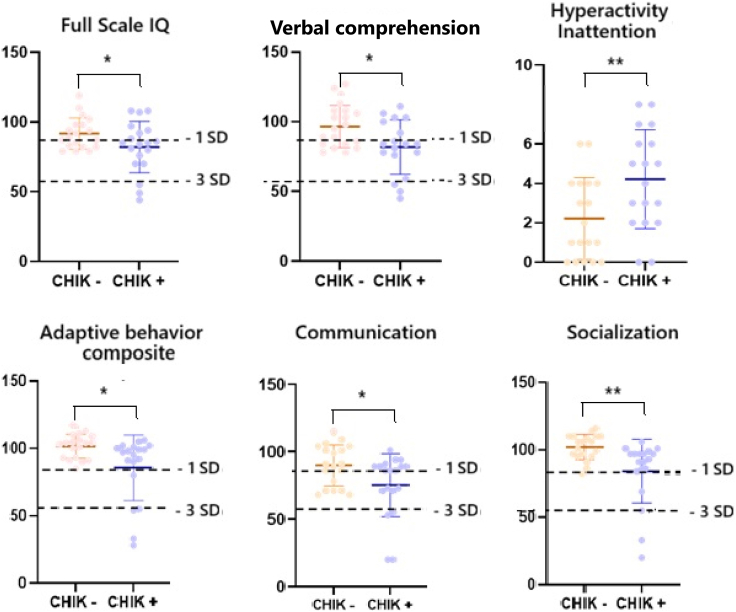
**Significantly impaired domains in cognitive, adaptive and behavioural skills of 19 children infected with perinatal chikungunya, CHIK13+ cohort, Reunion island, 2020–2021.** SD: standard deviation. ∗*p* < 0.05; ∗∗*p* < 0.01.

CHIKV-perinatally-infected children exhibited lower mean FSIQ than their uninfected peers (82.1 ± 18.4 *vs* 91.8 ± 11.3, *p*= 0.043; Table 2 and Fig. 2) in primary analysis and a similar non-significant trend in secondary sensitivity analysis (Table S4). Overall, six of the 19 (32%) infected children presented mild to moderate cognitive deficit (FSIQ 71–85) and five (26%) severe cognitive impairment (FSIQ ≤70) indicating mental retardation. This represented a 1.57 relative risk (RR) of cognitive impairment (95% CI 0.89–2.76) and a higher risk of intellectual disability (5/19 *versus* 0/19, *p* < 0.001), respectively. Although we observed similar trends in lower scores for all cognitive dimensions among CHIKV-perinatally-infected children, the mean differences were only significant for verbal comprehension and, to a lesser extent, for processing speed (Table 2, Table S4). These two indices were positively correlated together (rho = 0.53, *p* < 0.001) and with the three other WISC-V indices in this group (File S2, Table S7, Panel A). This highlights the potential for massive cognitive impairment (*i.e*., consistent impairment in the 5 cognitive dimensions), as observed in five children, following CHIKV perinatal MTCT: three encephalitic children with massive cognitive impairment affecting working memory; one encephalitic child with mild cognitive impairment and very low adaptive behaviour; and one non-encephalitic child with a normal IQ exhibiting a working memory deficit and experiencing major difficulties in social behaviours (conduct problems, peer relationships, hyperactivity/inattention).

**Table 2:** Neurodevelopmental average scores of 38 matched infected-uninfected children exposed to perinatal mother-to-child transmission of chikungunya, CHIK13+ cohort, Reunion island, 2020–2021.

Exposure group	Total		CHIK -		CHIK +		*p value*
	n = 38		n = 19		n = 19		
Subtest averages	Mean	(95% CI)	Mean	(95% CI)	Mean	(95% CI)	
WISC-V (Wechsler Intelligence Scale-5th ed) scores							
Full Scale Intelligence Quotient	86.9	(81.7–92.1)	91.8	(86.3–97.2)	82.1	(73.1–90.9)	**0.043**
Verbal comprehension	89.5	(72.2–91.6)	97.3	(89.7–104.8)	81.9	(72.2–91.6)	**0.015**
Visual spatial	91.9	(86.9–96.9)	95.7	(89.8–101.5)	88.1	(79.9–96.3)	0.151
Fluid reasoning	91.1	(86.0–96.1)	94.8	(89.5–100.0)	87.4	(78.4–96.3)	0.125
Working memory	86.2	(81.5–90.9)	87.7	(82.3–93.0)	84.7	(76.6–92.9)	0.482
Processing speed	92.4	(86.8–97.9)	99.1	(93.9–104.2)	85.7	(76.4–95.0)	0.058
VABS-II (Vineland Adaptive Behaviour Scale-2d ed) scores							
Adaptive behaviour composite	93.6	(87.1–100.1)	101.6	(97.3–105.8)	85.6	(73.9–97.4)	**0.018**
Communication	82.6	(75.7–89.4)	89.9	(82.5–97.2)	75.3	(64.0–86.5)	**0.023**
Daily living skills	105.2	(97.7–112.6)	113.0	(109.5–116.4)	97.3	(83.0–111.6)	0.164
Socialisation	93.1	(86.5–99.7)	102.1	(97.4–106.6)	84.2	(72.8–95.6)	**0.003**
Parent-reported SDQ (Strength and Difficulties Questionnaire) scores							
Total difficulties score	10.1	(8.2–11.8)	9.3	(6.2–12.4)	10.8	(8.7–12.9)	0.402
Emotional symptoms	2.7	(1.2–3.3)	3.1	(1.9–4.1)	2.3	(1.2–3.3)	0.381
Conduct problems	1.7	(1.0–2.4)	1.7	(0.9–2.4)	1.7	(1.0–2.4)	0.389
Hyperactivity/inattention	3.2	(2.3–4.1)	2.2	(1.1–3.3)	4.2	(2.9–5.5)	**0.009**
Peer relationships problems	2.4	(1.9–3.0)	2.3	(1.2–3.3)	2.6	(2.0–3.1)	0.082
Prosocial behaviour	8.1	(7.3–8.8)	8.4	(7.2–9.6)	7.7	(6.7–8.7)	0.244

Maternal age, maternal education, month of birth, gender, gestational age and birthweight were used for propensity score matching.

Data are means and 95% confidence intervals. Paired means are compared using Wilcoxon signed-rank tests. Bold characters indicate significant *p*-values.

Infected children exhibited lower mean VABS-II ABC scores than their uninfected peers in both primary (85.6 ± 24.3 *vs* 101.6 ± 8.9, *p* = 0.010; Table 2 and Fig. 2) and secondary analyses (Table S4). Overall, one of the 19 infected children presented low adaptive behaviour (ABC score 71–85) and four presented very low adaptive behaviour (ABC score ≤70) indicating poor autonomy and severe maladaptation (Table 3). This represented a non-significant five-fold higher risk of maladaptive behaviour (RR 5.0 95% CI 0.55–45.39) and a highly significant risk of severe maladaptation (4/19 *versus* 0/19, *p* < 0.001). Interestingly, the ABC score positively correlated with other VABS-II scores in communication, daily living skills and socialisation (rho = 0.80, 0.78, 0.85, each *p* < 0.001, respectively) as well as with FSIQ, WISC-V verbal comprehension, visual spatial and fluid reasoning indices (rho = 0.54, 0.51, 0.67, 0.61, each *p* < 0.016, respectively), which elucidates the intricate relation between cognition and adaptive behaviour in infected children (File S2, Table S7, Panel A).

**Table 3:** Cognitive, adaptative and behavioural skills of 38 matched infected-uninfected children exposed to mother-to-child perinatal transmission of chikungunya, CHIK13+ cohort, Reunion island, 2020–2021.

Exposure group	Total		CHIK−		CHIK+		*p value*
	n = 38		n = 19		n = 19		
	n	(%)	n	(%)	n	(%)	
Neurocognitive deficits (WISC-V)							
Full scale intelligence quotient							b + c vs a:
Age-appropriate skill (a)	20	(52.6)	12	(63.2)	8	(42.1)	0.796
Mild to moderate deficit (b)	13	(34.2)	7	(36.8)	6	(31.6)	c vs a+b:
Severe deficit/intellectual disability (c)	5	(13.2)	0	(0.0)	5	(26.3)	<**0.001**
Verbal comprehension							b + c vs a:
Age-appropriate skill (a)	20	(54.1)	13	(68.4)	7	(38.9)	0.781
Mild to moderate deficit (b)	13	(35.1)	6	(31.6)	7	(38.9)	c vs a+b:
Severe deficit (c)	4	(10.8)	0	(0.0)	4	(22.2)	<**0.001**
Visual spatial							b + c vs a:
Age-appropriate skill (a)	27	(71.1)	15	(79.0)	12	(63.2)	0.077
Mild to moderate deficit (b)	8	(21.0)	4	(21.0)	4	(21.0)	c vs a+b:
Severe deficit (c)	3	(7.9)	0	(0.0)	3	(15.8)	**<0.001**
Fluid reasoning							b + c vs a:
Age-appropriate skill (a)	26	(68.4)	15	(78.9)	11	(57.9)	0.118
Mild to moderate deficit (b)	8	(21.1)	4	(21.1)	4	(21.0)	c vs a+b:
Severe deficit (c)	4	(10.5)	0	(0.0)	4	(21.0)	**<0.001**
Working memory							b + c vs a:
Age-appropriate skill (a)	20	(52.6)	10	(52.6)	10	(52.6)	0.818
Mild to moderate deficit (b)	14	(36.8)	9	(47.4)	5	(26.3)	c vs a+b:
Severe deficit (c)	4	(10.5)	0	(0.0)	4	(21.0)	**<0.001**
Processing speed							b + c vs a:
Age-appropriate skill (a)	28	(73.7)	18	(94.7)	10	(52.6)	**0.012**
Mild to moderate deficit (b)	7	(18.4)	1	(5.3)	6	(31.6)	c vs a+b:
Severe deficit (c)	3	(7.9)	0	(0.0)	3	(15.8)	**<0.001**
Delayed adaptive skills (VABS-II)							
Adaptive behaviour composite							b + c vs a:
Normal adaptive (a)	32	(84.2)	18	(94.7)	14	(73.7)	**0.001**
Low adaptive (b)	2	(5.3)	1	(5.3)	1	(5.3)	c vs a+b:
Very low adaptive (c)	4	(10.5)	0	(0.0)	4	(21.0)	**<0.001**
Communication							b + c vs a:
Normal adaptive (a)	24	(63.2)	13	(68.4)	11	(57.9)	0.225
Low adaptive (b)	8	(21.0)	4	(21.0)	4	(21.0)	c vs a+b:
Very low adaptive (c)	6	(15.8)	2	(10.5)	4	(21.0)	**0.002**
Daily living skills							b + c vs a:
Normal adaptive (a)	33	(86.8)	19	(100.0)	14	(73.7)	**<0.001**
Low adaptive (b)	1	(2.6)	0	(0.0)	1	(5.3)	c vs a+b:
Very low adaptive (c)	4	(10.5)	0	(0.0)	4	(21.0)	**<0.001**
Socialisation							b + c vs a:
Normal adaptive (a)	32	(84.2)	18	(94.7)	14	(73.7)	**0.001**
Low adaptive (b)	8	(21.1)	1	(5.3)	1	(5.3)	c vs a+b:
Very low adaptive (c)	4	(10.5)	0	(0.0)	4	(21.0)	**<0.001**
Parent-reported SDQ scores							
Total difficulties score							b + c vs a:
Absent (a)	27	(75.0)	14	(77.8)	13	(72.2)	**0.049**
Mild to moderate (b)	3	(8.3)	1	(5.5)	2	(11.1)	c vs a+b:
High (c)	6	(16.7)	3	(16.7)	3	(16.7)	**0.007**
Emotional symptoms							b + c vs a:
Absent (a)	21	(63.6)	11	(61.1)	10	(66.7)	0.467
Mild to moderate (b)	4	(12.2)	1	(5.6)	3	(20.0)	c vs a+b:
High (c)	8	(24.2)	6	(33.3)	2	(13.3)	0.167
Conduct problems							b + c vs a:
Absent (a)	27	(75.0)	15	(83.3)	12	(66.7)	**0.035**
Mild to moderate (b)	6	(16.7)	2	(11.1)	4	(22.2)	c vs a+b:
High (c)	3	(8.3)	1	(5.6)	2	(11.1)	**<0.001**
Hyperactivity/inattention							b + c vs a:
Absent (a)	28	(77.8)	16	(88.9)	12	(73.7)	**0.013**
Mild to moderate (b)	4	(11.1)	2	(11.1)	2	(11.1)	c vs a+b:
High (c)	4	(11.1)	0	(0.0)	4	(22.2)	**<0.001**
Peer relationships problems							b + c vs a:
Absent (a)	17	(47.2)	10	(55.5)	7	(38.9)	0.796
Mild to moderate (b)	13	(36.1)	5	(27.8)	8	(44.4)	c vs a+b:
High (c)	6	(16.7)	3	(16.7)	3	(16.7)	**0.007**
Prosocial behaviour							b + c vs a:
Normal (a)	26	(72.2)	14	(77.8)	12	(66.6)	0.077
Intermediate (b)	7	(19.4)	2	(11.1)	5	(27.8)	c vs a+b:
Abnormal (c)	3	(8.3)	2	(11.1)	1	(5.6)	**<0.001**

WISC-V: Wechsler Intelligence Scale-5th ed scale. VABS-II: Vineland Adaptive Behaviour Scale-2d ed scale. SDQ: Strength and Difficulties Questionnaire. Maternal age, maternal education, month of birth, gender, gestational age and birthweight were used for propensity score matching. Cognitive functions, adaptive skills and behavioural skills are categorised as follows: (a) Normal: score > −1 SD; (b) Low, mildly or moderately impaired: < −2 SD score ≤ −1 SD; (c) Very low or severely -impaired: score ≤ −2 SD. Discordant pairs are compared using Mc Nemar tests. Bold characters indicate significant *p*-values.

CHIKV-perinatally-infected children did not differ from uninfected children in mean SDQ total difficulties in both primary (10.8 ± 4.2 *vs* 9.3 ± 6.1, *p* = 0.402; Table 2 and Fig. 2) and secondary analyses. By contrast, total difficulties, conduct problems, and hyperactivity/inattention were more likely in infected children (Table 3). To a lesser extent, encephalitic children were also more likely to encounter problems in peer relationships and less likely to exhibit normal prosocial behaviour. Of note, when these associations were turned into effect measures, only hyperactivity/inattention yielded a highly significant risk for the infected group (RR 1083, 95% CI 391–2996). Indeed, among the group of CHIKV-perinatally-infected children, hyperactivity/inattention score was an important driver of total difficulties (rho = 0.62, *p* = 0.006) whose score negatively correlated with FSIQ score, WISC-V visual spatial, fluid reasoning and working memory indices (rho = −0.66, −0.71, −0.65, −0.72, each *p* < 0.004) but it did not correlate with any of the VABS-II scores (File S2, Table S7, Panel A). Of note, three of the five infected children diagnosed with hyperactivity/inattention had no history of neonatal encephalitis, but all had negative head growth dynamics and three were already small headed.

Overall, 13 CHIKV-perinatally-infected children (68%) had a poor neurodevelopmental outcome; seven (37%) severe (OR 6.1, 95% CI 1.06–35.97).

Interestingly, we observed fewer (56% *versus* 68%, *p* = 0.042) subscale 2 × 2 correlations in infected children (File S2, Table S7, Panel A) than in their uninfected peers (File S2, Table S7, Panel B).

In longitudinal analyses, we found that overall cognitive and adaptive performances of adolescent children could not be predicted from toddler's Brunet-Lézine score performances (File S2, Table S8, Panel A). Subgroup analyses revealed that in nonencephalitic children, head growth dynamics showed no significant differences between infancy and childhood across the three different comparisons, which means head size at adolescence could be predictable from early head growth dynamics (File S2, Table S8, Panel B; Figure S1). In encephalitic children, we found that the FSIQ could not be predicted from the BL full DQ despite a significant correlation between scores (rho = 0.90, −*p* = 0.037). Importantly, we found that the socialisation and adaptive behaviour scores of adolescent encephalitic children were highly correlated to toddler's BL sociability DQ scores (each rho = 0.90, −*p* = 0.037, respectively), and exhibited no intra-individual differences, both in individual pairwise comparisons and inter-agreement measures (File S2, Table S8, Panel C; Figure S1). By contrast to nonencephalitic children, head growth dynamics in encephalitic children did not correlate over time (rho = 0.51, *p* = 0.2574) despite a significant weak agreement (50%; *κ* = 0.333; *p* = 0.048) for progression within same growth corridors (File S2, Table S8, Panel C; Figure S1).

## Discussion

To the best our knowledge, the CHIK13+ cohort study is the one of the few observational studies to have evaluated the long-term neurodevelopmental outcomes of perinatal CHIKV infection. This study builds on robust methods, using standardised assessments and blind comparisons with carefully matched uninfected peers from the same epidemiological context. We evaluated the burden of long-term disabilities in CHIKV-perinatally-infected children with hindsight of 13–15 years and found that neurocognitive deficit was the most common complication, reported in 58% of infected children and matched the former definition of mental retardation in 26% of them. Neurocognitive dysfunctions involved mainly verbal comprehension and processing speed. Dysfunction could also be severely massive and involve all cognitive dimensions especially in children with encephalitis *sequelae*, those including cerebral palsy and secondary microcephaly. In adolescence age, CHIKV perinatal infection resulted in poor adaptive functioning for 26% of the infected children, which was severe in 21% and hindered communication, socialisation and, to a lesser extent, daily living skills. Lastly, 27% of infected children presented behavioural problems, 16% severe related to hyperactivity/inattention and problems in interactions with peers. Overall, poor neurodevelopmental outcomes were found in more than two thirds of the infected children, severe in 37%. In addition, infected children exhibited delayed developmental milestones in learning to walk without help or in language acquisition. They were more likely to experience sensorineural disorders in childhood, such as strabismus and reduced visual acuity. Taken together, these complications resulted in poorer autonomy, higher needs for special care and educational difficulties requiring robust support.

The interplay between higher intellectual (executive and cognitive), adaptive, and behavioural functions has long been studied in the context of child and adolescent health.^28^ In our study, there were fewer correlations between cognitive, adaptive and behavioural scores in CHIKV-perinatally-infected children than in presumably “healthy” uninfected children, which might indicate that the infection “disconnects” brain neural networks and triggers neuroplastic functional adaptations in attempt to catch up some of the neurodevelopmental deficits, as previously suggested for preterm children.^29^ Unfortunately, beyond working memory, we were unable to provide a more in-depth assessment of executive functions in this study. Previous tests by the age of ten revealed poor performances in the three core executive functions: working memory, shifting, and inhibition, and related behavioural processes, such as initiation, emotional control and planning.^17^ Thus, we postulate that the executive dysfunctions found in CHIKV-perinatally-infected children could intensify cognitive impairment and prime maladaptive and behavioural difficulties observed in adolescence, similar to what has been reported in other health conditions of childhood (FASD, attention deficits/hyperactivity disorders, very preterm birth, extremely low birthweight) affecting the prefrontal areas of the developing brain.^30^^,^^31^ This hypothesis is highly plausible in our study for at least the five aforementioned children in whom working memory, the core executive function known as the engine for learning,^32^ was particularly affected. We believe these children are prone to executive dysfunctions reminiscent of those seen in FASD or attention deficit/hyperactivity disorder.^30^^,^^33^ Interestingly, socialisation and adaptive behaviour scores of adolescent encephalitic children could be predicted from toddler's BL sociability DQ scores, which we think illustrates the early damages in the frontal lobes and prefrontal cortex, social skills being highly dependent on early infant interactions under the rule of these brain regions.^18^ The predominance of frontal lobe and prefrontal cortex damage has been shown in early MRI studies of chikungunya neonatal encephalitis,^1^^,^^6^ and anatomic correlates with related neurocognitive functions evidenced in previous study.^17^ Of note, the head growth dynamic in encephalitic children followed a dissimilar pattern during infancy and childhood compared to that observed in nonencephalitic children, which could indicate that encephalitic lesions damaged the brain at an early stage and that neuroplasticity attempted to compensate for this in order to maintain growth in the expected range. Indeed, this would be consistent with the model of neuroplasticity, according to which the most critical period for brain damage occurs between the end of pregnancy and the first month of life.^18^

The CHIK13+ cohort study was conducted in a unique regional context, and despite providing a clear representative picture of local events, it only covered two waves of a single chikungunya outbreak caused by the CHIKV East Central South African (ECSA) genotype and by its ECSA-diverged Indian ocean lineage (IOL), sucessively.^34^ That being said, although differences in virulence of the CHIKV circulating strains have been suggested, at least for chronic chikungunya manifestations,^35^ IOL being the most likely to triggering joint inflammation,^36^ ECSA least likely, and Asian genotype midway^34^; CHIKV neonatal encephalitis has been reported in almost all regions worldwide, including the Caribbean^12^ and Latin America,^13^ where the CHIKV Asian genotype, a potential neuropathogen,^37^ caused outbreaks in 2013–2014. Together with the findings in the literature, our analysis suggests that the results of this study could be potentially generalisable to a global scale, regardless of the CHIKV circulating strain. Indeed, in Africa, climate change is shifting the disease burden from malaria to arboviruses.^38^ For instance, in recent years, neurochikungunya has outreached cerebral malaria and bacterial meningitis to become the most frequent neuroinfection in Kenyan children admitted to the hospital.^39^ With absolute risks of 50% MTCT and up to 36% encephalitis/encephalopathy,^1^^,^^6^ in the absence of validated interventions to mitigate these risks, the potential harms of CHIKV perinatal infection should not be neglected in both endemic and new epidemic settings.

This study has several strengths. First, despite the CHIK13+ cohort gathering half of CHIKV-perinatally-infected children and half of CHIKV neonatal encephalitis/encephalopathy cases observed during the epidemic,^1^^,^^6^ it was representative of the target population, enabling a broad spectrum of disabilities resulting from CHIKV perinatal MTCT to be studied.^6^^,^^11^^,^^17^ Second, exposure was defined with certainty from prospective cohorts with the CHIKV genome or CHIKV-specific IgM antibodies,^1^^,^^3^ and it was also confirmed retrospectively using CHIKV-specific IgG antibodies at the time of assessment. This precaution likely abrogated misclassification bias in the groups compared. Third, we excluded competing conditions and used propensity score matching^19^^,^^20^ to equilibrate the confounders. This proved effective for balancing the matching criteria under acceptable conditions and almost effective in equalising the distributions of several environmental, maternal, neonatal (other than the matching criteria) and child characteristics at follow-up (other than those caused by the infection). Fourth, neurodevelopmental evaluation was performed blind of exposure status by a skilled neuropsychologist who had not previously assessed the children. We believe this reduced the possibility of evaluation bias, although we cannot fully eliminate unintended communications which could have broken the blind, the infected children being known to many caregivers.

The study has several potential limitations. First, enrolment and assessment took place during the COVID-19 pandemic, and we cannot ensure the disruption to follow-up in the CHIMERE cohort or the non-participation after matching in the CHIK13+ study, was not medically or socially driven, which could have underlying unmeasured confounders.^40^ Moreover, even though it aims to minimise residual confounding, propensity score matching does not supplant randomisation nor completely avoid confounding. Second, despite issued from a large cohort of CHIKV-perinatally-infected children, our sample size might seem relatively small, and in this instance, the medium to large standardised mean differences (>0.1) for four of the six matching criteria could have not ensured a perfect balance between infected and uninfected groups (*i.e.*, matching adequacy), leaving room for potential selection biases. However, this standpoint is far from being unanimously accepted, especially in small matched samples for which medium standardised mean differences could still be consistent with the propensity-score model having been correctly specified.^41^ Third, we lacked statistical power to evidence subtle differences between the groups in several subscales, which might have been the source of information bias related to secondary outcomes, especially in VABS-II subscales. Thus, statistical significance was more easily obtained by pooling children with age-appropriate skills with the least affected children and by comparing them in both groups to the children with the most severe involvement, illustrating the impact of CHIKV encephalitis on the overall findings. This should encourage researchers to prioritise matching options with more controls. Fourth, this study is single-center and may not fully capture the full spectrum of consequences of perinatal CHIKV infection.

In conclusion, the CHIK13+ cohort study shed light on the long-lasting disabilities of neonatal chikungunya encephalitis/encephalopathy, the slowdown in brain growth and the poor neurodevelopmental outcomes in adolescent children with CHIKV perinatal infection. Childcare professionals and public health decision-makers should be aware of these complications to warrant the neuroprotection of neonates in endemic settings and future chikungunya epidemics with the aim to prevent intellectual disability and related social issues.

In this perspective, this study will also inform the development of vaccines and immunotherapies for women of childbearing age, and long-term follow-up of infected neonates. Finally, this study may also endorse multifaceted interventions combining prolonged cognitive behavioural therapy, tailored education, and special needs support to alleviate the long-term impact of disabilities in children perinatally infected with chikungunya.

## Contributors

RS, BB, and PG conceived the study. RS, MOM, and PG designed the study, together with SM. PG wrote the sampling plan, matched the cohort and provided the list of eligible subjects to be enrolled. RS informed participants and their parents and enrolled participants. RS, MOM, MC, MR, CM, MB, and BB collected the data. PG validated the data with the help of BB and RS and did the statistical analysis. MOM, MC, BB, and PG interpreted the data. RS and PG wrote the manuscript which was then revised by all authors. All authors approved the final version of the manuscript. All authors had full access to all the data in the study, agreed to be accountable for all the aspects of the work and took responsibility for the decision to submit the publication. PG acted as data guarantor.

## Data sharing statement

According to CHIMERE cohort data access policy, requests to access the dataset used for this study can be sent to the corresponding author and data guarantor (PG). These requests will be reviewed promptly for confidentiality, data protection, and intellectual property, and will not be unreasonably denied. These will include access to de-identified data, data dictionary, study protocol, statistical plan and inform consent from upon the signature of a data sharing agreement.

## Declaration of interests

All authors declare that they have no conflicts of interest related to this work.

## Figures and Tables

**Supplementary Figure:**
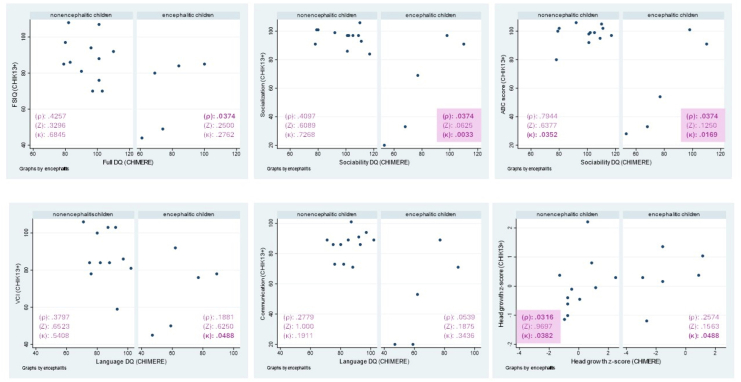
BL: Brunet Lézine. DQ: development quotient. FSIQ: full scale intelligence quotient. VCI: verbal comprehension index. ABC: adaptive behavior composite. ∗Correlations between scores are estimated using Spearman rho (ρ) coefficients. ∗∗Scores are compared as pairwise repeated measures using Wilcoxon signed-rank tests (z). Interagreement between score categories is assessed using Cohen’s kappa (Ϗ).

## Appendix A Supplementary data

File S2

Protocole
