# Supplementary material for: Long-term neurodevelopmental outcomes of children perinatally infected with chikungunya: the CHIK13+ matched cohort study on Reunion Island
Source: eClinicalMedicine. 2026 May 18;95:103975. doi: 10.1016/j.eclinm.2026.103975 (PMC13208087; doi:10.1016/j.eclinm.2026.103975)
Supplement: File S1 [file mmc1.docx]

**Methodological appendix (full detail of methods)**

**Long-term neurodevelopmental outcomes of children perinatally infected with chikungunya: the CHIK13+ matched cohort study on Reunion Island**

Raphaëlle Sarton, MD,^1^ Marie Odile Mery, MSc,^2^ Magali Carbonnier, MD,^2^ Michel Renouil, MD^1^, Camille Morice, MD,^3^ Samir Medjane, PhD,^4^ Marc Bintner, MD,^5^ Brahim Boumahni, MD,^6^ Patrick Gérardin, MD^7,8 ,^*

^1^ Department of Paediatrics, Centre Hospitalier Universitaire (CHU) de La Réunion, Saint Pierre, Reunion, France

^2^ Centre d’Action Médico-Sociale Précoce Christian Isautier, Fondation Père Favron, Saint Louis, Reunion, France

^3^ Department of Ophtalmology, CHU Réunion, Saint Pierre, Reunion, France

^4^ Direction de la Recherche Clinique et de l’Innovation, CHU Réunion, Saint Pierre, Reunion, France

^5^ Neuroradiology department, CHU de La Réunion, Saint Pierre, Reunion, France

^6^ Department of Neonatology, CHU Réunion, Saint Pierre, Reunion, France

^7^ Centre for Clinical Investigation Clinical Epidemiology (CIC1410), Institut National de la Santé et de la Recherche Médicale (INSERM), CHU Réunion, Saint Pierre, Reunion, France

^8^ Plateforme de Recherche Clinique et Translationnelle, CHU Réunion, Saint Pierre, Reunion, France

* Corresponding author :

Dr Patrick Gérardin, Centre for Clinical Investigation Clinical Epidemiology, Inserm CIC1410, CHU Réunion, Site du GHSR, av. de Soweto, BP 350, 97448 Saint Pierre Cedex, Reunion

Email : [patrick.gerardin@chu-reunion.fr](mailto:patrick.gerardin@chu-reunion.fr) ; Tel : +262 62 35 94 35

Short running head title: Neurodevelopmental outcomes of perinatal chikungunya

**Methods**

**Study design and participants**

CHIK13+ is an ambispective, observational, matched cohort study in which CHIKV exposure was defined retrospectively and outcomes were assessed prospectively after the thirteenth birthday. All children born and infected through CHIKV perinatal mother-to-child transmission (MTCT) during the 2005-2006 outbreak were eligible for the study. This included the children that participated to the neurodevelopment assessment at the age of two in the CHIMERE cohort study,^1,2^ and additional children from the same background diagnosed at the time of the epidemic taken from the hospital-based historical cohorts.^3,4^

Prior to enrolment in the cohort, CHIKV-perinatally-infected children were listed and matched in equal proportions (1:1) with uninfected peers on multiple potential confounding factors: maternal age, maternal education level, month of birth, gender, gestational age, and birthweight using Mahalanobis distance matching within propensity score calipers,^5,6^ from a pre-established list of eligible children taken from same maternity birth registers. Each infected child was assigned up to six potential uninfected peers who were offered to participate in the study. Uninfected children could have been exposed perinatally to maternal CHIKV viremia without contracting the infection. In summary, eligible uninfected controls had to be born in the same epidemic context and to share the six matching criteria.

The selection of control children was based on the willingness to participate activated by the enrollment of the infected child. For each participating infected child, the list of potential uninfected controls was reviewed in the pre-established order, and the first control child in the list who agreed to participate was included to form the pair. If the control child had already been selected in another pair, the next child on the list was proposed to participate, and so on.

Children with pre-existing conditions likely to hamper the normal acquisition of developmental milestones and neurodevelopmental scores were not eligible for the study. Non-inclusion criteria were, as follows: very preterm births less than 33 weeks gestation, fetal alcohol spectrum disorders (FASD), autism spectrum disorders (ASD), infantile psychosis and intellectual disability or epilepsy stemming from causes other than neurochikungunya.

**Ethics approval**

The parents of children who formerly participated in the CHIMERE cohort had been informed for potential further follow-up,^2^ and provided written consent. CHIKV-Infected children not enrolled in this cohort were monitored by pediatricians in a standard care setting and were regularly informed of new research on perinatally-acquired chikungunya.

For the CHIK13+ cohort study, all parents received new verbal and written information on study’s aims, constraints, expected risks and benefits. Children were asked to participate only if parents had given consent. Both parents and their child were asked for written consent.

Parents of eligible children were contacted when their child reached 13 years of age. In order to minimize impacts on education, infected-uninfected pair enrolment was randomly planned during school vacations or non-teaching days according to the school calendar.

The study was in accordance with the MR-001 of the French National Commission for Informatics and Liberty (CNIL). Following the French regulation (Loi Jardé 2019), it was approved by the *Comité de Protection des Personnes (CPP) Sud-Ouest et Outremer 4* (IRB: IORG0009855) as an interventional study on human subjects with minimal risks and constraints (RIPH2) under the reference L5311-1-CPP2019-09-072 and registered in both the French National Agency for Drug Safety (ANSM) database (IDRCB: 2019-A02095-52) and the Clinical Trials register (NCT04909411).

**Exposure**

Exposure had been defined at birth in perinatally-infected neonates by the presence of viral RNA using a positive blood or cerebrospinal fluid RT-PCR or by the presence of CHIKV-specific IgM antibodies in the serum.^3,4^ This was also verified at the time of enrolment in the CHIK13+ cohort by the presence of CHIKV-specific IgG antibodies. A check was necessary to exclude from the CHIKV-infected group: 1- children who had been exposed before or during delivery, in whom, in the absence of maternal-fetal or postnatal infection, transplacental CHIKV-specific IgG antibodies had been eliminated within 32 months;^7^ 2- children who had not been exposed before or during delivery, but who could have been infected during early childhood over the epidemic, as it is also known that early infection during early childhood has adverse effects on neurological development.^8,9^ Testing, both at the time of the epidemic and at the time of enrolment ensured correct exposure classification within the study. In summary, double testing allowed exposure to be graded with certainty based on virological (CHIKV genome in blood or cerebrospinal fluid) or serological testing (CHIKV-specific IgM abs) in the perinatal period and confirmed at the time of enrolment in the study (CHIKV-specific IgG abs).

**Outcomes**

The primary endpoint was the full-scale intelligence quotient (FSIQ) of the Wechsler Intelligence Scale for Children® fifth edition (WISC-V) which provides an overall rating of cognitive performance.^10^ This scale assesses verbal comprehension, visual spatial skills, fluid reasoning, working memory and processing speed. For the FSIQ and each of its indices, cognitive deficit was deemed severe for scores of 55 or less (≤ -3 standard deviations; < 1^st^ centile), moderate for scores of 56-70 (-3 to ≤ -2 SD; 1^st^ to 3^rd^ centile), mild for scores 71-85 (-2 to ≤ -1 SD; 3^rd^ to 16^th^ centile) or absent for scores above 85. A FSIQ of 70 or less (≤-2 SD) indicated intellectual disability (DSM-V).

Secondary endpoints included the adaptive behavior composite (ABC) score of the Vineland Adaptive Behavior Scale second edition (VABS-II),^11^ and the total difficulties score of the Strengths and Difficulties Questionnaire (SDQ).^12^

The VABS-II is a multipurpose tool aimed at assessing the adaptive behaviour of children through three different scales exploring communication, daily living, and socialization (and incidentally also gross and fine motor skills). Scores less than 70 (≤ -2 SD) indicate a very low adaptive behaviour, 71–85 (-2 to ≤ -1 SD) a low adaptive behaviour, and scores higher than 85 (> -1 SD) an average adaptive behaviour.^11^

The SDQ is a questionnaire that assesses behaviour through five different scales exploring emotional symptoms, conduct problems, hyperactivity/inattention, relationships with peers and prosocial behaviour. It can be used for clinical assessment either through child, parent, or teacher reports. The sum of its first four dimensions yields the SDQ total difficulties score which can be used to diagnose behavioural problems. A SDQ of 14-16 (1 to ≤ 2 SD) defines mild to moderate difficulties, and a score higher than 16 high difficulties (> 2 SD).^12^

We also assessed a composite binary indicator of all identified impairments in the three abovementioned scales, as done in the French MONALISA cohort.^13^ In this, poor neurodevelopmental outcome was defined as a cluster a FSIQ and/or ABC score ≤ 85 and/or SDQ total difficulties score higher than 13. Severe neurodevelopmental impairment was defined at least as one of the abovementioned scores less than 70 or higher than 16 and as a proxy indicator of intellectual disability (DSM-V).

Subsidiary intermediate outcomes included assessing developmental delays such as walking without help or language (phonemes and sentences), sensorineural disorders (visual or hearing deficit, wearing correctional lenses, hearing aids), special needs (speech, psychomotor, or physiotherapist), schooling information (repeated grades, mainstream class with or without support, special education and home care service), and various health issues (cerebral palsy, swallowing and sleep disorders), emergency consultations and hospitalizations. These outcomes were obtained through examination of the child’s health record and parental interviews.

The first assessment visit included a non-blinded physical examination by a pediatrician, an interview with the parents, serological assay (ELISA IgG and IgM immunocapture), and questionnaires for parents, children, and teachers. Ophthalmological examinations *(i.e.*, visual acuity, fundus, and orthoptist evaluations) and neuropsychological assessment by a single skilled neuropsychologist (MOM) were performed during the school year. The ophthalmologist and neuropsychologist were blinded to CHIKV exposure.

**Covariates**

Neonatal characteristics were retrieved from maternity birth registers and linked with previous cohort data,^1-4^ This allowed to distinguish different phenotypes in CHIKV-perinatally-infected children : encephalitic *versus* non-encephalitic; small-headed (< 1 SD or < 16^th^ centile) *versus* normal-sized head (≥ 16^th^ centile); positive *versus* negative head growth dynamics (*i.e.*, centile gain *vs* centile loss).^14^

The data collected included parental professions and highest socio-professional category in the parental couple, the deprivation index as provided by borough of residence, number of siblings, sibling school grades, maternal education level, pregnancy (parity, gestational diabetes, pregnancy-induced hypertension disorders) and perinatal variables (gestational age, birth height, birthweight, head circumference, low birthweight and small for gestational age, Apgar scores), the medical history of the child, breastfeeding, and growth parameters (height, weight, head circumference, growth dynamics, stunting, wasting, overweight and obesity) based on Center for Disease Control and Prevention growth charts.

**Statistical analysis**

Sample size was guided by prior knowledge of the recruitment capacity. We anticipated that of the 33 CHIKV-perinatally-infected children assessed in the CHIMERE cohort, we could enroll roughly two thirds in the new cohort, as done previously.^15^ Each CHIKV-perinatally-infected child was matched with one uninfected child using psmatch2 in Stata and the method of k-nearest neighbor to form a pair. Matching adequacy was checked using both the standardized mean difference (SMD, aka Cohen’s δ effect size) and the variance ratio.^16^ Given the unreliability of the SMD to diagnose imbalance in small samples,^17^ imbalance between infected and uninfected groups was defined for matching criteria and other factors if their SMD was large (>0.8) or more than small (>0.1) with a variance ratio significantly different than 1.

To provide a clinically relevant difference of 1 standard deviation (15 points) in the FSIQ, 21 pairs would yield a statistical power of 88% in two-tailed hypothesis, with an α risk of 5%. This sample size would allow us to show similar differences in WISC-V and VABS-II scales.

The association between exposure and neurodevelopmental outcomes was assessed in multivariate propensity score matched analyses^5,6^ from a causal inference perspective.^18^ We designed two series of analyses: 1-the primary analysis of appropriately matched children; 2- the secondary analysis of all enrolled children (matched or not), taken as a tight sensitivity analysis. In the article, we present the primary analyses. In these, we first compared means using Wilcoxon signed-rank tests. We next compared proportions using Mac Nemar tests. For binary outcomes that were significant, we measured effect sizes using logistic regression and conditional Poisson regression (or generalized estimating equations) models with the matched pair as grouping variable, odds ratios or risk ratios (relative risks) as outputs.

Sensitivity analyses are presented as supporting information. In these, proportions were compared using Chi2 or Fisher exact tests and non-conditional logistic regression models, means using Student-T or Mann-Whitney-Wilcoxon tests, and medians using a non-parametric Brown-Mood tests, as appropriate.

Subscales scores were correlated together using Spearman rho (ρ) coefficients.

Finally, we conducted a longitudinal analysis among the infected children on similar IQ scales to test whether the cognitive development continued to change over time between the previous assessment at two years of age in the CHIMERE cohort and the new assessment at adolescence age in the CHIK13+ cohort. To this end, we considered that a long-term outcome could be predicted from an early measure if it met three criteria: a significant correlation between the two score distributions, the absence of a significant score difference in individual pairwise comparisons, and a higher than expected agreement between score classifications (*i.e.*, absence of individual significant change and progression within same standard deviations corridor of performances throughout development). In this, correlations between the two measure distributions were tested using Spearman rho (ρ) coefficients, the difference between two similar pairwise measures using Wilcoxon signed-rank tests, and inter-agreement between the two score categories using Cohen’s kappa (κ) tests. Briefly, the scales that could be compared longitudinally were: 1- the full development quotient (DQ) score of the revised Brunet-Lézine (BL) scale (CHIMERE) to the FSIQ score (CHIK13+); 2- the BL language DQ score (CHIMERE) to the verbal comprehension index of the WISC-V or the communication score of the VABS-II (CHIK13+); 3- the BL sociability DQ score (CHIMERE) to the socialization index or the ABC of VABS-II (CHIK13+). We also compared the z-scores of head circumferences and of head growth dynamics between birth and two-year to those between two-year and 14-year assessments.

Analyses were performed with Stata® (v16·1, StataCorp, College Station, Tx, USA, 2019). For all of these, observations with missing data were eliminated and a two-tailed *p* less than 0·05 was considered significant in first instance. However, for the specific analyses of percentages of deficits (normal *versus* any deficit, normal or mild to moderate deficits *versus* severe deficits), we adjusted the second test by a Bonferroni correction, and its statistical significance was set at *p*=0·025 (bilateral formulation) to account for inflation of alpha risk due to multiple testing.

**Role of the funding source**

The funder of the study had no role in study design, data collection, data analysis, data interpretation, or writing of the report.

**Reporting**

The study’s reporting the results of this study adhered to the STROBE guidelines, as is generally the case for observational studies that do not compare drugs or non-pharmacological interventions, but merely compare groups based on their exposure to a disease.

**References**

1. Fritel X, Rollot O, Gérardin P, et al. Chikungunya virus infection during pregnancy, Réunion, France, 2006. *Emerg Infect Dis* 2010; 16: 418-25.
2. Gérardin P, Sampériz S, Ramful D, et al. Neurocognitive outcome of children exposed to perinatal mother-to-child Chikungunya virus infection: the CHIMERE Cohort Study on Reunion Island. *PLoS Negl Trop Dis* 2014; 8: e2996.
3. Gérardin P, Barau G, Michault A, et al. Multidisciplinary prospective study of mother-to-child chikungunya virus infections on the island of La Réunion. *PLoS Med* 2008; 5: e60.
4. Ramful D, Carbonnier M, Pasquet M, et al. Mother-to-child transmission of Chikungunya virus infection. *Pediatr Infect Dis J* 2007; 26: 811-5.
5. Austin PC. A comparison of 12 algorithms for matching on the propensity score. *Stat Med* 2014; 33: 1057-69.
6. Austin PC. Optimal caliper widths for propensity-score matching when estimating differences in means and differences in proportions in observational studies. *Pharm Stat* 2010; 10: 150-61.
7. Ramful D, Sampériz S, Fritel X, et al. Antibody kinetics in infants exposed to Chikungunya virus infection during pregnancy reveals absence of congenital infection. *J Infect Dis* 2014; 209: 1726-30.
8. Ferreira FCPADM, da Silva ASV, Recht J, et al. Vertical transmission of& Chikungunya virus: a systematic review. *PLoS One* 2021; 16: e0249166.
9. Van Ewijk R, Huibers MHW, Manshande ME, et al. Neurologic sequelae of severe chikungunya infection in the first 6 months of life: a prospective cohort study 24-months post-infection. *BMC Infect Dis* 2021; 21: 179.
10. Canivez GL, Watkins MV, Dombrowski SC. Structural validity of the Wechsler Intelligence Scale for Children-Fifth edition: confirmatory factor analyses with 16 primary and secondary subtests. *Psychol Assess* 2017; 29: 458-72.
11. Sparrow SS, Cicchetti DV, Balla DA, Doll EA. Vineland adaptive behavior scales Vineland-II (2^nd^ ed.): Survey forms manual. American Guidance Service; Circle Pines, MN: NCS Pearson 2005.
12. Goodman R. The Strengths and Difficulties Questionnaire: a research note. *J Child Psychol Psychiatr* 1997; 38: 581-6.
13. Charlier C, Barrault Z, Rousseau J, et al. Long-term neurological and neurodevelopmental outcome of neonatal Listeriosis in France: a prospective, matched observational cohort study. *Lancet Child Adolesc Health* 2023; 7: 875-85.
14. Sarton R, Medina-Santos R, Boumahni B, Gauthier P, Renouil M, Medjane S, et al. Impaired brain growth in children perinatally infected with chikungunya. *Pediatr Neuro*l 2026; April 28. Epub ahead of print. DOI: [10.1016/j.pediatrneurol.2026.04.023](https://doi.org/10.1016/j.pediatrneurol.2026.04.023).
15. Sarton R, Carbonnier M, Robin S, et al. Perinatal mother-to-child chikungunya virus infection: screening of cognitive and learning difficulties in a follow-up study of the CHIMERE cohort on Reunion Island. *Viruses* 2025; 17: 704.
16. Zhang A, Kim HJ, Lonjon G, et al. Balance diagnostics after propensity score matching. *Ann Transl Med* 2019; 7: 16.
17. Austin PC. Balance diagnostics for comparing the distribution of baseline covariates between treatment groups in propensity-score match samples. Stat Med 2009; 28: 3083-3107.
18. Hernan MA, Hsu J, Healy B. A second chance to get causal inference right: a classification of data science tasks. *Chance* 2019; 32: 42-9.
