# Supplementary material for: Long-term neurodevelopmental outcomes of children perinatally infected with chikungunya: the CHIK13+ matched cohort study on Reunion Island
Source: eClinicalMedicine. 2026 May 18;95:103975. doi: 10.1016/j.eclinm.2026.103975 (PMC13208087; doi:10.1016/j.eclinm.2026.103975)
Supplement: Tables S1–S6 [file mmc2.docx]

**Supporting information**

**Long-term neurodevelopmental outcomes of children perinatally infected with chikungunya: the CHIK13+ matched cohort study on Reunion Island**

Raphaëlle Sarton, MD,^1^ Marie Odile Mery, MSc,^2^ Magali Carbonnier, MD,^2^ Michel Renouil, MD^1^, Camille Morice, MD,^3^ Samir Medjane, PhD,^4^ Marc Bintner, MD,^5^ Brahim Boumahni, MD,^6^ Patrick Gérardin, MD, PhD^7,8 ,^*

^1^ Department of Paediatrics, Centre Hospitalier Universitaire (CHU) de La Réunion, Saint Pierre, Reunion, France

^2^ Centre d’Action Médico-Sociale Précoce Christian Isautier, Fondation Père Favron, Saint Louis, Reunion, France

^3^ Department of Ophtalmology, CHU Réunion, Saint Pierre, Reunion, France

^4^ Direction de la Recherche Clinique et de l’Innovation, CHU Réunion, Saint Pierre, Reunion, France

^5^ Neuroradiology department, CHU de La Réunion, Saint Pierre, Reunion, France

^6^ Department of Neonatology, CHU Réunion, Saint Pierre, Reunion, France

^7^ Centre for Clinical Investigation Clinical Epidemiology (CIC1410), Institut National de la Santé et de la Recherche Médicale (INSERM), CHU Réunion, Saint Pierre, Reunion, France

^8^ Plateforme de Recherche Clinique et Translationnelle, CHU Réunion, Saint Pierre, Reunion, France

* Corresponding author :

Dr Patrick Gérardin, Centre for Clinical Investigation Clinical Epidemiology, Inserm CIC1410, CHU Réunion, Site du GHSR, av. de Soweto, BP 350, 97448 Saint Pierre Cedex, Reunion

Email : [patrick.gerardin@chu-reunion.fr](mailto:patrick.gerardin@chu-reunion.fr) ; Tel : +262 62 35 94 35

Short running head title: Neurodevelopmental outcomes of perinatal chikungunya

Supplemental tables: 8. 6 .doc tables and 1 Excel file (2 additional tabs).

**Keywords**: arbovirus, chikungunya, congenital infection, encephalitis, neurodevelopment, cohort study

| **Supplementary Table 1. Characteristics of 37 perinatally CHIKV-infected children that survived the neonatal period, CHIK13+, Reunion island, 2020-2021.** | | | | | | | |
| --- | --- | --- | --- | --- | --- | --- | --- |
| **Exposure group** | **Total** | | **Infected children**  **non participants** | | **Infected children participants** | |  |
|  | n = 37 | (%) | n = 18 | (%) | n = 19 | (%) | *p value* |
| ***Environmental characteristics*** |  |  |  |  |  |  |  |
| **Neighbourhood deprivation** |  |  |  |  |  |  |  |
| Absent | 7 | (18.9) | 2 | (11.1) | 5 | (26.3) | 0.519 |
| Moderate | 12 | (32.4) | 7 | (38.9) | 5 | (26.3) |  |
| High | 18 | (48.7) | 9 | (50.0) | 9 | (47.4) |  |
| ***Maternal characteristics*** |  |  |  |  |  |  |  |
| **Age** * (years; median, Q_1_-Q_3_) | 28.0 | (23.0 - 33.0) | 27.0 | (19.0 - 31.0) | 28.2 | (24.3 - 36.9) | 0.892 |
| **Education *** |  |  |  |  |  |  |  |
| Lower than college | 6 | (16.2) | 2 | (11.1) | 4 | (21.1) | 0.353 |
| Lower than baccalaureate | 11 | (29.7) | 4 | (22.2) | 7 | (36.8) |  |
| Baccalaureate or higher | 20 | (54.1) | 12 | (66.7) | 8 | (42.1) |  |
| **Parity** |  |  |  |  |  |  |  |
| 0 | 11 | (30.5) | 5 | (29.4) | 6 | (31.6) | 0.901 |
| 1-3 | 20 | (55.6) | 9 | (52.9) | 11 | (57.9) |  |
| ≥ 4 | 5 | (13.9) | 3 | (17.7) | 2 | (10.5) |  |
| **Prepregnancy mass index** |  |  |  |  |  |  |  |
| (kg/m^2^; median, Q_1_-Q_3_) | 24.7 | (22.1 - 28.9) | 25.2 | (22.5 - 28.0) | 24.0 | (21.7 - 29.9) | 0.739 |
| < 25 | 19 | (52.8) | 9 | (50.0) | 10 | (55.6) | 1.000 |
| 25-29.9 | 9 | (25.0) | 5 | (27.8) | 4 | (22.2) |  |
| ≥ 30 | 8 | (22.2) | 4 | (22.2) | 4 | (22.2) |  |
| **Smoking during pregnancy** |  |  |  |  |  |  | 0.340 |
| No | 33 | (89.2) | 15 | (83.3) | 18 | (94.4) |  |
| Yes | 4 | (10.8) | 3 | (16.7) | 1 | (5.3) |  |
| ***Child characteristics at birth*** |  |  |  |  |  |  |  |
| **Gender *** |  |  |  |  |  |  |  |
| Male | 23 | (62.2) | 10 | (56.6) | 13 | (68.4) | 0.420 |
| Female | 14 | (37.8) | 8 | (44.4) | 6 | (31.6) |  |
| **Gestational age ***(weeks; median, Q_1_-Q_3_) | 38 | (37 - 39) | 38 | (37 - 39) | 38 | (37 - 39) | 0.833 |
| **Preterm birth** |  |  |  |  |  |  |  |
| No | 33 | (89.2) | 17 | (94.4) | 16 | (84.2) | 0.604 |
| Yes | 4 | (10.8) | 1 | (5.6) | 3 | (15.8) |  |
| **Birthweight *** (kg; median, Q_1_-Q_3_) | 3.10 | (2.76 - 3.34) | 3.07 | (2.76 - 3.40) | 3.10 | (2.62 - 3.34) | 0.866 |
| **Low birthweight** |  |  |  |  |  |  |  |
| No | 32 | (86.5) | 16 | (84.2) | 16 | (84.2) | 1.000 |
| Yes | 5 | (13.5) | 2 | (15.8) | 3 | (15.8) |  |
| **Height at birth** (cm; median, Q_1-_Q_3_) | 49 | (47 - 50) | 49 | (48 - 51) | 48 | (46 - 50) | 0.640 |
| **Small for gestational age** |  |  |  |  |  |  |  |
| No | 29 | (78.4) | 14 | (77.8) | 15 | (79.0) | 0.413 |
| 10^th^-3^rd^ centile | 6 | (16.2) | 4 | (22.2) | 2 | (10.5) |  |
| < 3^rd^ centile | 2 | (5.4) | 0 | (0.0) | 2 | (10.5) |  |
| **Head circumference** (cm; median, Q_1_-Q_3_) | 34 | (33 - 35) | 34 | (33 - 35) | 34 | (32 - 35) | 0.569 |
| Normal head, -1 SD ≤ to ≤ +2 SD | 29 | (78.4) | 15 | (83.3) | 14 | (73.7) | 0.405 |
| Small head, -2 SD ≤ to < - 1 SD | 7 | (18.9) | 2 | (11.1) | 5 | (26.3) |  |
| Microcephaly, < - 2 SD | 1 | (2.7) | 1 | (5.6) | 0 | (0.0) |  |
| **Apgar score at 1 minute** |  |  |  |  |  |  |  |
| 10 | 30 | (81.1) | 15 | (83.3) | 15 | (78.9) | 0.694 |
| 7-9 | 3 | (8.1) | 2 | (11.1) | 1 | (5.3) |  |
| < 7 | 4 | (10.8) | 1 | (5.6) | 3 | (15.8) |  |
| **Breastfeeding** |  |  |  |  |  |  |  |
| No | 19 | (52.8) | 9 | (52.9) | 10 | (52.6) | 0.985 |
| Yes | 17 | (47.2) | 8 | (47.1) | 9 | (47.4) |  |
| ***Characteristics of exposure*** |  |  |  |  |  |  |  |
| **Month of birth in the epidemic** (median, Q_1_-Q_3_) | 11 | (11 - 12) | 11.5 | (11 - 12) | 11 | (9 - 12) | 0.879 |
| **Epidemic waves** |  |  |  |  |  |  |  |
| 1 (March to August 2005) | 4 | (17.8) | 1 | (5.6) | 3 | (15.8) | 0.604 |
| 2 (September 2005 to August 2006) | 33 | (89.2) | 17 | (94.4) | 16 | (84.2) |  |
| Data are numbers and column percentages, or medians and interquartile ranges (Q1-Q3) when specified. Percentages are compared using Chi2 or Fisher’s Exact test, as appropriate. Medians are compared using a non-parametric Brown-Mood test. | | | | | | | |

| **Supplementary Table 2. Characteristics of 4777 uninfected children that survived the neonatal period and could serve as potential controls, CHIK13+, Reunion island, 2020-2021.** | | | | | | | |
| --- | --- | --- | --- | --- | --- | --- | --- |
| **Exposure group** | **Total** | | **Uninfected children**  **nonparticipants** | | **Uninfected children participants** | |  |
|  | n = 4777 | (%) | n = 4758 | (%) | n = 19 | (%) | *p value* |
| ***Environmental characteristics*** |  |  |  |  |  |  |  |
| **Neighborhood deprivation** |  |  |  |  |  |  |  |
| Absent | 1799 | (37.7) | 1795 | (37.7) | 4 | (21.1) | 0.139 |
| Moderate | 1858 | (38.9) | 1851 | (38.9) | 7 | (36.8) |  |
| High | 1120 | (23.4) | 1112 | (23.4) | 8 | (42.1) |  |
| ***Maternal characteristics*** |  |  |  |  |  |  |  |
| **Age** * (years; median, Q_1_-Q_3_) | 28.0 | (23.0 - 33.0) | 28.0 | (23.0 - 33.0) | 30.2 | (24.7 - 34.2) | 0.160 |
| **Education *** |  |  |  |  |  |  |  |
| Lower than college | 244 | (5.7) | 241 | (5.7) | 3 | (15.8) | 0.125 |
| Lower than baccalaureate | 1920 | (45.2) | 1911 | (45.2) | 9 | (47.4) |  |
| Baccalaureate or higher | 2087 | (49.1) | 2080 | (49.1) | 7 | (36.8) |  |
| **Parity** |  |  |  |  |  |  |  |
| 0 | 1867 | (39.1) | 1860 | (39.1) | 7 | (36.8) | 0.585 |
| 1-3 | 2565 | (53.7) | 2553 | (53.7) | 12 | (63.2) |  |
| ≥ 4 | 341 | (7.1) | 341 | (7.2) | 0 | (0.0) |  |
| **Prepregnancy mass index** |  |  |  |  |  |  |  |
| (kg/m^2^; median, Q_1_-Q_3_) | 22.6 | (20.0 - 26.3) | 22.6 | (20.0 - 26.3) | 26.5 | (20.8 - 32.9) | 0.135 |
| < 25 | 3059 | (68.4) | 3050 | (68.5) | 9 | (47.4) | 0.031 |
| 25-29.9 | 885 | (19.8) | 881 | (19.8) | 4 | (21.0) |  |
| ≥ 30 | 529 | (11.8) | 523 | (11.7) | 6 | (31.6) |  |
| **Smoking during pregnancy** |  |  |  |  |  |  | 0.443 |
| No | 4229 | (88.9) | 4214 | (88.8) | 15 | (83.3) |  |
| Yes | 530 | (11.1) | 527 | (11.2) | 3 | (16.7) |  |
| ***Child characteristics at birth*** |  |  |  |  |  |  |  |
| **Gender *** |  |  |  |  |  |  |  |
| Male | 2405 | (50.4) | 2393 | (50.3) | 12 | (63.2) | 0.264 |
| Female | 2370 | (49.6) | 2363 | (49.7) | 7 | (36.8) |  |
| **Gestational age ***(weeks; median, Q_1_-Q_3_) | 39 | (38 - 40) | 39 | (38 - 40) | 38 | (38 - 39) | 0.567 |
| **Preterm birth** |  |  |  |  |  |  |  |
| No | 4345 | (91.0) | 4328 | (91.0) | 17 | (89.5) | 0.687 |
| Yes | 431 | (9.0) | 429 | (9.0) | 2 | (10.5) |  |
| **Birthweight *** (kg; median, Q_1_-Q_3_) | 3.14 | (2.83 - 3.45) | 3.14 | (2.82 - 3.45) | 3.18 | (3.03 - 3.41) | 0.604 |
| **Low birthweight** |  |  |  |  |  |  |  |
| No | 4061 | (85.0) | 4043 | (85.0) | 18 | (94.7) | 0.342 |
| Yes | 716 | (15.0) | 715 | (15.0) | 1 | (5.3) |  |
| **Height at birth** (cm; median, Q_1-_Q_3_) | 49 | (47 - 50) | 49 | (47 - 50) | 50 | (49 - 51) | 0.101 |
| **Small for gestational age** |  |  |  |  |  |  |  |
| No | 3931 | (82.3) | 3914 | (82.3) | 17 | (89.5) | 0.849 |
| 10^th^-3^rd^ centile | 725 | (15.2) | 723 | (15.2) | 2 | (10.5) |  |
| < 3^rd^ centile | 2 | (2.5) | 0 | (2.5) | 0 | (0.0) |  |
| **Head circumference** (cm; median, Q_1_-Q_3_) | 34 | (33 - 35) | 34 | (33 - 35) | 34 | (33 - 35) | 0.932 |
| Normal head, -1 SD ≤ to ≤ +2 SD | 2170 | (78.4) | 2152 | (83.3) | 18 | (94.7) | 0.353 |
| Small head, -2 SD ≤ to < - 1 SD | 468 | (17.3) | 467 | (11.1) | 1 | (5.3) |  |
| Microcephaly, < - 2 SD | 82 | (2.7) | 82 | (5.6) | 0 | (0.0) |  |
| **Apgar score at 1 minute** |  |  |  |  |  |  |  |
| 10 | 4176 | (88.3) | 4164 | (88.3) | 14 | (73.7) | 0.058 |
| 7-9 | 395 | (8.4) | 392 | (8.4) | 3 | (15.8) |  |
| < 7 | 157 | (3.3) | 155 | (3.3) | 2 | (10.5) |  |
| **Breastfeeding** |  |  |  |  |  |  |  |
| No | 1143 | (52.8) | 1137 | (52.9) | 6 | (31.6) | 0.426 |
| Yes | 3606 | (47.2) | 3593 | (47.1) | 13 | (68.4) |  |
| ***Characteristics of exposure*** |  |  |  |  |  |  |  |
| **Month of birth in the epidemic** (median, Q_1_-Q_3_) | 9 | (5 - 12) | 9 | (5 - 12) | 11 | (9 - 12) | <0.001 |
| **Epidemic waves** |  |  |  |  |  |  |  |
| 1 (March to August 2005) | 1688 | (35.3) | 1685 | (35.4) | 3 | (15.8) | 0.092 |
| 2 (September 2005 to August 2006) | 3089 | (64.7) | 3073 | (64.6) | 16 | (84.2) |  |
| Data are numbers and column percentages, or medians and interquartile ranges (Q1-Q3) when specified. Percentages are compared using Chi2 or Fisher’s Exact test, as appropriate. Medians are compared using a non-parametric Brown-Mood test. | | | | | | | |

| \| \| **Supplementary table 3. Characteristics of 42 nonmatched infected-uninfected children exposed to perinatal mother-to-child transmission of chikungunya virus, CHIK13+, Reunion island, 2020-2021.** \| \| --- \| \| \| --- \| --- \| | | | | | | | | |
| --- | --- | --- | --- | --- | --- | --- | --- | --- | --- | --- |
| **Exposure group** | **Total** | | **CHIK -** | | **CHIK +** | | |  |
|  | n = 42 | (%) | n = 23 | (%) | n = 19 | | (%) | *p value* |
| ***Environmental characteristics*** |  |  |  |  |  |  | |  |
| **Neighbourhood deprivation** |  |  |  |  |  |  | |  |
| Absent | 12 | (28.6) | 7 | (30.4) | 5 | (26.3) | | 0.701 |
| Moderate | 13 | (30.9) | 8 | (34.8) | 5 | (26.3) | |  |
| High | 17 | (40.5) | 8 | (34.8) | 9 | (47.4) | |  |
| **Higher socio-professional category of parents** | | |  |  |  |  | |  |
| Executive and upper professions | 3 | (7.3) | 2 | (9.1) | 1 | (5.3) | | 0.720 |
| Intermediate | 8 | (19.5) | 6 | (27.3) | 2 | (10.5) | |  |
| Farmers, craftsmen, entrepreneurs | 10 | (24.4) | 4 | (18.2) | 6 | (31.6) | |  |
| Employees | 0 | (0.0) | 0 | (0.0) | 0 | (0.0) | |  |
| Workers | 5 | (23.2) | 3 | (13.6) | 2 | (10.5) | |  |
| Pensioners | 12 | (29.3) | 6 | (27.3) | 6 | (31.6) | |  |
| Unemployed | 3 | (7.3) | 1 | (4.5) | 2 | (10.5) | |  |
| **Other siblings at home** |  |  |  |  |  |  | |  |
| 0 | 4 | (9.5) | 3 | (13.0) | 1 | (5.3) | | 0.812 |
| 1-2 | 17 | (40.5) | 9 | (39.1) | 8 | (42.1) | |  |
| ≥ 3 | 21 | (50.0) | 11 | (47.8) | 10 | (52.6) | |  |
| ***Maternal characteristics*** |  |  |  |  |  |  | |  |
| **Age** * (years; median, Q_1_-Q_3_) | 28.6 | (24.3 - 34.7) | 29.7 | (24.1 - 34.2) | 28.2 | (24.3 - 36.9) | | 0.535 |
| **Education *** |  |  |  |  |  |  | |  |
| Lower than college | 7 | (17.1) | 3 | (13.6) | 4 | (21.1) | | 0.840 |
| Lower than baccalaureate | 16 | (39.0) | 9 | (40.9) | 7 | (36.8) | |  |
| Baccalaureate or higher | 18 | (43.9) | 10 | (45.5) | 8 | (42.1) | |  |
| **Parity** |  |  |  |  |  |  | |  |
| 0 | 14 | (33.3) | 8 | (34.8) | 6 | (31.6) | | 0.443 |
| 1-3 | 26 | (61.9) | 15 | (65.2) | 11 | (57.9) | |  |
| ≥ 4 | 2 | (4.8) | 0 | (0.0) | 2 | (10.5) | |  |
| **Prepregnancy mass index** |  |  |  |  |  |  | |  |
| (kg/m^2^; median, Q_1_-Q_3_) | 24.0 | (21.3 - 30.8) | 25.0 | (20.8 - 31.2) | 24.0 | (21.7 - 29.9) | | 0.751 |
| < 25 | 21 | (52.5) | 11 | (50.0) | 10 | (55.6) | | 0.836 |
| 25-29.9 | 8 | (20.0) | 4 | (18.2) | 4 | (22.2) | |  |
| ≥ 30 | 11 | (27.5) | 7 | (31.8) | 4 | (22.2) | |  |
| **Smoking during pregnancy** |  |  |  |  |  |  | |  |
| No | 37 | (88.1) | 19 | (82.6) | 18 | (94.7) | | 0.356 |
| Yes | 5 | (11.9) | 4 | (17.4) | 1 | (5.3) | |  |
| ***Child characteristics at birth*** |  |  |  |  |  |  | |  |
| **Gender *** |  |  |  |  |  |  | |  |
| Male | 28 | (66.7) | 15 | (65.2) | 13 | (68.4) | | 0.826 |
| Female | 14 | (33.3) | 8 | (34.8) | 6 | (31.6) | |  |
| **Gestational age ***(weeks; median, Q_1_-Q_3_) | 38 | (37 - 39) | 38 | (38 - 40) | 38 | (37 - 39) | | 0.687 |
| **Preterm birth** |  |  |  |  |  |  | |  |
| No | 36 | (85.7) | 20 | (87.0) | 16 | (84.2) | | 1.000 |
| Yes | 6 | (14.3) | 3 | (13.0) | 3 | (15.8) | |  |
| **Birthweight *** (kg; median, Q_1_-Q_3_) | 3.15 | (2.89 - 3.40) | 3.19 | (3.03 - 3.46) | 3.10 | (2.62 - 3.34) | | 0.535 |
| **Low birthweight** |  |  |  |  |  |  | |  |
| No | 38 | (90.5) | 22 | (95.7) | 16 | (84.2) | | 0.313 |
| Yes | 4 | (9.5) | 1 | (4.3) | 3 | (15.8) | |  |
| **Height at birth** (cm; median, Q_1-_Q_3_) | 49.5 | (48 - 50) | 50 | (49 - 51) | 48 | (46 - 50) | | 0.063 |
| **Small for gestational age** |  |  |  |  |  |  | |  |
| No | 35 | (83.3) | 20 | (87.0) | 15 | (79.0) | | 0.379 |
| 10^th^-3^rd^ centile | 5 | (11.9) | 3 | (13.0) | 2 | (10.5) | |  |
| < 3^rd^ centile | 2 | (4.8) | 0 | (0.0) | 2 | (10.5) | |  |
| **Head circumference** (cm; median, Q_1_-Q_3_) | 34 | (33 - 35) | 34 | (33 - 35) | 34 | (32 - 35) | | 0.645 |
| **Head circumference z-score** (means, 95% CI) | -.218 | (-.461 - .025) | -.173 | (-.481 - .135) | -.272 | (-.691 - .147) | | 0.876 |
| **Head circumference categories** |  |  |  |  |  |  | |  |
| Normal head, -1 SD ≤ to ≤ +2 SD | 35 | (83.3) | 21 | (91.3) | 14 | (73.7) | | 0.214 |
| Small head, -2 SD ≤ to < - 1 SD | 7 | (16.7) | 2 | (8.7) | 5 | (26.3) | |  |
| Microcephaly, < - 2 SD | 0 | (0.0) | 0 | (0.0) | 0 | (0.0) | |  |
| **Apgar score at 1 minute** |  |  |  |  |  |  | |  |
| 10 | .32 | (76.2) | 17 | (73.9) | 15 | (78.9) | | 0.450 |
| 7-9 | 5 | (11.9) | 4 | (17.4) | 1 | (5.3) | |  |
| < 7 | 5 | (11.9) | 2 | (8.7) | 3 | (15.8) | |  |
| **Breastfeeding** |  |  |  |  |  |  | |  |
| No | 16 | (38.1) | 6 | (26.1) | 10 | (52.6) | | 0.078 |
| Yes | 26 | (61.9) | 17 | (73.9) | 9 | (47.4) | |  |
| ***Characteristics of exposure*** |  |  |  |  |  |  | |  |
| **Month of birth over the epidemic** | |  |  |  |  |  | |  |
| (median, Q_1_-Q_3_) | 12 | (11 - 13) | 12 | (11 - 13) | 11 | (9 - 12) | | **0.023** |
| **Epidemic waves** |  |  |  |  |  |  | |  |
| 1 (March to August 2005) | 4 | (9.5) | 1 | (4.3) | 3 | (15.8) | | 0.313 |
| 2 (September 2005 to August 2006) | 38 | (90.5) | 22 | (95.7) | 16 | (84.2) | |  |
| ***Child characteristics at follow-up*** | |  |  |  |  |  | |  |
| **Age** (years ; medians, Q_1_-Q_3_) | 14.4 | (14.1 - 14.5) | 14.2 | (13.9 - 14.4) | 14.5 | (14.4 - 14.8) | | **< 0.001** |
| 13 to 14 | 9 | (21.4) | 8 | (34.8) | 1 | (5.3) | | **0.027** |
| 14 to 15 | 33 | (78.6) | 15 | (65.2) | 18 | (94.7) | |  |
| **Height** (cm, medians, Q_1_-Q_3_) | 166 | (159 - 175) | 170 | (160 - 176) | 162 | (157 - 172) | | 0.337 |
| **Stunted** (inappropriate height/age) |  |  |  |  |  |  | |  |
| No | 37 | (88.1) | 22 | (95.6) | 15 | (79.0) | | 0.158 |
| Yes | 5 | (11.9) | 1 | (4.4) | 4 | (21.0) | |  |
| **Weight** (kg; median, Q_1_-Q_3_) | 57.2 | (49 - 68) | 56.5 | (51 - 68) | 60 | (48 - 70.7) | | 0.535 |
| **Wasted** (inappropriate weight/height) | |  |  |  |  |  | |  |
| No | 35 | (83.3) | 21 | (91.3) | 14 | (73.7) | | 0.214 |
| Yes | 7 | (16.7) | 2 | (8.7) | 5 | (26.3) | |  |
| **Obese** |  |  |  |  |  |  | |  |
| No | 33 | (78.6) | 21 | (91.3) | 12 | (63.2) | | 0.055 |
| Yes | 9 | (21.4) | 2 | (8.7) | 7 | (36.8) | |  |
| **Head circumference** (cm ; median, Q_1_-Q_3_) | 54.7 | (53 - 56) | 56 | (54 - 57) | 53 | (52 - 54.4) | | **0.002** |
| **Head circumference z-score** (means, 95% CI) | -.000 | (-.311 - .311) | .495 | (.152 - .837) | -.599 | (-1.035 - -.162) | | **<0.001** |
| **Head circumference categories** |  |  |  |  |  |  | |  |
| Large head > + 2 SD | 6 | (14.3) | 5 | (21.7) | 1 | (5.3) | | **<0.001** |
| Normal head, -1 SD ≤ to ≤ +2 SD | 22 | (52.4) | 17 | (73.9) | 5 | (26.3) | |  |
| Small head, -2 SD ≤ to < - 1 SD | 12 | (31.6) | 1 | (4.4) | 11 | (57.9) | |  |
| Microcephaly, < - 2 SD | 2 | (5.2) | 0 | (0.0) | 2 | (10.5) | |  |
| Data are numbers and column percentages, or medians and interquartile ranges (Q1-Q3) when specified. Percentages are compared using Chi2 or Fisher’s Exact test, as appropriate. Medians are compared using a non-parametric Brown-Mood test. | | | | | | | | |

| **Supplementary table 4. Neurodevelopmental average scores of 19 infected children and 23 nonmatched uninfected children exposed to perinatal MTCT of chikungunya, CHIK13+ cohort, Reunion island, 2020-2021.** | | | | | | | |
| --- | --- | --- | --- | --- | --- | --- | --- |
| **Exposure group** | **Total** | | **CHIK -** | | **CHIK +** | |  |
|  | n = 42 | | n = 23 | | n = 19 | |  |
| ***Subtest averages*** | mean | (95% CI) | mean | (95% CI) | mean | (95% CI) | *p value* |
| 1. **WISC-V (Wechsler Intelligence Scale-5th ed) scores** | | | | | | | |
| **Full Scale Intelligence Quotient** | 87.2 | (82.4 - 92.0) | 91.5 | (86.7 - 96.2) | 82.1 | (73.1 - 90.9) | 0.119 |
| Verbal comprehension | 89.6 | (84.0 - 95.3) | 95.7 | (89.5 - 101.7) | 81.9 | (72.2 - 91.6) | **0.030** |
| Visual spatial | 92.3 | (87.7 - 96.8) | 95.7 | (90.8 - 100.5) | 88.1 | (79.9 - 96.3) | 0.210 |
| Fluid reasoning | 90.5 | (85.8 - 95.1) | 93.1 | (88.3 - 97.8) | 87.4 | (78.4 - 96.3) | 0.417 |
| Working memory | 87.2 | (82.8 - 91.5) | 89.3 | (84.5 - 93.8) | 84.7 | (76.6 - 92.9) | 0.568 |
| Processing speed | 93.2 | (88.0 - 98.2) | 99.3 | (94.8 - 103.6) | 85.7 | (76.4 - 95.0) | **0.015** |
| 1. **VABS-II (Vineland Adaptive Behavior Scale-2d ed) scores** | | | | | | | |
| **Adaptive behavior composite** | 93.8 | (87.7 - 99.8) | 100.5 | (96.0 - 104.9) | 85.6 | (73.9 - 97.4) | **0.012** |
| Communication | 83.9 | (77.4 - 90.3) | 91.0 | (84.3 - 97.6) | 75.3 | (64.0 - 86.5) | **0.032** |
| Daily living skills | 106.2 | (99.3 - 113.1) | 113.6 | (109.9 - 117.3) | 97.3 | (83.0 - 111.6) | 0.315 |
| Socialization | 91.2 | (83.8 - 98.6) | 97.0 | (87.1 - 106.8) | 84.2 | (72.8 - 95.6) | **0.006** |
| 1. **Parent-reported SDQ (Strength and Difficulties Questionnaire) scores** | | | | | | | |
| **Total difficulties score** | 9.9 | (8.1 - 11.6) | 9.1 | (6.4 - 11.8) | 10.8 | (8.7 - 12.9) | 0.209 |
| Emotional symptoms | 2.6 | (1.9 - 3.3) | 2.8 | (1.8 - 3.8) | 2.3 | (1.2 - 3.3) | 0.358 |
| Conduct problems | 1.7 | (1.3 - 2.2) | 1.7 | (1.1 - 2.3) | 1.7 | (1.0 - 2.4) | 0.931 |
| Hyperactivity / inattention | 3.3 | (2.4 - 4.0) | 2.5 | (1.5 - 3.4) | 4.2 | (2.9 - 5.5) | **0.028** |
| Peer relationships problems | 2.7 | (2.0 - 3.3) | 2.7 | (1.5 - 3.8) | 2.6 | (2.0 - 3.1) | 0.439 |
| Prosocial behavior | 8.0 | (7.3 - 8.7) | 8.3 | (7.3 - 9.2) | 7.7 | (6.7 - 8.7) | 0.276 |
| MTCT: mother-to-child transmission. Data are unpaired means and 95% confidence intervals. Unpaired means are compared using a Mann-Whitney Wilcoxon test. | | | | | | | |

| \| \| **Supplementary table 5. Cognitive, adaptative and behavioural skills of 19 infected children and 23 nonmatched uninfected children exposed to perinatal MTCT of chikungunya, CHIK13+ cohort, Reunion island, 2020-2021** \| \| --- \| \| \| --- \| --- \| | | | | | | | | | |
| --- | --- | --- | --- | --- | --- | --- | --- | --- | --- | --- | --- |
| **Exposure group** | **Total** | | | **CHIK -** | | | **CHIK +** | |  |
|  | n = 42 | | | n = 23 | | | n = 19 | |  |
| **1. Neurocognitive deficits (WISC-V)** | n | (%) | n | | (%) | | n | (%) | *p value* |
| **Full Scale Intelligence Quotient** |  |  |  | |  | |  |  | b+c vs a : |
| Age-appropriate skill (a) | 22 | (52.4) | 14 | | (60.9) | | 8 | (42.1) | 0.226 |
| Mild to moderate deficit (b) | 15 | (35.7) | 9 | | (39.1) | | 6 | (31.6) | c vs a+b : |
| Severe deficit//intellectual disability (c) | 5 | (11.9) | 0 | | (0.0) | | 5 | (26.3) | **0.014** |
| **Verbal comprehension** |  |  |  | |  | |  |  | b+c vs a : |
| Age-appropriate skill (a) | 23 | (56.1) | 16 | | (69.6) | | 7 | (38.9) | 0.050 |
| Mild to moderate deficit (b) | 14 | (34.1) | 7 | | (30.4) | | 7 | (38.9) | c vs a+b : |
| Severe deficit (c) | 4 | (9.8) | 0 | | (0.0) | | 4 | (22.2) | **0.030** |
| **Visual spatial** |  |  |  | |  | |  |  | b+c vs a : |
| Age-appropriate skill (a) | 31 | (73.8) | 19 | | (82.6) | | 12 | (63.2) | 0.180 |
| Mild to moderate deficit (b) | 8 | (19.1) | 4 | | (17.4) | | 4 | (21.0) | c vs a+b : |
| Severe deficit (c) | 3 | (7.1) | 0 | | (0.0) | | 3 | (15.8) | 0.084 |
| **Fluid reasoning** |  |  |  | |  | |  |  | b+c vs a : |
| Age-appropriate skill (a) | 28 | (66.7) | 17 | | (73.9) | | 11 | (57.9) | 0.273 |
| Mild to moderate deficit (b) | 10 | (23.8) | 6 | | (26.1) | | 4 | (21.0) | c vs a+b : |
| Severe deficit (c) | 4 | (9.5) | 0 | | (0.0) | | 4 | (21.0) | **0.035** |
| **Working memory** |  |  |  | |  | |  |  | b+c vs a : |
| Age-appropriate skill (a) | 24 | (57.1) | 14 | | (60.9) | | 10 | (52.6) | 0.591 |
| Mild to moderate deficit (b) | 14 | (33.3) | 9 | | (39.1) | | 5 | (26.3) | c vs a+b : |
| Severe deficit (c) | 4 | (9.5) | 0 | | (0.0) | | 4 | (21.0) | **0.035** |
| **Processing speed** |  |  |  | |  | |  |  | b+c vs a : |
| Age-appropriate skill (a) | 32 | (76.2) | 22 | | (95.6) | | 10 | (52.6) | **0.002** |
| Mild to moderate deficit (b) | 7 | (16.7) | 1 | | (4.4) | | 6 | (31.6) | c vs a+b : |
| Severe deficit (c) | 3 | (7.1) | 0 | | (0.0) | | 3 | (15.8) | 0.084 |
| **2. Delayed adaptive skills (VABS-II)** |  |  |  | |  | |  |  |  |
| **Adaptive behavior composite** |  |  |  | |  | |  |  | b+c vs a : |
| Normal adaptive (a) | 35 | (83.3) | 21 | | (91.3) | | 14 | (73.7) | 0.214 |
| Low adaptive (b) | 3 | (7.1) | 2 | | (8.7) | | 1 | (5.3) | c vs a+b : |
| Very low adaptive (c) | 4 | (9.5) | 0 | | (0.0) | | 4 | (21.0) | **0.035** |
| **Communication** |  |  |  | |  | |  |  | b+c vs a : |
| Normal adaptive (a) | 27 | (64.3) | 16 | | (69.6) | | 11 | (57.9) | 0.432 |
| Low adaptive (b) | 9 | (21.4) | 5 | | (21.7) | | 4 | (21.0) | c vs a+b : |
| Very low adaptive (c) | 6 | (14.3) | 2 | | (8.7) | | 4 | (21.0) | 0.384 |
| **Daily living skills** |  |  |  | |  | |  |  | b+c vs a : |
| Normal adaptive (a) | 35 | (83.3) | 21 | | (91.3) | | 14 | (73.7) | **0.014** |
| Low adaptive (b) | 2 | (4.8) | 1 | | (4.3) | | 1 | (5.3) | c vs a+b : |
| Very low adaptive (c) | 5 | (11.9) | 1 | | (4.3) | | 4 | (21.0) | **0.035** |
| **Socialization** |  |  |  | |  | |  |  | b+c vs a : |
| Normal adaptive (a) | 32 | (84.2) | 18 | | (94.7) | | 14 | (73.7) | 0.214 |
| Low adaptive (b) | 8 | (21.1) | 1 | | (5.3) | | 1 | (5.3) | c vs a+b : |
| Very low adaptive (c) | 4 | (10.5) | 0 | | (0.0) | | 4 | (21.0) | 0.158 |
| *To be continued* |  |  |  | |  | |  |  |  |
| **3. Parent-reported SDQ** |  |  | |  | |  |  |  |  |
| **Total difficulties score** |  |  |  | |  | |  |  | b+c vs a : |
| Absent (a) | 30 | (75.0) | 17 | | (77.3) | | 13 | (72.2) | 0.731 |
| Mild to moderate (b) | 4 | (10.0) | 2 | | (9.1) | | 2 | (11.1) | c vs a+b : |
| High (c) | 6 | (15.0) | 3 | | (13.6) | | 3 | (16.7) | 1.000 |
| **Emotional symptoms** |  |  |  | |  | |  |  | b+c vs a : |
| Absent (a) | 24 | (64.9) | 14 | | (63.6) | | 10 | (66.7) | 0.850 |
| Mild to moderate (b) | 5 | (13.5) | 2 | | (9.1) | | 3 | (20.0) | c vs a+b : |
| High (c) | 8 | (21.6) | 6 | | (27.3) | | 2 | (13.3) | 0.431 |
| **Conduct problems** |  |  |  | |  | |  |  | b+c vs a : |
| Absent (a) | 30 | (75.0) | 18 | | (81.8) | | 12 | (66.7) | 0.300 |
| Mild to moderate (b) | 7 | (17.5) | 3 | | (13.6) | | 4 | (22.2) | c vs a+b : |
| High (c) | 3 | (7.5) | 1 | | (4.6) | | 2 | (11.1) | 0.579 |
| **Hyperactivity / inattention** |  |  |  | |  | |  |  | b+c vs a : |
| Absent (a) | 31 | (77.5) | 19 | | (86.4) | | 12 | (73.7) | 0.253 |
| Mild to moderate (b) | 4 | (10.0) | 2 | | (9.1) | | 2 | (11.1) | c vs a+b : |
| High (c) | 5 | (12.5) | 1 | | (4.5) | | 4 | (22.2) | 0.155 |
| **Peer relationships problems** |  |  |  | |  | |  |  | b+c vs a : |
| Absent (a) | 18 | (45.0) | 11 | | (50.0) | | 7 | (38.9) | 0.482 |
| Mild to moderate (b) | 15 | (37.5) | 7 | | (31.8) | | 8 | (44.4) | c vs a+b : |
| High (c) | 7 | (17.5) | 4 | | (18.2) | | 3 | (16.7) | 1.000 |
| **Prosocial behavior** |  |  |  | |  | |  |  | b+c vs a : |
| Normal (a) | 30 | (75.0) | 18 | | (81.8) | | 12 | (66.6) | 0.300 |
| Intermediate (b) | 7 | (17.5) | 2 | | (9.1) | | 5 | (27.8) | c vs a+b : |
| Abnormal (c) | 3 | (7.5) | 2 | | (9.1) | | 1 | (5.6) | 1.000 |
| WISC-V: Wechsler Intelligence Scale-5th ed scale. VABS-II: Vineland Adaptive Behavior Scale-2d ed scale. SDQ: Strength and Difficulties Questionnaire. MTCT: mother-to-child transmission. Cognitive functions, adaptive skills and behavioural skills are categorized as follows: (a) Normal: score >-1 SD; (b) Low, mildly or moderately impaired: < -2 SD score ≤ -1 SD; (c) Very low, severely or highly impaired: score ≤ -2 SD. Percentages are compared using Chi2 or Fisher’s Exact tests, as appropriate. | | | | | | | | | |

| \| **Supplementary Table 6. Subsidiary outcomes and health issues for the 42 children (matched or not) exposed to perinatal mother-to-child transmission of chikungunya, CHIK13+, Reunion island, 2020-2021.** \| \| --- \| | | | | | | | |
| --- | --- | --- | --- | --- | --- | --- | --- | --- |
| **Exposure group** | **Total** | | **CHIK -** | | **CHIK +** | |  |
|  | n = 42 | | n = 23 | | n = 19 | | *p value* |
| ***Development milestones*** |  |  |  |  |  |  |  |
| **Delay in walking alone without help** |  |  |  |  |  |  |  |
| No | 36 | (85.7) | 23 | (100) | 13 | (68.4) | *****0.005** |
| Yes | 6 | (14.3) | 0 | (0.0) | 6 | (31.6) |  |
| **Delay in language acquisition** |  |  |  |  |  |  |  |
| No | 34 | (81.0) | 23 | (100) | 11 | (57.9) | *****0.001** |
| Yes | 8 | (19.0) | 0 | (0.0) | 8 | (42.1) |  |
| ***Sensorineural outcomes*** |  |  |  |  |  |  |  |
| **Strabismus** |  |  |  |  |  |  |  |
| No | 34 | (81.0) | 22 | (95.6) | 12 | (63.2) | ****0.015** |
| Yes | 8 | (19.0) | 1 | (4.4) | 7 | (36.8) |  |
| **Worst -eye mean ocular deficit** (diopters, µ ± SD) | 8.5 | (2.7) | 9.0 | (2.4) | 8.0 | (3.0) | **0.041** |
| **Loss of visual acuity** |  |  |  |  |  |  |  |
| No | 26 | (61.9) | 18 | (78.3) | 8 | (42.1) | **0.016** |
| Yes | 16 | (38.1) | 5 | (21.7) | 11 | (57.9) |  |
| **Wearing correction lenses** |  |  |  |  |  |  |  |
| No | 26 | (61.9) | 17 | (73.9) | 9 | (47.4) | 0.078 |
| Yes | 16 | (38.1) | 6 | (26.1) | 10 | (52.6) |  |
| **Hearing difficulties** |  |  |  |  |  |  |  |
| No | 39 | (92.9) | 23 | (100) | 16 | (84.2) | 0.084 |
| Yes | 3 | (7.1) | 0 | (0.0) | 3 | (15.8) |  |
| **Hearing aid with apparatus** |  |  |  |  |  |  |  |
| No | 42 | (100) | 23 | (100) | 19 | (100) | 1.000 |
| Yes | 0 | (0.0) | 0 | (0.0) | 0 | (0.0) |  |
| ***Special care and interdisciplinary follow-up*** |  |  |  |  |  |  |  |
| **Physiotherapist** |  |  |  |  |  |  |  |
| No | 35 | (83.3) | 23 | (100) | 12 | (63.2) | *****0.002** |
| Yes | 7 | (16.7) | 0 | (0.0) | 7 | (36.8) |  |
| **Speech therapist** |  |  |  |  |  |  |  |
| No | 30 | (71.4) | 19 | (82.6) | 11 | (57.9) | 0.098 |
| Yes | 12 | (28.6) | 4 | (17.4) | 8 | (42.1) |  |
| **Psychomotor therapist** |  |  |  |  |  |  |  |
| No | 36 | (85.7) | 23 | (100) | 13 | (68.4) | *****0.005** |
| Yes | 6 | (14.3) | 0 | (0.0) | 6 | (31.6) |  |
| **Early neuropsychological follow-up (CAMSP)** |  |  |  |  |  |  |  |
| No | 32 | (76.2) | 23 | (100) | 9 | (57.9) | ****<0.001** |
| Yes | 10 | (23.8) | 0 | (0.0) | 10 | (42.1) |  |
| **Ongoing rehabilitative support (CMPP)** |  |  |  |  |  |  |  |
| No | 38 | (90.5) | 23 | (100) | 15 | (78.9) | *****0.035** |
| Yes | 4 | (9.5) | 0 | (0.0) | 4 | (21.1) |  |
| ***Schooling and learning*** |  |  |  |  |  |  |  |
| **School class on assessment day** |  |  |  |  |  |  |  |
| Mainstream class without support | 38 | (90.5) | 23 | (100) | 15 | (78.9) | **0.035** |
| Mainstream class with *ULIS* support | 3 | (7.1) | 0 | (0.0) | 4 | (15.8) |  |
| Special education and home care service | 1 | (2.4) | 0 | (0.0) | 1 | (5.3) |  |
| **Average deviation from expected class** (years, µ ± SD) | -0.6 | (2.0) | 0.1 | (0.3) | -1.4 | (2.7) | **0.006** |
| **RRepeated grades at school** |  |  |  |  |  |  |  |
| No | 38 | (90.5) | 23 | (100) | 15 | (78.9) | *****0.035** |
| Yes | 4 | (9.5) | 0 | (0.0) | 4 | (21.1) |  |
| ***Health issues*** |  |  |  |  |  |  |  |
| **Cerebral palsy** |  |  |  |  |  |  |  |
| No | 40 | (71.4) | 23 | (82.6) | 17 | (57.9) | ***0.199 |
| Yes | 2 | (28.6) | 0 | (17.4) | 2 | (42.1) |  |
| **Swallowing disorders** |  |  |  |  |  |  |  |
| No | 41 | (97.6) | 23 | (82.6) | 18 | (94.7) | ***0.452 |
| Yes | 1 | (2.4) | 0 | (17.4) | 1 | (2.3) |  |
| **Sleep disorders** |  |  |  |  |  |  |  |
| No | 39 | (92.9) | 23 | (100) | 16 | (84.2) | ***0.084 |
| Yes | 3 | (7.1) | 0 | (0.0) | 3 | (15.8) |  |
| Data are numbers and column percentages or means and standard deviations when specified. Percentages are compared using chi2 or Fisher’s Exact test, as appropriate and means are compared using a non-parametric Mann-Whitney-Wilcoxon test in full cohort analysis: * p<0.05; ** p<0.01; ***p<0.001. | | | | | | | |

**Supporting file 2. Supplementary Table 7: Score correlations appendix**

**A. Subscale 2*2 correlations in the infected children group (Spearmon Rho test)**

Spearman Rho coefficient values are displayed on the left and down to the dark diagonal boxes.

*P* values are displayed on the right and up to the dark diagonal boxes.

**B. Subscale 2*2 correlations in the uninfected children group (Spearmon Rho test)**

Spearman Rho coefficient values are displayed on the left and down to the dark diagonal boxes.

*P* values are displayed on the right and up to the dark diagonal boxes.

**C. Subscale 2*2 correlations in the full adolescent child cohort (Spearmon Rho test)**

Spearman Rho coefficient values are displayed on the left and down to the dark diagonal boxes.

*P* values are displayed on the right and up to the dark diagonal boxes.

**Supporting file 3. Supplementary Table 8: Longitudinal analysis of infected children**

**A. Longitudinal analysis of score rank-ordered distributions, correlations and inter-agreement between CHIMERE scores (2 years of age) and CHIK13+ scores (14-15 years) in 17 infected children**

*Correlations between scores are estimated using Spearman Rho (ρ) coefficient.

**Scores are compared as paired repeated measures using Wilcoxon signed-rank test (z).

Inter-agreement between score categories is assessed using Cohen's kappa (κ).

Data are *p* values. An early test is considered predictable if ρ and κ are significant and z is nonsignificant.

**B. Longitudinal analysis of score rank-ordered performances, correlations and inter-agreement between CHIMERE scores (2 years of age) and CHIK13+ scores (14-15 years) in 12 nonencephalitic children**

*Correlations between scores are estimated using Spearman Rho (ρ) coefficient.

**Scores are compared as paired repeated measures using Wilcoxon signed-rank test (z).

Inter-agreement between score categories is assessed using Cohen's kappa (κ).

Data are *p* values. An early test is considered predictable if ρ and κ are significant and z is nonsignificant.

**C. Longitudinal analysis of score rank-ordered performances, correlations and inter-agreement between CHIMERE scores (2 years of age) and CHIK13+ scores (14-15 years) in 6 encephalitic children**

| *Correlations between scores are estimated using Spearman Rho (ρ) coefficient.  **Scores are compared as paired repeated measures using Wilcoxon signed-rank test (z).  Inter-agreement between score categories is assessed using Cohen's kappa (κ).  Data are *p* values. An early test is considered predictable if ρ and κ are significant and z is nonsignificant. |
| --- |
