# Supplementary material for: Long-term neurodevelopmental outcomes of children perinatally infected with chikungunya: the CHIK13+ matched cohort study on Reunion Island
Source: eClinicalMedicine. 2026 May 18;95:103975. doi: 10.1016/j.eclinm.2026.103975 (PMC13208087; doi:10.1016/j.eclinm.2026.103975)
Supplement: Protocole [file mmc4.pdf]

Conséquences d'une infection materno-foetale au virus du *chikungunya*. Evaluation neurocognitive et sensorielle vers l'âge de 13 ans

CHIK 13+

2018/CHU/07

PROTOCOLE DE RECHERCHE INTERVENTIONNELLE

IMPLIQUANT LA PERSONNE HUMAINE

(Catégorie 2 risques et contraintes minimes)

Version n°3.0 du 10/10/2019
N° IDRCB : 2019-A02095-52

Promoteur :

Centre Hospitalier Universitaire (CHU) de la Réunion
Site du Groupe Hospitalier Sud Réunion (GHSR)
97448 Saint Pierre – Cedex – La Réunion

Investigateur principal:

Docteur Raphaëlle SARTON
Fonction : assistante des hôpitaux de la Réunion
Service de Pédiatrie générale, CHU Réunion, site du GHSR, Saint Pierre – La Réunion
Adresse électronique : sartonraphaelle@gmail.com

Projet sous la supervision du :
Docteur Brahim BOUMAHNI
Fonction : Praticien Hospitalier
Service de Néonotalogie, Réanimation Pédiatrique et Néonatale, CHU Réunion, site du GHSR, Saint Pierre – La Réunion
Adresse électronique : brahim.boumahni@chu-reunion.fr

Centre de Méthodologie et de Gestion des données :

Docteur Patrick GERARDIN
Fonction : Praticien Hospitalier
Centre d'Investigation Clinique Epidémiologie Clinique de la Réunion
INSERM CIC 1410 – CHU Réunion, site du GHSR, Saint Pierre – La Réunion

Ce protocole a été conçu et rédigé à partir de la version 3.0 du 01/02/2017 du protocole-type du GIRCI SOHO

HISTORIQUE DES MISES A JOUR DU PROTOCOLE

VERSION	DATE	RAISON DE LA MISE A JOUR
1.0	26/09/2018	Soumission à l'AOI médical 2018 du CHU de La Réunion
2.0	04/07/2019	Soumission initiale CPP.
3.0	10/10/2019	Réponses aux demandes d'informations complémentaires émises par le CPP

PAGE D'APPROBATION DU PROTOCOLE

Conséquences d'une infection materno-foetale au virus du *chikungunya*. Evaluation neurocognitive et sensorielle vers l'âge de 13 ans

CHIK 13+

PROTOCOLE DE RECHERCHE INTERVENTIONNELLE IMPLIQUANT LA PERSONNE HUMAINE *(risques et contraintes minimes)*

COMITE DE REDACTION	Raphaëlle SARTON<br>Brahim BOUMAHNI<br>Patrick GERARDIN<br>Samir MEDJANE

NOMS ET TITRES DES RESPONSABLES	COORDONNÉES	DATE (jj-mm-aa)	SIGNATURE
Investigateur Principal pour le CHU	Dr Raphaëlle SARTON<br>Pédiatre Générale<br>CHU Réunion, GHSR, St Pierre		
Président de la Délégation à la Recherche Clinique et à l'Innovation	Dr Silvia IACOBELLI<br>DRCI de la Réunion		
Structure Méthodologique	Dr Catherine MARIMOUTOU<br>INSERM CIC 1410 –<br>Epidémiologie Clinique		
Chef de Projet	M. Samir MEDJANE<br>DRCI de la Réunion		

PAGE DE SIGNATURE DU PROTOCOLE

Conséquences d'une infection materno-foetale au virus du *chikungunya*. Evaluation neurocognitive et sensorielle vers l'âge de 13 ans.

CHIK 13+

Promoteur
Centre Hospitalier Universitaire de la Réunion
Groupe Hospitalier Sud Réunion – BP 350
97448 Saint Pierre Cedex – La Réunion
Tel : 02 62 35 77 87
Fax : 02 62 35 97 21
Courriel : manuella.pothin@chu-reunion.fr

à Saint Pierre, le

Manuella POTHIN
Signature

Investigateur principal
Dr Raphaëlle SARTON
Service de Pédiatrie générale,
CHU Réunion, site du GHSR,
Saint Pierre – La Réunion
Tel : 06.93.82.84.30
Courriel : sartonraphaelle@gmail.com

à Saint Pierre, le :

Dr Raphaelle SARTON
Signature

PRINCIPAUX CORRESPONDANTS

Investigateur principal
Dr Raphaëlle SARTON
Service de Pédiatrie générale,
CHU Réunion, site du GHSR,
Saint Pierre – La Réunion
Tel : *06.93.82.84.30*
Courriel : *sartonraphaelle@gmail.com*

Co-Investigateur
Dr Brahim BOUMAHNI
Service de Néonotalogie, Réanimation Pédiatrique et Néonatale, CHU Réunion, site du GHSR, Saint Pierre – La Réunion
Tel : 02.62.35.91.49
Courriel : *brahim.boumahni@chu-reunion.fr*

Autres spécialités
Mme Marie Odile MERY
Neuropsychologue
Centre d'Action Médico-Sociale Précoce
Fondation du Père Favron
97450 Saint Louis - La Réunion
Tel : 02.62.91.82.32
Courriel : *mo_mery@hotmail.com*

Promoteur
Mme Manuella POTHIN
à Saint Pierre, le *: date*
Centre Hospitalier Universitaire de la Réunion
Groupe Hospitalier Sud Réunion –
BP 350 97448 Saint Pierre Cedex –
La Réunion
Tel : 02 62 35 77 87 - Fax : 02 62 35 97 21
Courriel : *manuella.pothin@chu-reunion.fr*

Centre de Méthodologie et de Gestion des données
à Saint Pierre, le : date
Dr Patrick GERARDIN
INSERM CIC 1410 Epidémiologie Clinique
site du GHSR, Saint Pierre – La Réunion
Tel : 02.62.35.90.00 + poste 5.87.53
Courriel : *patrick.gerardin@chu-reunion.fr*

SOMMAIRE

PAGE D'APPROBATION DU PROTOCOLE 3
Page de signature du protocole 4
SOMMAIRE 6
1. RESUME DE LA RECHERCHE 9
2. JUSTIFICATION SCIENTIFIQUE ET DESCRIPTION GENERALE 13
2.1. ETAT ACTUEL DES CONNAISSANCES SUR LA PATHOLOGIE 13
2.2. HYPOTHESES DE LA RECHERCHE ET RESULTATS ATTENDUS 15
2.3. JUSTIFICATION DES CHOIX METHODOLOGIQUES 16
2.3.1. JUSTIFICATION DU SCHEMA DE LA RECHERCHE 16
2.3.2 JUSTIFICATION DU CRITERE DE JUGEMENT PRINCIPAL 17
2.3.3 JUSTIFICATION DU TYPE DE COMPARAISON 17
2.3.4. JUSTIFICATION DE LA CONDUITE DE LA RECHERCHE 17
2.4. RAPPORT BENEFICE / RISQUE 17
2.5. RETOMBEES ATTENDUES 18
2.6. JUSTIFICATION DU FAIBLE NIVEAU D'INTERVENTION 19
3. OBJECTIFS DE LA RECHERCHE 20
3.1 OBJECTIF PRINCIPAL 20
3.2. OBJECTIFS SECONDAIRES 20
4. CRITERES DE JUGEMENT 21
4.1. CRITERE DE JUGEMENT PRINCIPAL 21
4.2. CRITERES DE JUGEMENT SECONDAIRES 21
5. CONCEPTION DE LA RECHERCHE 23
5.1. SCHEMA DE LA RECHERCHE 23
5.2. METHODES POUR L'APPARIEMENT DES NON-EXPOSES 23
6. CRITERES D'ELIGIBILITE 25
6.1. CRITERES D'INCLUSION 25
6.2. CRITERES DE NON-INCLUSION 25
6.3. FAISABILITE ET MODALITES DE RECRUTEMENT 27
7. TRAITEMENT(S)/STRATEGIE(S)/PROCEDURE(S) DE LA RECHERCHE 28
7.1. TRAITEMENT/STRATEGIE/PROCEDURE EXPERIMENTAL(E) 28
7.2. TRAITEMENT/STRATEGIE/PROCEDURE DE COMPARAISON 28
8. DEROULEMENT DE LA RECHERCHE 31
8.1. CALENDRIER DE LA RECHERCHE 31
8.2. VISITE D'INCLUSION V1 (M0) 32
8.2.1. Recueil du consentement 32
8.3.2 Déroulement de la visite 32
8.3. VISITES DE SUIVI V2 (M3 (+/- 1MOIS)) 32
8.1. VISITE DE SUIVI V3 (M6 +/-3MOIS) 33
8.6 REGLES D'ARRET DE LA PARTICIPATIONS D'UNE PERSONNE A LA RECHERCHE 33
8.7 CONTRAINTES LIEES A LA RECHERCHE ET INDEMNISATION EVENTUELLE DES PARTICIPANTS 33

9. GESTION DES ÉVÉNEMENTS INDÉSIRABLES ET DES FAITS NOUVEAUX **34**

10. ASPECTS STATISTIQUES **35**

10.1. CALCUL DE LA TAILLE D'ETUDE 35
0.2. METHODES STATISTIQUES EMPLOYEES 36
10.2.1 PLan d'analyse *36*
10.2.2 CRITERES DE JUGEMENT QUANTITATIFS *36*
10.2.3 CRITERES DE JUGEMENT qualitatifs *36*

11. SURVEILLANCE DE LA RECHERCHE **38**

11.1 CONSEIL SCIENTIFIQUE 38

12. DROITS D'ACCES AUX DONNEES ET DOCUMENTS SOURCE **39**

12.1. ACCES AUX DONNEES 39
12.2. DONNEES SOURCE 39
12.3. CONFIDENTIALITE DES DONNEES 39

13. CONTROLE ET ASSURANCE QUALITE **40**

13.1. CONSIGNES POUR LE RECUEIL DES DONNEES 40
13.2. CONTROLE QUALITE 40
13.3. GESTION DES DONNEES 40
13.4. AUDIT ET INSPECTION 40

14. CONSIDERATIONS ETHIQUES ET REGLEMENTAIRES **41**

15. CONSERVATION DES DOCUMENTS ET DES DONNEES RELATIFS A LA RECHERCHE **43**

16. RAPPORT FINAL **43**

17. REGLES RELATIVES A LA PUBLICATION **44**

17.1. COMMUNICATIONS SCIENTIFIQUES 44
17.2. COMMUNICATION DES RESULTATS AUX PARTICIPANTS 44
17.3. CESSION DES DONNEES 44

ANNEXES **49**

ANNEXE 1A **49**

ANNEXE 1B **52**

LISTE DES ABREVIATIONS

AECD	Association pour l'Education Cognitive et le Développement
AEMO	Action Educative en Milieu Ouvert
ALD	Allocation Longue Durée
ANSM	Agence Nationale de Sécurité du Médicament et des produits de santé
ACSOS	Agression cérébrale secondaire d'origine systémique
CAMSP	Centre d'Action Médico-Sociale Précoce
CHIKV	Chikungunya virus
CHIMERE	Chikungunya Mère-Enfant
CLIS	Classe d'Intégration Scolaire
CMPP	Centre Médico-Psycho-Pédagogique
CMV	Cytomégalovirus
CPP	Comité de Protection des Personnes
EDA	Évaluation des fonctions cognitives et Apprentissages de l'enfant
EIG	Effet Indésirable Grave
EIGI	Effet Indésirable Grave Inattendu
EvI	Evènement Indésirable
EvIG	Evènement Indésirable Grave
FLAIR	Fluid Attenuation Inversion Recovery
ICV	Indice de Compréhension Verbale
IMT	Indice de Mémoire de Travail
IRF	Indice de Raisonnement Fluide
IRM	Imagerie par Résonance Magnétique
IVS	Indice visuo-spatial
IVT	Indice de Vitesse de Traitement
LCR	Liquide céphalorachidien
LPV	Leucomalacie périventriculaire
QD	Quotient de développement
QIT	Quotient intellectuel total
MEOPA	Mélange équimolaire en oxygène et protoxyde d'azote
MFIU	Mort fœtale in utéro
RT-PCR	Reverse transcriptase polymerase chain reaction
SAF	Syndrome d'alcoolisation foetale
SDQ	Strength and difficulties questionnaire
SESSAD	Service d'Education Spécialisée et de Soins A Domicile
SNC	Système nerveux central
VABS-II	Vineland Adaptative Behavior Scale, 2nde version
WISC-5	Wechsler Intelligence Scale for Children de 5ème génération

1. RESUME DE LA RECHERCHE

**PROMOTEUR**	Centre Hospitalier Universitaire de la Réunion<br>Groupe Hospitalier Sud Réunion – BP 350
**INVESTIGATEUR PRINCIPAL**	Dr Raphaëlle SARTON
**TITRE**	Conséquences d'une infection materno-foetale au virus du chikungunya. Evaluation neurocognitive et sensorielle à l'âge de 13 ans.<br>**CHIK 13+**
**JUSTIFICATION / CONTEXTE**	Le chikungunya est une maladie infectieuse due à un alphavirus transmis par les moustiques Aedes qui a connu une expansion mondiale depuis sa réémergence en 2004. A la faveur d'une épidémie sans précédent, les pédiatres réunionnais ont décrit en 2005-2006 une transmission verticale materno-fœtale de ce virus, au moment de l'accouchement. Depuis, ce mode de transmission a été largement confirmé, avec un risque absolu estimé entre 15,5% et 48,3%. Les principales conséquences pour l'enfant sont d'ordre neuromoteur, neurosensoriel ou neurocognitif. Elles ont été étudiées vers l'âge de 2 ans chez 33 enfants dans la cohorte CHIMERE, ainsi qu'à l'âge de 5 ans sur une petite fraction de ces enfants suivis au C.A.M.S.P (Centre d'Action Médico-Sociale Précoce). Les résultats suggéraient un retard global des acquisitions psychomotrices secondaire à l'infection néonatale, portant sur les fonctions de la région préfrontale (en particulier coordination et langage). Les performances étaient corrélées à la gravité de la présentation clinique (plus sévères en cas d'encéphalite ou d'encéphalopathie) tout en restant sub-optimales chez les enfants ayant présenté une infection non compliquée. Au cours du suivi neurodéveloppemental, d'autres traits inquiétants sont venus compléter le spectre des problèmes présentés par ces enfants, telles qu'une microcéphalie, une infirmité motrice cérébrale, une épilepsie, un trouble des interactions ou un déficit de l'attention. Nous avons réévalué, vers l'âge de 10 ans, 21 de ces enfants à l'aide du test de dépistage EDA (Evaluation des fonctions cognitives et Apprentissages de l'enfant). Nous souhaiterions désormais confirmer et caractériser leurs déficiences à l'aide d'une batterie de tests confirmatoires vers l'âge de 13 ans.
**OBJECTIFS**	**Objectif principal** :<br><br>Evaluer à l'aide du Quotient Intellectuel Total (QIT) de l'échelle WISC-5 (Wechsler Intelligence Scale for Children 5th version) vers l'âge de 13 ans la performance neurocognitive d'enfants infectés à la naissance par transmission materno-fœtale du CHIKV en comparaison de celle d'un groupe d'enfants non infectés, sélectionnés dans le relevé épidémiologique périnatal des maternités pour maitriser l'exposition, ceux-ci étant appariés sur les principaux facteurs de confusion.<br><br>**Objectifs secondaires :**

	Evaluer la compréhension verbale, le raisonnement visuo-spatial et le raisonnement fluide, la mémoire de travail et la vitesse de traitement, à l'aide du profil des notes standards et des notes composites des cinq échelles psychométriques ou indices spécifiques (dimensions) composant le WISC-5 (respectivement ICV, IVS, IRF, IMT, IVT)<br><br>Evaluer les fonctions exécutives à l'aide du score total composite et des indices spécifiques des quatre domaines de l'échelle VABS-II (Vineland Adaptive Behavior Scale 2EME version) à savoir : communication, autonomie dans la vie quotidienne, socialisation et motricité.<br><br>Evaluer le comportement et les troubles du comportement à l'aide du questionnaire subsidiaire de l'échelle VABS-II et des questionnaires SDQ (Strength and Difficulties Questionnaire). Le questionnaire des troubles du comportement de l'échelle VABS-II permet un dépistage des troubles autistiques tandis que les questionnaires SDQ permettent d'évaluer l'enfant dans son milieu familial, à l'école ainsi qu'une auto-évaluation de l'enfant par rapport à son environnement.<br><br>Evaluer les capacités et les déficits visuels d'une acuité visuelle et de tests de la motilité oculaire<br><br>Evaluer la scolarité de l'enfant (scolarité adaptée pour l'âge au collège, avec ou sans soutien scolaire, années redoublées ; scolarité en institution ou école spécialisée ; recours au CLIS, SESSAD, etc…)<br><br>Evaluer le recours aux soins spécialisés suivants : orthophonie, orthoptie, psychomotricité, orthopédie, kinésiethérapie et rééducation fonctionnelle
**CRITÈRES DE JUGEMENT**	**Critères de jugement principal :**<br>Quotient intellectuel total (QIT) de l'échelle WISC-V (Wechsler Intelligence Scale for Children 5th version).<br><br>**Critères de jugement secondaires :**<br>• Score total composite (moyenne 100 et déviation standard ± 15) de l'échelle VABS-II (Vineland Adaptive Behavior Scale 2nd version) et indices spécifiques de ses quatre domaines (moyenne 15 et déviation standard ± 3) : communication, autonomie pour les gestes de la vie quotidienne, socialisation, motricité.<br><br>• Trouble (oui/non) du comportement identifié par les 27 questions additionnelles du VABS-II<br><br>• Scores de difficultés totales et scores des quatres premiers indices (activité, problèmes de conduite, émotions, problèmes avec les pairs) du SDQ (Strength and Difficulties Questionnaire) validé chez les enfants âgés de 4

	à 16 ans.<br><br>• , troubles de la convergence ou de la motilité oculaire (hétérophorie, voire strabisme) et déficit de l'acuité visuelle (oui/non) et si oui, type de déficit, port de prothèse auditive (oui/non); port de lunettes (oui/non)<br><br>• Type d'école fréquentée en catégoriel (collège normal/institution ou école spécialisée) ; Niveau de scolarité (classe adpatée pour l'âge sans soutien scolaire/classe adaptée pour l'âge avec soutien scolaire/retard scolaire avec redoublement)<br><br>• Recours à des soins spécialisés : orthophoniste (oui/non) ; orthoptiste (oui/non) ; psychomotricien (oui /non); kinésithérapie et rééducation fonctionnelle (oui/non)
**SCHÉMA DE LA RECHERCHE**	Il s'agit d'une étude comparative de cohorte de type exposé - non exposé appariée à visée pronostique, monocentrique.
**CRITERES D'INCLUSION**	- Enfant né entre mars 2005 et juillet 2006<br>- Dont la mère identifiée dans la cohorte CHIMERE ou le registre perinatal des maternités du Sud Réunion<br>- Exposé : enfant infecté par le virus chikungunya au moment de l'accouchement<br>- Non exposé : enfant non infecté par le virus chikungunya au moment de l'accouchement vérifiant les critères d'appariement précisés au chapitre 5.2<br>- Affilié à un régime de sécurité sociale
**CRITERES DE NON INCLUSION**	- Enfant né avant mars 2005 ou après août 2006<br>- Mère non identifiée dans la cohorte CHIMERE ou le registre perinatal de la maternité Sud Réunion<br>- Prématurité < 33 SA<br>- Alcoolisation prénatale authentifiée par un syndrome d'alcoolisation fœtale<br>- Déficience intellectuelle ou épilepsie secondaire d'origine autre que l'infection à CHIKV (causées par les ACSOS ou toute autre cause d'agression cérébrale d'origine inflammatoire, métabolique ou infectieuse)<br>- Absence d'affiliation à un regime de sécurité sociale<br>- Chikungunya néonatal de transmission postnatale
**STRATEGIES/PROCEDURES DE LA RECHERCHE**	Les groupes exposés (enfants infectés par transmission materno-fœtales) et non exposés (enfants appariés sur les principaux facteurs de confusion périnataux) seront comparés en termes de bilan ophtalmologique (motilité oculaire, acuité visuelle et rétinopathie), et neuropsychologique (quotient intellectuel total et subscores, fonctions exécutives, comportement)
**TAILLE D'ÉTUDE**	42 participants : 21 exposés – 21 non-exposés

**DURÉE DE LA RECHERCHE**	– Durée de la période d'inclusion : 18 mois<br>– Durée de participation de chaque participant : 6 mois<br>– Durée totale de la recherche : 24 mois
**ANALYSE STATISTIQUE DES DONNÉES**	Les tests statistiques seront appliqués avec une formulation bilatérale ou unilatérale le cas échéant (dans les analyses de sous groups notamment pour différencier encéphalites/encéphalopathies et prostrations) et un risque α de 5 %. Dans ce cas, une méthode corrective (Bonferroni, Duncan) sera appliquée au besoin pour prendre en compte la multiplicité des tests effectués et maintenir le risque d'erreur de première espèce à 5 %.
**RETOMBÉES ATTENDUES**	Grâce à la confirmation et la caractérisation des conséquences graves d'une infection à CHIKV par transmission verticale materno-fœtale, cette étude permettra d'avoir des implications pratiques concrètes dans la prévention et la surveillance des épidémies d'arboviroses dans les pays où a déjà sévi le chikungunya (avec un risque de réémergence dans départements français d'outremer dont la Réunion, siège de réémergences en 2009 et 2010). Elle permettra également d'attirer la vigilance sur les patients rentrant de voyage en zone endémique, et celle de tous les acteurs de santé publique travaillant dans les nouvelles zones candidates à une transmission autochtone notamment en France métropolitaine, faisant actuellement l'objet d'une surveillance renforcée par Santé Publique France.

2. JUSTIFICATION SCIENTIFIQUE ET DESCRIPTION GÉNÉRALE

2.1. ETAT ACTUEL DES CONNAISSANCES SUR LA PATHOLOGIE

Epidémiologie

Le virus *chikungunya* (CHIKV) est un **arbovirus** (virus transmis par des arthropodes) appartenant à la famille des *togaviridae (*genre *alphavirus)*. Le virus est transmis à l'homme par piqûre de moustiques. Les vecteurs sont des moustiques femelles du genre *Aedes* (ou moustiques Tigres dans le langage commun), reconnaissable par la présence de rayures noires et blanches. Les deux espèces incriminées sont *Aedes albopictus*, principalement présent à la Réunion mais également dans le sud de la France et *Aedes aegypti* présent aux Antilles, en Guyane en Polynésie Française et Nouvelle Calédonie (Reiter P, 2006).

Ces deux moustiques sont également impliqués dans la transmission d'autres arbovirus tels que la dengue, la fièvre jaune et le virus *zika* (Powell & Tabachnick 2013).

Ce virus a été isolé pour la première fois en Tanzanie en 1952 lors d'une épidémie survenue en Afrique de l'Est (Ross 1956). Apparu aux Comores fin 2004, le virus a atteint les îles de l'Océan Indien en 2005. L'île de la Réunion a connu alors une grande épidémie au virus du *chikungunya* durant 17 mois entre 2005 et 2006 avec plusieurs centaines de milliers de cas déclarés, transmis par l'*Aedes albopictus* qui s'y est répandu grâce à sa grande plasticité écologique puisqu'il colonise indifféremment les zones urbaines et selvatiques, les gîtes artificiels et naturels (Renault et al., 2007; Gérardin et al., 2008).

Depuis, le CHIKV continue de circuler sur un mode endémo-épidémique sur les continents Africains (principalement en Afrique centrale et de l'Est) et Asiatique (en particulier aux Indes en 2006). En 2007, la maladie a fait son apparition en Europe, avec une première émergence en août 2007 au Nord-Est de l'Italie dans la province d'Émilie-Romagne (257 cas dont douze enfants, à partir d'un voyageur en provenance du Kerala) (Rezza et al., 2007). Les premiers 12 **cas** autochtones observés dans le Sud de la France ont été recensés dans le Var en 2010. En 2011, la Nouvelle-Calédonie a été touchée, fin 2013 le *chikungunya* s'est propagé aux Antilles puis au reste de la Caraïbe et le continent américain, enfin en Polynésie Française en 2014 (Aubry 2012).

Plus récemment, le CHIKV a ré-émergé en Italie, avec plusieurs clusters dans deux régions (le Lazio et la Calabria) responsables d'une centaine de cas (Trentini et al., 2017), ainsi que dans le Var (Calba et al., 2017). Le risque de réémergence en Europe du Sud fait désormais l'objet d'une surveillance rapprochée par les autorités de santé et l'infection à CHIKV a été ajouté à la liste des maladies à Déclaration Obligatoire depuis janvier 2006.

Présentation clinique

Classiquement chez l'adulte comme chez l'enfant, l'infection se traduit par une phase d'incubation de 4 à 7 jours avec une fièvre élevée à 40°C, des arthralgies souvent extrêmement invalidantes concernant principalement les poignets, les doigts, les chevilles et les pieds mais également les genoux**,** les hanches et les épaules. D'ailleurs, l'appellation « *chikungunya* » vient du Swahili et signifie « l'homme qui marche courbé » évoquant la posture adoptée par les patients atteints en raison d'intenses douleurs articulaires (Aubry 2012). A cette atteinte articulaire s'associent fréquemment des céphalées, la présence de courbatures musculaires et parfois une éruption cutanée maculo-papuleuse (Gérardin et al. 2011). C'est depuis l'épidémie sur l'île de la Réunion, que la communauté médicale s'est aperçue de la gravité potentielle des infections à *chikungunya*, qui était jusqu'alors considérée comme une infection bénigne (Economopoulou A et al., 2009; Gérardin et al., 2011). Elle peut ainsi prendre des formes graves avec notamment des cas d'atteinte

neurologiques sévères tels que des méningo-encéphalites ou des atteintes des nerfs périphériques (syndromes de Guillain-Barré) mais également d'autres formes systémiques graves comme des hépatites aigues ainsi qu'un risque absolu de mortalité non négligeable d'après une récente méta-analyse datée de 2018 (Contopoulos-Ionnanidis D et al., 2018). Ces dernières sont principalement rencontrées chez des personnes âgées, ou au système immunitaire affaibli, et chez des nouveau-nés, infectés *in utero* alors de l'infection de la mère (Tandale et al., 2009;Gérardin et al., 2011).

<u>Particularité de l'atteinte néonatale par transmission verticale materno-fœtale</u>

Au cours de l'épidémie ayant débuté en 2005 sur l'île de la Réunion, il a été mis en évidence pour la première fois des cas **d'infections néonatales par transmission verticale** materno-fœtale du CHIKV (Robillard et al., 2006;Ramful et al., 2007;Gérardin P et al., 2008). Cette transmission materno-fœtale peut survenir à un terme précoce de la grossesse, avant 22 SA. Elle apparaît cependant rare, mais létale à ce terme avec des cas de morts fœtales in utéro (MFIU) décrit dans plusieurs études. La présence du génome viral dans le liquide amniotique et dans le placenta attestait de la transmission transplacentaire du CHIKV. Il n'y a pas de cas de transmission materno-fœtale après 22 SA décrit à ce jour dans la littérature. L'infection virale au CHIKV au cours des 2èmes et 3èmes trimestres de grossesse semble donc bénigne. Aucune MFIU imputable au virus n'a été retrouvée en milieu de grossesse et les taux de prématurité et de retard de croissance intra utérin ne sont pas modifiés par rapport à la population générale des accouchées. Aucune embryopathie ou fœtopathie n'est décrite, le chikungunya ne semble donc pas être tératogène (Touret et al., 2006;Lenglet et al., 2006). En revanche, l'infection à CHIKV au terme de la grossesse en période de virémie maternelle est à haut risque pour le nouveau-né. Sur l'ensemble des femmes présentant une hyperthermie dans les 48 heures précédant l'accouchement et le jour même, celles qui avaient une infection biologiquement prouvée par PCR présentaient un risque absolu de transmission au fœtus élevé (50,3%) d'après la récente méta-analyse de Contopoulos-Ioannidis (Contopoulos-Ioannidis et al. 2018).

Le mécanisme physiopathologique de la transmission materno-fœtale n'a pas encore été élucidé. Le fait que les nouveau-nés malades soient tous issus de mère en période de virémie au moment de l'accouchement, l'absence ou la très faible permissivité du syncitiotrophoblaste chez la souris gestante (Couderc et al., 2007; Platt et al., 2018), ainsi que la mise en évidence d'un délai constant entre la naissance et le début des symptômes, ont conduit à émettre l'hypothèse d'une contamination per-partum de l'enfant sans que l'on puisse en comprendre le mécanisme. L'absence de protection en cas de naissance par césarienne était un argument contre une possible contamination vaginale lors du passage par la filière génitale. Actuellement, l'hypothèse d'une contamination en pré-partum immédiat (plutôt qu'en per-partum), et donc par voie transplacentaire, est plus volontiers privilégiée et ce, en raison de deux arguments : le taux de césarienne pour souffrance fœtale chez ces enfants est nettement plus élevé et la recherche de virus au niveau placentaire est constamment positive lorsqu'elle a été réalisée. Nous ne pouvons cependant pas exclure une deuxième hypothèse: celle que la transmission du CHIKV se fasse lors des échanges sanguins qui existent au moment de l'accouchement entre la mère et l'enfant (Gérardin et al., 2008;Contopoulos-Ioannidis et al., 2018).

Les présentations cliniques chez le nouveau-né pouvaient être sévères d'emblée (encéphalite ou encéphalopathie, fièvre hémorragique) ou compliquées d'état de choc (Gérardin et al., 2008).

La présentation la plus commune était principalement d'ordre neurologique à type de prostration douloureuse et fébrile (hypotonie marquée, difficultés à téter, irritabilité) (Gérardin et al. 2014).

Les **conséquences à moyen terme** de ce virus à fort neurotropisme ont été étudiées chez 33 enfants à l'âge de 2 ans dans l'étude CHIMERE ainsi qu'à l'âge de 5 ans sur une petite cohorte

d'enfants suivi au C.A.M.S.P (Centre d'Action Médico-Sociale Précoce) du Sud de l'île de la Réunion (Boumahni & Bintner, Med Trop 2012; Gérardin et al., 2014) .

Prise en charge actuelle d'une infection et prévention

La prise en charge médicale de l'infection néonatale à CHIKV est **symptomatique**, reposant sur des traitements anti-douleurs de palier I et II voir III chez les plus algiques. La surveillance de ces nouveau-nés s'est faite systématiquement à l'hopital dans des unités spécialisées de soins intensifs ou de réanimation selon la présentation clinique. L'utilisation d'amines en cas de choc ou un support ventilatoire était parfois nécessaire ainsi que l'utilisation d'un traitement anti-convulsivant en cas d'épilepsie. Les enfants ayant contracté une infection à CHIKV en période néonatale, ont tous bénéficié d'un suivi médical spécialisé jusqu'à l'âge de 6 ans.

La prévention de cette infection est à la fois collective et individuelle. Certaines études ont envisagé la prévention des formes néonatales par des mesures obstétricales empiriques comprenant l'utilisation de tocolytiques pour retarder l'accouchement le temps que la mère produise des anticorps neutralisants et les passe à l'enfant par voie trans-placentaire. Effectivement, le CHIKV pourrait être un inducteur d'une réponse immune chez la femme enceinte, avec un relargage de médiateurs de l'inflammation tels que le TNF alpha susceptible de déclencher le travail. La transmission transplacentaire au cours de cette période, pourrait être responsable de la survenue de souffrances fœtales per-partum et entraînerait un taux de contamination élevé du fœtus. C'est à partir de cette hypothèse qu'a été suggéré que l'utilisation de tocolytiques pour retarder l'accouchement le temps que la mère produise des anticorps neutralisants transplacentaires, pourrait diminuer le risque de transmission materno-fœtale (Gérardin et al., 2008;Escobar Vidarte et al., 2017;Contopoulos-Ioannidis et al., 2018). Par ailleurs, une étude prometteuse bien que non concluante sur le modèle animal, partait du postulat que l'administration d'immunoglobulines CHIKV pouvait constituer une stratégie sécuritaire et efficace de prévention et traitement pour les personnes exposées à CHIKV courant le risque d'infection grave, comme chez les nouveau-nés nés de mères virémiques (Couderc et al. 2009). La césarienne en tout début de travail, avant que l'intensité des contractions utérines ne soit susceptible de provoquer des brèches placentaires, n'a pas montré d'effet protecteur sur l' infection à CHIKV chez le nouveau né (Lenglet et al., 2006; Gérardin et al., 2008).

Etant donné l'absence de démonstration d'efficacité dans ces différentes études, la prévention repose essentiellement à ce jour sur la **lutte anti-vectorielle chez la femme enceinte.**

2.2. HYPOTHESES DE LA RECHERCHE ET RESULTATS ATTENDUS

Dans les cas de MFIU décrits dans la littérature au 1er trimestre de grossesse, le génome du CHIKV avait été retrouvé dans le liquide amniotique et dans le placenta alors qu'il n'était plus détecté dans le sang maternel (RT-PCR CHIKV négative). Ceci excluait la contamination du liquide amniotique par le sang maternel lors de l'amniocentèse et celle du placenta au moment de la délivrance. De plus, les autopsies retrouvaient la présence de génome du CHIKV dans le cerveau fœtal. Chez les nouveau-nés atteints de CHIKV prouvé biologiquement, 21% ont présenté des convulsions durant leur hospitalisation à type d'apnées prolongées avec des troubles sévères de la conscience. Chez certains de ces enfants, les IRM cérébrales étaient anormales avec un hypersignal très marqué de la substance blanche au niveau des deux hémisphères cérébraux en séquence de diffusion, évocateur d'œdème cytotoxique. De plus chez la plupart des enfants symptomatiques sur le plan neurologique, la RT-PCR CHIKV était positive dans le LCR.

L'ensemble de ces arguments cliniques, biologiques, anathomopathologiques et radiologiques suggèrent un fort neutropisme du CHIKV (Lenglet et al., 2006;Gérardin et al., 2014;Gérardin et al., 2016; Mehta, Gérardin et al., 2018). Ce neurotropisme a été largement confirmé in vitro et in vivo. Il touche particulièrement les astrocytes, à moindre part les neurones, plus inconstamment infectés dans les modèles cellulaires ou animaux (Das et al., 2010; Das et al., 2015; Lim et al., 2017). Les cellules du plexus choroides et des leptoméninges hypervascularisées constituent la voie d'accès au système nerveux central (Couderc et al., 2008). Une fois infectées, les cellules cibles seraient le siège d'une intense mort cellulaire par apoptose, nécronoptose ou pyroptose (Das et al., 2015; Lim et al., 2017). La signature génique moléculaire serait commune à celle d'autres infections neuroinvasives du SNC dont l'encéphalite à virus du Nile occidental (Lim et al., 2017). Elle partagerait en outre certaines signatures retrouvées dans les maladies neurodégénératives, dont la sclérose en plaques ou la maladie de Parkinson (Lim et al., 2017).

La majorité du développement cérébral se déroule in utero et jusqu'à l'âge de deux ans. Nous savons aussi que grâce à la plasticité cérébrale de l'enfant, les stimulations de l'environnement et les apprentissages guident la mise en place des circuits neuronaux permettant d'assurer les grandes fonctions cérébrales, qu'elles soient motrices, sensorielles ou cognitives. Les IRM cérébrales précoces réalisées peu après la naissance et dans la première année de vie, ont montré une atteinte diffuse de la substance blanche avec raréfaction de la myéline, prédominante dans les zones préfrontales, incluant les zones germinatives péri-ventriculaires impliquées dans la réparation tissulaire et la migration neuronale qui l'accompagne (Gérardin et al., 2014).

Sur la base de ces résultats en période d'épidémie chez ces nouveaux nés, ainsi que des résultats de l'étude CHIMERE acquis en moyenne vers l'âge de 2 ans, nous émettons en première hypothèse que les enfants infectés à la naissance par le CHIKV auront des **séquelles cliniques, neurosensorielles et neurocognitives** vers l'âge de 13 ans altérant leur performance neurodéveloppementale, leur comportement et leur scolarité, notamment des troubles de la coordination, du langage et du raisonnement perceptif et conceptuel. Notre hypothèse secondaire est que ces troubles seront liés à une perturbation des circuits neuronaux affectant les zones cérébrales responsables des fonctions concernées, et que ces zones cérébrales pouvaient être identifiées par une imagerie cérébrale précoce.

2.3. JUSTIFICATION DES CHOIX METHODOLOGIQUES

2.3.1. JUSTIFICATION DU SCHEMA DE LA RECHERCHE

Le recours à une étude comparative de type exposé - non exposé est justifié par le fait que les critères de jugement (performance neurodéveloppementale, troubles du comportement, recours à des soins spécialisés, scolarisation, etc…), ne sont pas connus au démarrage de l'étude.

L'utilisation pour le groupe exposé d'une cohorte historique déjà bien documentée minimisera le biais d'information tout en permettant d'assurer un suivi longitudinal des enfants infectés à la naissance par transmission materno-fœtale du CHIKV.

Il ne s'agit pas d'une étude prospective à proprement parler réalisée à partir des seuls enfants infectés à la naissance, mais plutôt d'une étude à visée pronostique comparant un groupe d'intérêt, infecté, à un groupe contrôle le plus pur (donc non infecté) afin d'obtenir le meilleur contraste possible dans les comparaisons.

Le caractère monocentrique de l'étude est justifié par l'absence de population d'enfants exposés comparable. Certes, le chikungunya a circulé un peu partout dans le monde depuis 2004, mais aucune population n'a réuni de cohorte importante disposant du même recul pour une évaluation du neurodéveloppement à l'âge de la pré-adolescence.

2.3.2 JUSTIFICATION DU CRITERE DE JUGEMENT PRINCIPAL

Le quotient intellectuel total (QIT) de l'échelle WISC-V (Wechsler Intelligence Scale for Children 5th version) est l'outil le plus utilisé dans le monde pour mesure l'efficience intellectuelle des enfants âgés de 6 à 16 ans. Son choix en tant que critère de jugement principal permettra de comparer le pronostic neurodéveloppemental vers l'âge de 13 ans des enfants infectés à la naissance par transmission verticale materno-néonatale du CHIKV aux populations d'enfants sur lesquelles cet outil a été développé ainsi que d'autres populations d'enfants vulnérables (grands prématurés, SAF, etc…) sur lesquelles il a pu être utilisé. Ces comparaisons permettront de vérifier la cohérence des résultats avec les autres populations d'enfants présentant des déficiences cognitives, des troubles du comportement, des difficultés d'apprentissage ou un retard scolaire.

2.3.3 JUSTIFICATION DU TYPE DE COMPARAISON

Les biais de sélection et de confusion seront pris en compte.

L'effet de sélection sera étudié tant dans le groupe exposé (comparaison des 21 enfants infectés participants aux 12 autres enfants ayant complété l'évaluation neurodéveloppementale dans la cohorte CHIMERE sur les données périnatales, les données de suivi et le pronostic neurodéveloppemental à deux ans) que dans le groupe contrôle (comparaison des 21 enfants non infectés participants aux autres enfants appariés et à la population source) dont on vérifiera pour chacun la représentativité.

L'effet de confusion sera contrôlé à l'inclusion par un appariement sur de multiples facteurs (détaillé au **chapitre 5.2**). Le but de cet appariement multiple est d'équilibrer les tiers facteurs et de favoriser une interprétation causale (« la déficience est due à l'infection néonatale à CHIKV »), toute chose étant égale par ailleurs, celle-ci n'étant admise cependant qu'après randomisation.

La population à apparier sera sélectionnée au sein de la cohorte maternelle de l'étude CHIMERE (Fritel, et al., 2010) et dans le relevé épidémiologique périnatal des maternités sud Réunion. En effet, en 2006, il n'existait pas de recueil de données périnatales dans les autres maternités de la Réunion susceptible de servir de population source pour être appariée. De nombreuses publications ont été réalisées sur le relevé épidémiologique périnatal des maternités du sud Réunion. Leur liste est consultable dans les rapports annuels de ce relevé sur le site de réseau périnatal Réunion (RePèRe) https://www.repere.re/professionnels/bibliotheque/publications-regionales.html?L=4

2.3.4. JUSTIFICATION DE LA CONDUITE DE LA RECHERCHE

Cette recherche est très attendue et ne peut être conduite qu'à l'île de la Réunion (cf. **chapitre 2.5**), seule région disposant du recul nécessaire pour étudier à l'âge de la pré-adolescence le pronostic neurodéveloppemental d'une infection néonatale par transmission verticale materno-fœtale du CHIKV. Plusieurs acteurs de ce protocole ont participé à la cohorte CHIMERE dont ils ont contribué à publier les résultats. Cet élément offre une garantie de publication des résultats.

2.4. RAPPORT BÉNÉFICE / RISQUE

Les enfants classés comme déficients (QIT du WISC-V < 70) se verront proposer une prise en charge en ALD de leur handicap et un suivi scolaire et médical spécialisé.

Les parents dont l'enfant présentera des troubles cognitifs ou comportementaux diagnostiqués au moment de l'étude se verront proposer une intervention correctrice pour leur enfant, soit au Centre

Médico-Psycho-Pédagogique (CMPP), soit à l'Institut Médico-Educatif (IME), soit par le Service d'Education Spéciale et de Soins à Domicile (SESSAD), de l'île, dans la mesure des places disponibles. Celle-ci consistera en un programme personnalisé, adapté à chaque enfant, conforme aux données de la science. Il s'agit donc d'une étude à bénéfice direct pour l'enfant dépisté.

Nous ne concevons pas de risque lié à ce type d'étude, si ce n'est celui inhérent au déplacement. Le risque de stigmatisation d'enfants légèrement déficients par un entourage familial inadéquat paraît en effet négligeable compte tenu de l'historique des enfants antérieurement suivis au CAMSP ou au CMPP.

Les trois visites prévues au suivi pourraient théoriquement générer des perdus de vue, notamment dans le groupe contrôle possiblement moins motivé par l'étude, mais nous estimons raisonnable que ce risque pourra être compensé par la gratuité du bilan neuropsychologique et ophtalmologiques offerts au titre de l'étude.

L'investigateur principal surveillera, évaluera et documentera les risques tout au long du suivi et s'assurera qu'ils pourront être gérés de manière satisfaisante.

2.5. RETOMBÉES ATTENDUES

Aucune étude n'a proposé un suivi à l'âge scolaire des enfants qui avaient été infectés par le virus *chikungunya* (CHIKV) par voie materno-foetale. Grâce à la confirmation et la caractérisation des conséquences graves d'une infection à CHIKV par transmission verticale materno-fœtale, cette étude permettra d'avoir des implications pratiques concrètes dans la prévention et la surveillance des épidémies d'arboviroses dans les pays où a déjà sévi le *chikungunya* (avec un risque de réémergence dans départements français d'outremer dont la Réunion, siège de réémergences en 2009 et 2010). Elle permettra également d'attirer la vigilance sur les patients rentrant de voyage en zone endémique, et celle de tous les acteurs de santé publique travaillant dans les nouvelles zones candidates à une transmission autochtone notamment en France métropolitaine, faisant actuellement l'objet d'une surveillance renforcée par Santé Publique France.

- Mise en place de mesures concrètes en prévention primaire à l'échelle individuelle et collective afin de diminuer l'incidence de la maladie : par l'éducation et l'information auprès de la population générale et du corps médical pour promouvoir des mesures de protection anti-vectorielles et des campagnes d'information du risque chez les femmes enceintes en cas d'épidémie. Par ailleurs, la mise en place d'épandages précautionneux d'insecticides et une élimination des gîtes larvaires potentiels par mobilisation communautaire, particulièrement autour des habitations est nécessaire.
- Mise en place de mesure en prévention secondaire : surveillance renforcée par les néonatologues d'enfants CHIK+ pour renforcer la neuroprotection chez ces enfants par le contrôle des ACSOS afin de diminuer les co-morbidités notamment neurologiques, qui pourraient être associé**es** aux soins.
- Prévention tertiaire : diminution des incapacités fonctionnelles et de l'invalidité des enfants atteints, par une prise en charge adaptée précoce fondée sur une amélioration du suivi pédiatrique et une meilleure rééducation psychomotrice, neurosensorielle et neurocognitive favorisant l'insertion scolaire, professionnelle et sociale.

Ces trois mesures de prévention auront un impact direct pour les patients, et la santé publique.

Le projet CHIK13+ est un projet collaboratif pluridisciplinaire à haut potentiel de valorisation scientifique. En effet, l'étude à long terme du neuro-développement d'enfants affectés par une arbovirose congénitale au travers d'un suivi longitudinal à plus de dix ans d'une épidémie sans précédent est unique en son genre. La publication qui découlera de cette étude pilote devrait

continuer d'inscrire la recherche académique réunionnaise à la pointe de la recherche internationale sur cette thématique.

2.6. JUSTIFICATION DU FAIBLE NIVEAU D'INTERVENTION

Cette étude a été classée comme recherche à risques et contraintes minimes car conformément à l'arrêté du 3 mai 2017, les interventions prévues au protocole présentent des entretiens et questionnaires pouvant mettre en jeu la securite de la personne ou conduire à la modification de sa prise en charge habituelle et ne relevant pas de ce fait de la recherche non interventionnelle.

3. OBJECTIFS DE LA RECHERCHE

3.1 OBJECTIF PRINCIPAL

Evaluer à l'aide du Quotient Intellectuel Total (QIT) de l'échelle WISC-5 (*Wechsler Intelligence Scale for Children 5th version*) vers l'âge de 13 ans la performance neurocognitive d'enfants infectés à la naissance par transmission materno-fœtale du CHIKV en comparaison de celle d'un groupe d'enfants non infectés, sélectionnés dans le relevé épidémiologique périnatal des maternités pour maitriser l'exposition, ceux-ci étant appariés sur les principaux facteurs de confusion.

3.2. OBJECTIFS SECONDAIRES

- Evaluer la compréhension verbale, le raisonnement visuo-spatial et le raisonnement fluide, la mémoire de travail et la vitesse de traitement, à l'aide du profil des notes standards et des notes composites des cinq échelles psychométriques ou indices spécifiques (dimensions) composant le WISC-5 (respectivement ICV, IVS, IRF, IMT, IVT)

- Evaluer les fonctions exécutives à l'aide du score total composite et des indices spécifiques des quatre domaines de l'échelle VABS-II (*Vineland Adaptive Behavior Scale 2ème version*) à savoir : communication, autonomie dans la vie quotidienne, socialisation et motricité.

- Evaluer le comportement et les troubles du comportement à l'aide du questionnaire subsidiaire de l'échelle VABS-II et des questionnaires SDQ (*Strength and Difficulties Questionnaire)*. Le questionnaire des troubles du comportement de l'échelle VABS-II permet un dépistage des troubles autistiques tandis que les questionnaires SDQ permettent d'évaluer l'enfant dans son milieu familial, à l'école ainsi qu'une auto-évaluation de l'enfant par rapport à son environnement.

- Evaluer les capacités et les déficits sensoriels auditifs et visuels à l'aide d'une audiométrie tonale et d'une acuité visuelle

- Evaluer la scolarité de l'enfant (scolarité adaptée pour l'âge au collège, avec ou sans soutien scolaire, années redoublées ; scolarité en institution ou école spécialisée ; recours au CLIS, SESSAD, etc…)

- Evaluer le recours aux soins spécialisés suivants : orthophonie, orthoptie, psychomotricité, orthopédie, kinésiethérapie et rééducation fonctionnelle

4. CRITERES DE JUGEMENT

4.1. CRITÈRE DE JUGEMENT PRINCIPAL

Quotient intellectuel total (QIT) de l'échelle WISC-V (*Wechsler Intelligence Scale for Children 5th version*).

La WISC-V est l'outil le plus utilisé dans le monde pour mesure l'efficience intellectuelle des enfants âgés de 6 à 16 ans. Il évalue le fonctionnement intellectuel global à l'aide du QIT, résultat de cinq indices qui sont eux-mêmes élaborés à partir de sept subtests principaux et huit épreuves supplémentaires facultatives. Ces subtests font appel à des processus mentaux complexes. Chacun est composé d'items (c'est-à-dire de questions), rangés par difficulté croissante. Il y a entre vingt et trente items selon les subtests. Au bout de plusieurs items échoués, l'épreuve s'arrête et le/la (neuro)-psychologue fait passer l'enfant au subtest suivant. Et ainsi de suite jusqu'à la fin de la passation. Les cinq indices sont l'Indice de Compréhension Verbale (ICV), l'Indice Visuo-Spatial (IVS), l'Indice de Raisonnement Fluide (IRF), l'Indice de Mémoire de Travail (IMT) et l'Indice de Vitesse de Traitement (IVT).

La répartition des résultats se fait selon une courbe de distribution répondant à la loi Normale de l'intelligence, également appelée courbe de Gauss. La moyenne est établie à 100, avec un écart-type de 15 points. On considère hors norme des scores s'éloignant de deux écarts-types en dessous ou au dessus de cette moyenne.

<u>La répartition exacte est la suivante :</u>
- de 2,2 à 2,3% de la population présente un QI Total ≤ 70 et donc, un retard mental (dont 0,13% présente un QI Total ≤ 55)
- 95% de la population présente un QI Total compris entre 70 et 130 (de l'intelligence faible à l'intelligence supérieure, en passant par la norme, norme qui peut être moyenne basse ou moyenne haute)
- de 2,2 à 2,3% de la population présente un QI Total ≥ 130 et donc un haut potentiel intellectuel (dont 0,13% présente un QI Total ≥ 145, pouvant être qualifié de Très Haut Quotient Intellectuel. Soit à peine plus d'1‰).

Le WISC-V a été étalonné sur 1100 enfants et adolescents.
Le score de QI est un chiffre relatif. Le QIT est ainsi obtenu par comparaison à l'échantillon témoin de ces 1100 jeunes qui ont permis d'étalonner le test avant sa sortie officielle.

4.2. CRITÈRES DE JUGEMENT SECONDAIRES

Indices spécifiques des 5 dimensions du WISC-V permettant de mesurer respectivement la compréhension verbale (ICV), l'indice visuo-spatial (IVS), le raisonnement fluide (IRF), la mémoire de travail (IMT) et la vitesse de traitement (IVT), à l'aide du profil de leurs notes standards et de leurs notes composites, de la détermination des points forts et des points faibles aux subtests, de l'analyse des notes additionnelles et de la comparaison des différences.

Une déficience légère spécifique à un domaine du neurodéveloppement est définie pour un score compris entre 70 et 79. Une déficience spécifique sévère est définie pour un score inférieur à 70.

- Score total composite (moyenne 100 et déviation standard ± 15) de l'échelle VABS-II (*Vineland Adaptive Behavior Scale 2nd version*) et indices spécifiques de ses quatre

domaines (moyenne 15 et déviation standard ± 3) : communication, autonomie pour les gestes de la vie quotidienne, socialisation, motricité.

Une dysfonction exécutive est définie pour un score total composite du VABS-II compris entre 70 et 85. Une déficience exécutive est définie pour un score total composite inférieur à 70. Une dysfonction exécutive spécifique est définie pour un score spécifique inférieur à 9.

- Trouble (oui/non) du comportement identifié par les 27 questions additionnelles du VABS-II

- Scores de difficultés totales et scores des quatres premiers indices (activité, problèmes de conduite, émotions, problèmes avec les pairs) du SDQ (*Strength and Difficulties Questionnaire*) validé chez les enfants âgés de 4 à 16 ans.

Il existe trois versions de ce questionnaire, l'une sous forme d'auto-questionnaire pour l'enfant, l'autre destinée aux parents, et enfin la dernière, plus complète destinée aux enseignants, pour évaluer le comportement des enfants dans leur milieu familial et à l'école.

Ces évaluations psychométriques seront réalisées par une neuropsychologue expérimentée.

- Troubles de la convergence ou de la motilité oculaire (hétérophorie, voire strabisme) et déficit de l'acuité visuelle (oui/non) et si ouiport de lunettes (oui/non)

- Type d'école fréquentée en catégoriel (collège normal/institution ou école spécialisée) ; Niveau de scolarité (classe adpatée pour l'âge sans soutien scolaire/classe adaptée pour l'âge avec soutien scolaire/retard scolaire avec redoublement)

- Recours à des soins spécialisés : orthophoniste (oui/non) ; orthoptiste (oui/non) ; psychomotricien (oui /non); kinésithérapie et rééducation fonctionnelle (oui/non)

5. CONCEPTION DE LA RECHERCHE

5.1. SCHÉMA DE LA RECHERCHE

Il s'agit d'une étude comparative de cohorte de type exposé - non exposé appariée à visée pronostique, monocentrique. L'exposition d'intérêt correspond à une infection néonatale par transmission verticale materno-fœtale du CHIKV survenue à l'accouchement (femme virémique ou symptomatique entre 2 jours avant et 2 jours après l'accouchement), définie par une RT-PCR et/ou une sérologie Mac Elisa IgM néonatale positive(s) dans les 10 jours suivant la naissance (jusqu'à 15 jours dans le LCR).

Ces enfants ont fait l'objet d'un suivi tant sur le plan clinique ou développemental que sérologique dans la cohorte CHIMERE (constituée entre avril et juillet 2006 de manière ambispective par inclusion rétrospective des cas d'infection néonatale survenus par transmission materno-fœtale avant avril 2006 et par inclusion prospective des couples mères enfants à partir d'avril 2006).

5.2. METHODES POUR L'APPARIEMENT DES NON-EXPOSES

Pour réduire les possibilités de biais de sélection susceptible d'altérer la relation entre l'infection et le pronostic neurodéveloppemental, les enfants exposés, c'est à dire les enfants infectés à la naissance par le CHIKV (groupe d'intérêt) seront appariés à des enfants non exposés (groupe contrôle) dont la mère n'a été pas infectée pendant la grossesse. Pour les mères infectées à l'accouchement, nous excluerons de l'appariement les enfants connus pour avoir fait un chikungunya néonatal de transmission postnatale.

L'appariement vise à équilibrer les tierces variables entre le groupe d'intérêt et le groupe contrôle et tendre à ce que ces deux groupes ne diffèrent que par la variable d'exposition (infection néonatale par transmission verticale materno-foetale). Plus un appariement comporte de variables, plus il a de chances de minimiser la confusion résiduelle et d'équilibrer les tiers facteurs non mesurés dans les deux groupes, ceux-ci étant eux-mêmes liés à des variables mesurées également réparties, mais plus il est difficile à réaliser.

Le groupe non exposé correspondra donc à une population d'enfants sains nés pendant l'épidémie à l'île de la Réunion, appariés à la population sur l'âge maternel, le niveau d'étude maternel, le mois de naissance, le sexe, l'âge gestationnel, le poids de naissance à partir de la base du relevé épidémiologique périnatal des maternités de la Réunion et de la cohorte CHIMERE. La prise en compte dans cet appariement du poids de naisance permettra par exemple de limiter l'influence du SAF (syndrome d'alcoolisation fœtale) et de l'ETCAF (ensemble des troubles causés par l'alcoolation fœtale) dont les incidences et prévalences sont élevées à l'île de La Réunion (Serreau et al., 2002; Lamblin et al., 2008), ainsi que celles de l'embryofoetopathie à cytomegalovirus (CMV). L'infection néonatale à CMV peut effectivement présenter certains signes similaires à une infection au CHIKV. Cependant, le tableau clinique est souvent différent avec notamment un retard de croissance intra utérin marqué, qui est une manifestation fréquente (Pereira L et al., 2014) et des séquelles à distance centrées sur des déficiences auditives, avec une intelligence normale et des lésions de la substance blanche profonde en IRM (James F et al., 2012) (Nakagawa K et al., 2018).

Chaque enfant exposé sera préalablement apparié à plusieurs enfants non exposés d'abord par la méthode d'appariement individuel après stratification des deux groupes sur les variables précédentes puis, en cas d'échec, par la méthode de la distance de Mahalanobis qui mesure la similarité de chaque observation comme une variable continue, enfin certaines variables ayant été identifiées dans la cohorte CHIMERE comme facteurs de confusion (à la fois liés à l'infection maternelle et connues pour être liées au pronotic neurodéveloppemental, comme le niveau

d’éducation maternelle, un score de déprivation ou l’âge gestationnel), si la méthode précédente ne parvient à équilibrer les deux groupes, un appariement sur le score de propension sera envisagé (celui donnant une équiprobabilité d’être exposé). Celui-ci présuppose de ne pas exclure les mères infectées à l’accouchement qui n’ont pas transmis le virus verticalement à leur enfant.

Une fois l’appariement effectué un enfant contrôle unique sera sélectionné parmi les non exposés éligibles volontaires et par tirage au sort si plusieurs enfants contrôles ont été proposés.

6. CRITERES D'ELIGIBILITE

6.1. CRITERES D'INCLUSION

- Enfant né entre mars 2005 et juillet 2006
- Dont la mère identifiée dans la cohorte CHIMERE ou le registre perinatal des maternités du Sud Réunion
- Exposé : enfant infecté par le virus chikungunya au moment de l'accouchement
- Non exposé : enfant non infecté par le virus chikungunya au moment de l'accouchement vérifiant les critères d'appariement précisés au chapitre 5.2
- Affilié à un régime de sécurité sociale

6.2. CRITÈRES DE NON-INCLUSION

- Enfant né avant mars 2005 ou après août 2006
- Mère non identifiée dans la cohorte CHIMERE ou le register perinatal de la maternité Sud Réunion
- Prématurité < 33 SA
- Alcoolisation prénatale authentifiée par un syndrome d'alcoolisation fœtale
- Déficience intellectuelle ou épilepsie secondaire d'origine autre que l'infection à CHIKV (causées par les ACSOS ou toute autre cause d'agression cérébrale d'origine inflammatoire, métabolique ou infectieuse)
- Absence d'affiliation à un regime de sécurité sociale
- Chikungunya néonatal de transmission postnatale

Aucun de ces critères n'avait été initialement identifié dans l'étude CHIMERE. Certaines déficiences et leurs causes pouvant se révéler secondairement, des mois voire des années après la naissance (par exemple, déficience en lien avec une alcoolisation prénatale souvent inavouée), un examen du dossier médical des enfants éligibles sera réalisé pour vérifier qu'aucune de ces conditions n'a été diagnostiquée secondairement.

Le diagramme d’inclusion des exposés est résumé dans la **Figure 1** suivante :

Figure 1: diagramme d'inclusion des exposés

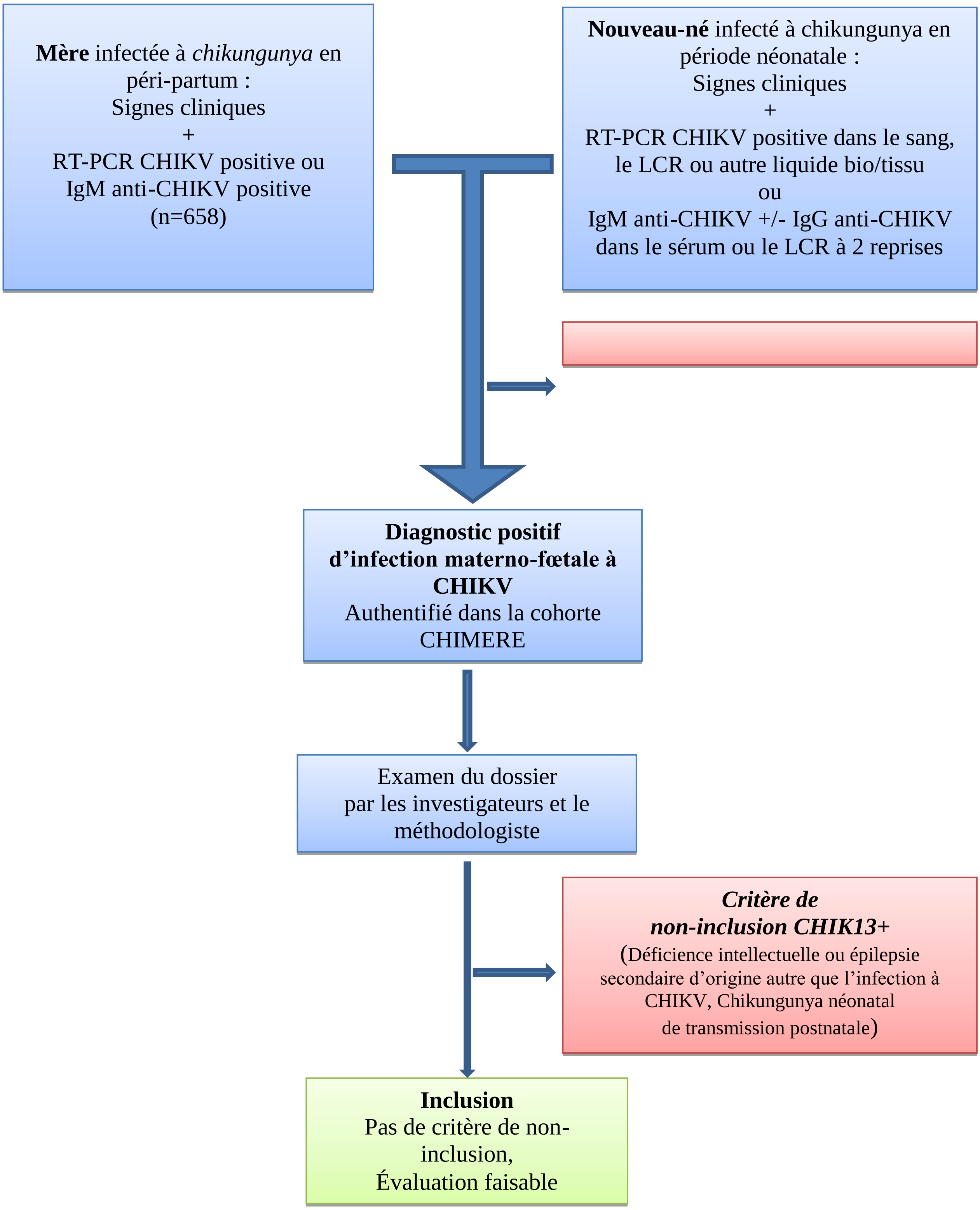

6.3. FAISABILITE ET MODALITES DE RECRUTEMENT

Le recrutement est prévu à partir de la cohorte CHIMERE et des registres des maternités du Sud Réunion, lequel collige de manière continue les données épidémiologiques de tous les couples mères-nouveau-nés accouchés/nés dans le Sud Réunion (maternités du GHSR, Saint Pierre, Saint Louis, Clinique Durieux) depuis janvier 2001. 31 des 33 enfants exposés suivis dans la cohorte CHIMERE étant d'origine réunionnaise, 21 ayant été revus parmi 30 recontactés en septembre 2015, nous estimons le recrutement du groupe exposé comme faisable, voire très faisable. Pour le groupe non exposé, nous envisageons un recrutement à partir des mêmes sources qui comportent toutes les variables suffisantes à un appariement de qualité. Les bases de données sources sont importantes, les populations incluses encore très probablement accessibles, du fait de l'insularité et du caractère relativement captif des populations locales plutôt attachées à leur île jusqu'à la fin du lycée. Enfin, les procédures d'appariement qui seront utilisées sont toutes à fait classiques et maitrisées par le centre de méthodologie, ce qui fait de notre étude un projet faisable.

7. TRAITEMENT(S)/STRATEGIE(S)/PROCEDURE(S) DE LA RECHERCHE

7.1. TRAITEMENT/STRATEGIE/PROCEDURE EXPERIMENTAL(E)

Aucun traitement, stratégie ou procédure expérimentale n'est prévu dans cette étude, s'agissant d'une étude d'observation qualifiée d'interventionnelle impliquant la personne humaine à contraintes minimes (RIPH2).

7.2. TRAITEMENT/STRATÉGIE/PROCÉDURE DE COMPARAISON

Chaque participant aura une sérologie chikungunya IgG par ELISA pour vérifier l'adéquation de son classement en exposé ou non-exposé. Pour les exposés, une sérologie IgM par immunocapture (MAC) ELISA sera également demandée pour vérifier l'absence d'IgM, argument en faveur d'une persistance de la stimulation antigénique, et s'assurer du caractère séquellaire des troubles neurocognitifs et comportementaux éventuellement observés. En réponse à la réaction immunitaire de l'hôte, le virus développe des mécanismes élaborés afin d'éviter, de contrecarrer et de détourner les mécanismes de détection, les voies d'activation ainsi que les différentes fonctions effectrices. Cet équilibre peut tourner à l'avantage du pathogène et contribuer à l'infection chronique et à la persistance virale. Pour ce faire, le virus doit répondre à deux conditions : sa capacité à co-exister dans la cellule hôte de façon non lytique et être capable de développer des stratégies d'évasion qui lui permettent de ne pas être reconnu par le système immunitaire de l'hôte. Le virus ne doit pas exposer ses antigènes pour échapper à sa reconnaissance par les cellules de l'hôte, et/ou se déguiser afin d'être reconnu comme faisant partie du soi (Oldstone 1998). A tous les niveaux de la réaction immunitaire développée par l'hôte, le virus a développé des stratégies afin de la subvertir et ainsi se sanctuariser dans l'organisme hôte. 1) des protéines virales peuvent empêcher la détection du virus par des modificiations cellulaire des protéines de membranes (Sklan, Charuworn et al., 2009). 2) par inhibition de la production et de la voie de signalisation de l'interféron, bien décrite dans le premier modèle murin de chikungunya ou sur des lignées cellulaires (Couderc et al., 2008; Fros et al., 2010; Schilte et al., 2010).

Une persistance des IgM anti-chikungunya très à distance de l'infection primitive serait en faveur d'une persistance de debris viraux, voire de virus vivants sanctuarisés dans l'organisme hôte comme cela a été suggéré pour deux cas d'arthrites à CHIKV (Malvy et al., 2008; Hoarau et al., 2010), confirmant ce qui avait été déjà observé avec d'autres alphavirus : persistance du virus dans le liquide synovial articulaire, le périoste ou les ligaments (Cunningham and al. 1981 ; Soden, Vasudevan et al., 2000), ce qui argumenterait contre l'origine séquellaire des troubles neurocognitifs et comportementaux, et renforcerait l'interprétation causale, le SNC étant connu pour constituer une niche sanctuaire des pathogènes pour échapper à la réponse immunitaire de l'hôte (Das T et al., 2010 ; Miner et al., 2015).

Chaque participant bénéficiera d'un bilan ophtalmologique afin de dépister des complications oculaires à distance de l'infection néonatale à chikungunya.

Rappel des généralités sur le WISC-V

Lorsque qu'une déficience cognitive globale est suspectée, celle-ci doit être confirmée ou infirmée par des tests psychométriques adaptés à l'âge. Les outils de dépistage de ces troubles sont nombreux et ne sont pas spécifiques à une pathologie. Nous avons choisi dans cette étude l'échelle WISC-V (*Wechsler Intelligence Scale for Children 5 th version*) comme précédemment citée, car c'est l'outil le plus utilisé dans le monde pour mesure l'efficience intellectuelle des enfants âgés de 6 à 16 ans. La 5ème version, en vigueur depuis 2016, a été entièrement remaniée dans l'objectif de mieux évaluer les enfants pour un diagnostic plus précis ainsi que de réduire la durée de passation. Cet outil à déjà fait ses preuves dans de nombreuses pathologies à risque de déficience cognitive

globale, tel que dans l'étude de la prématurité, dans des atteintes du SNC comme l'agénésie du coprs calleux, ou encore chez les enfants atteint d'un SAF. À l'île de La Réunion, il est noté une incidence élevée des SAF et les enfants porteurs de SAF représentent 10% des enfants en institutions spécialisées (Serreau et al., 2002). Il a été montré que les grands prématurés sont plus à risque de déficience cognitive globale, se manifestant par des difficultés de compréhension, de réalisation de gestes simples ou d'élaboration de jeux en rapport avec l'âge, puis de difficultés concernant les capacités d'abstraction et de mémorisation. Cette déficience cognitive peut être suspectée durant les 3 premières années à l'aide d'échelles de développement de type Brunet-Lézine en France et avec l'échelle de WISC après 6ans dont les versions évoluent avec le temps (Farooqi et al., 2016; Serenius et al., 2016; Bolk et al., 2018). Cette échelle à encore été utilisé dans l'agénésie du corps calleux, pathologie qui reste une préoccupation majeure pour lesquels le pronostic est incertain. Les résutats avaient montrée que malgré un QIT « normal » dans la majorité des patients, l'étude spécifique des 5 dimensions du WISC-V permettant de mesurer respectivement la compréhension verbale (ICV), l'indice visuo-spatial (IVS), l'indice de raisonnement fluide (IRF), la mémoire de travail (IMT) et la vitesse de traitement (IVT) pouvaient être isoléments altérés sur une seule des 5 dimensions. Le dépistage précis de la déficience permettait ensuite de mieux cibler la prise en charge du patient (Des Portes, V. Europ journal of peadiatric neurology, 2018) (des Portes et al., 2018). Une étude de Poletti en 2014 à montré que l'échelle de WISC était aussi un outil efficace dans les troubles des apprentissages des enfants, même en l'absence de déficience cognitive globabe (Poletti 2014). L'échelle WISC a encore été utilisé dans plusieurs études démontrant des désordres neurodéveloppementaux en lien avec une exposition à l'alcool en anténatal (Landgren et al., 2010;Furtado and Roriz 2016) ou a d'autre drogues tels que la cocaine (CE et al., 2006).

D'autres études ont cependant montré certaines limites du WISC dans l'évaluation clinique du fonctionnement intellectuel d'un sujet qu'il conviendra de prendre en compte lors de l'évaluation globale de l'enfant (Rozencwajg, P. 2006). L'apport du WISC paraît considérable pour une interprétation fine de la forme d'intelligence du sujet à partir, notamment, de l'appréciation de la dispersion des indices au sein du profil de ses performances. L'utilisation des données cliniques du test est sans doute à construire par les cliniciens. On peut néanmoins regretter certains choix de l'évaluation cognitive réduisant la dimension clinique du test. D'où l'idée de l'importance d'une évaluation globale du sujet. Dans bien des cas, la compréhension du fonctionnement de la personnalité de l'enfant et notamment en cas d'anxiété au moment des tests, sera à prendre en compte en utilisant d'autres épreuves comme les tests projectifs et sera nécessaire avant toute orientation de l'enfant.

Rappel des généralités sur le VABS-II

Le VABS est un outil standardisé permettant d'évaluer les comportements adaptatifs de la naissance à l'âge adulte (de Bildt et al., 2005; Sparrow et al., 2005). La seconde version révisée est en vigueur depuis 2005. Celui-ci peut être auto-administré sous forme de questionnaire ou sous forme d'entrevue semi-structurée. Une version est également disponible pour l'évaluation par un professeur afin de trianguler les observations faites dans le fonctionnement scolaire. Cette mesure développementale contient onze sous-catégories regroupées selon quatre principaux thèmes : la communication, les activités de la vie quotidienne, les habiletés d'interaction sociale et le développement moteur (Sparrow et al.,). Le parent/tuteur doit répondre selon une échelle à quatre niveaux (2 = habituellement, 1 = parfois ou partiellement, 0 = jamais, DK = je ne le sais pas). Cet outil peut être utilisé pour différentes visées, entre autres pour procéder à des évaluations diagnostiques, à des évaluations développementales, pour planifier des programmes d'intervention, pour suivre l'évolution des progrès lors d'une période d'intervention ainsi que pour contribuer à la recherche. Cette évaluation, effectuée auprès d'un large échantillon de plus de 3000 personnes, présente de bonnes propriétés métrologiques ainsi que des données normatives pour la population présentant plusieurs pathologies, entre autres, pour les personnes présentant une déficience intellectuelle quelqu'en soit la cause, un trouble de l'attention, un trouble du spectre de l'autisme

avec ou sans spécificité sur le plan du langage et autres (Goldberg et al., 2009; Selvam et al., 2016).

Rappel des généralités sur le SDQ

Depuis quelques années, dans un souci d'économie de moyens et de temps, se développent des questionnaires parentaux de type SDQ (The Strenghts and Difficulties Questionaire) (Goodman R, 1997; Goodman 2012; Goodman & Goodman 2012). Il s'agit d'un outil à la fois d'auto-évaluation par l'enfant et d'évaluation par les parents ou l'entourage proche notamment scolaire, utilisé pour évaluer le fonctionnement comportemental et l'adaptation psychosociale chez les enfants et les adolescents (Braham S, 2013) (Braham et al., 2015). Cet outil a été également utilisé pour l'évaluation des enfants exposé à l'alocool en anténatal (Alvik A, 2013;Alvik, Aalen, and Lindemann 2013; Niclasen J, 2014; Niclasen et al., 2014). Ce questionnaire a encore été utilisé récemment dans l'évaluation comportementale des enfants nés prématuréments (Rajput N, 2017;Broström, 2018; Broström et al., 2018;Rajput et al., 2018). C'est une échelle courte, de dépistage, applicable de l'âge de 3 ans jusqu'à l'âge de 17 ans. Elle est composée de 25 items répartis sur cinq sous-échelles ou : symptômes émotionnels ; problèmes de conduite ; hyperactivité/inattention ; problèmes dans les relations avec les comportements pro-sociaux. Ce qui permet d'obtenir 5 sous-scores, en plus du score total des difficultés calculé en additionnant les 4 premiers sous-scores. Chacun des 25 items est coté sur une échelle de type Likert de 3 points. Les résultats doivent être corrélés aux résultats des tests neuropsychologiques réalisés par un professionnel de santé au cours d'une journée dédié au passage de ces tests. Leur forcent résident dans le fait que ces questionnaires sont remplis par les personnes les plus proches de l'enfant, les plus à même de décrire le comportement au quotidien. Les limites se retrouvent dans la reproductibilité des tests avec la partie subjective qu'un parent peut avoir pour juger son enfant (notamment prendre en compte l'environnement psycho-familial au moment du remplissage du questionnaire).

8. DEROULEMENT DE LA RECHERCHE

8.1. CALENDRIER DE LA RECHERCHE

- Durée de la période d'inclusion : 18 mois
- Durée de participation de chaque participant : 6 mois
- Durée totale de la recherche : 24 mois

La durée totale de la recherche est fixée à 24 mois, les tests neuropsychologiques et les questionnaires comportementaux étant réalisés consécutivement pour chaque paire exposé et non-exposé, dans un ordre aléatoire et selon un délai assez proche determiné par la disponibilité au rendez-vous proposé, et ce afin que l'âge à l'évaluation soit comparable chez les exposés et les non- exposés.

Afin d'impacter au minimum la scolarité des enfants lors de cette étude, nous prévoyons d'organiser dans la mesure du possible l'ensemble des visites hors périodes scolaires (mercredis après-midi et vacances scolaires).
Nous prévoyons de débuter l'étude dès le mois d'octobre 2019 et suivant le calendrier scolaire 2019-2020 de l'académie de La Réunion.
Le bilan neuropsychologique qui dure une matinée par enfant se fera essentiellement pendant les vacances scolaires. Ainsi nous prévoyons de voir 10 enfants sur la période des vacances du 14 au 25 octobre 2019, puis 27 enfants sur la période des vacances de l'été ausctral du 19 décembre 2019 au 24 janvier 2020 puis les 5 (enfants restants sur les vacances du 9 au 20 mars 2020.
L'ensemble des seront réalisés au CHU Sud Réunion dans le pôle mère-femme enfants au niveau des consultations pour la visite pédiatrique V1 et le bilan neuropsychologique et dans le service d'ophtalmologie du CHU Sud pour la visite V2. Nous pourrons également nous déplacer au Nord de l'île si cela est necessaire.

	Inclusion V1 (M0)	Bilan Ophtalmologique Visite V2 (M3 (+/- 1mois))	Bilan Neuropsychologique Visite V3 (entre M0 et M9)
Information et Recueil du consentement éclairé	✓		
Examen Clinique [1]	✓		✓
Bilan sérologique [2]	✓		
Bilan ophtalmologique [3]		✓	
Bilan neuropsychologique [4]			✓
Auto-questionnaires parents et enseignant	✓		✓

Tableau 1. Suivi du participant
[(*)] T (unité de temps à adapter en fonction de la recherche) : M (mois)

[1] *Examen clinique : Interrogatoire détaillé : niveau scolaire, présence de reboublement/saut de classe, relevé des biais socio-familiaux avec profession des parents, niveau scolaire des autres enfants de la fratrie. Suivi éventuel spécialisé (orthophonie, psychomotricité…) déjà mis en place (oui/non). Bilan de croissance (poids, taille, PC, courbes de croissance), examen clinique complet organe par organe et examen neurologique détaillé. Recherche d'argument clinique en faveur de séquelles de l'infection au CHIKV ou de la présence de débris viraux sanctuarisé : arthralgies*

(oui/non), hallucinations visuelles ou auditives (oui/non), trouble des phanères (oui/non), recherche de lésions dyschromiques de la peau.
[2] *Bilan sérologique : sérologie chikungunya IgG (+ IgM chez les exposés)*
[3] *Bilan ophtalmologique **:** évaluation de l'acuité visuelle, de la mobilité oculaire, des champs visuels, des structures externes et le fond d'oeil*
[4] *Bilan neuropsychologique **:** WISC-V, VABS-II et SDQ*

8.2. VISITE D'INCLUSION V1 (M0)

8.2.1. Recueil du consentement

La visite de d'inclusion sera assurée par les médecins investigateurs de l'étude.

Lors de la visite d'inclusion, le médecin investigateur informe les titulaires de l'autorité parentale, répond à toutes leurs questions concernant l'objectif, la nature des contraintes, les risques prévisibles et les bénéfices attendus de la recherche. Il précise également les droits du participant dans le cadre d'une RIPH2 et vérifie les critères d'éligibilité. Si les titulaires de l'autorité parentale acceptent la particpation à l'étude, alors le mékedin investigateur informe l'enfant de l'étude et lui demande son avis.

8.3.2 Deroulement de la visite

Au cours de cette visite d'inclusion, le médecin investigateur:

- Informe les titulaires de l'autorité parentale des objectifs, de la nature des contraintes, des risques prévisibles et des bénéfices attendus de la recherche ;
- S'ils sont d'accord pour particper, le médein inestigateur informe l'enfant de l'étude (objectifs, de la nature des contraintes, des risques prévisibles et des bénéfices attendus de la recherche) dans un discours adapté à son niveau de compréhension ;
- Recueille le consentement des titulaires de l'autorité parentale **par écrit** pour participer à l'étude. Si l'enfant le souhaite, il peut également signer le consentement.
- une fois le consentement obtenu, il procède à un excamen clinique et recueille une première série d'informations via une fiche de recueil standardisée, dont les données de croissance (poids, taille, périmètre crânien, examen pédiatrique standard et neurologique détaillé).
- Une fois l'examen clinique, l'enfant est confié aux soins d'une infirmière pour prélèvement d'une sérologie IgG et IgM (10 mL maximum) pour les enfants exposés et les enfants du groupe contrôle.

Pour les enfants scolarisés, les titulaires de l'autorité parentale se voient confier à l'issue de la consultation deux auto-questionnaires SDQ, l'un pour eux, l'autre pour le professeur principal de l'enfant.

Pour les enfants non-scolarisés, seuls les titulaires de l'autorité parentale se verront proposer de répondre au questionnaire SDQ.

Cette visite est prévue pour durée d'une 1h environ.

8.3. VISITES DE SUIVI V2 (M3 (+/- 1MOIS))

BILAN OPHTALMOLOGIQUE

De même que le reste des visites, le bilan ophtalmologique sera fait dans la mesure du possible hors période scolaire, dans un délai de 3 mois suivant la première visite pour chaque enfant. Les

enfants ne pouvant être vus sur ces périodes, pourrons être programmé en semaine à titre exceptionnel.

Le bilan ophtalmologique sera réalisé en aveugle pour le statut infecté/non-infecté du sujet et comprendra une évaluation de la mobilité oculaire (recherche d'hétérophorie ou de strabisme), l'acuité visuelle, des champs visuels, des structures externes et un fond d'oeil. Il sera réalisé en ambulatoire entre la visite d'inclusion et le bilan neuropsychologique dans des cabinets sélectionnés en fonction de la zone d'habitation.

8.1. VISITE DE SUIVI V3 (M6 +/-3MOIS)

BILAN NEUROPSYCHOLOGIQUE

Le bilan neuropsychologique, programmé à l'hôpital sur une demi-journée, commencera par un examen clinique bref, assuré par un médecin investigateur afin d'éliminer les conditions aigues susceptibles d'interférer avec la réalisation des tests neuropsychologiques. Ensuite la neuropsychologue mesurera le WISC-5 (1h30 environ). Enfin, l'enfant et ses parents auront en entretien d'une durée de 1h environ afin d'aborder les difficultés rencontrées dans la vie qutidienne, les temps de jeu, la relation avec les autres (seront complété pendant cet entretien les échelles VABS-II et SDQ). Les tests seront interprétés en fonction des résultats de l'évaluation ophtalmologique et de la connaissance préalable d'un déficit auditif.

Cette visite sera programmée le mercredi ou chaque jour de la semaine en période de vacances scolaires. Cette visite est définie comme la visite de fin de participation.

Nous partons du principe que la plus part des familles créoles maitrise très bien la langue Française, mais si toutefois il y avait des problèmes de compréhension des consignes, un interprétariat créole pourra être envisagé par le personnel présent sur place.

8.6 REGLES D'ARRET DE LA PARTICIPATIONS D'UNE PERSONNE A LA RECHERCHE

- participant perdu de vue,
- retrait de consentement,
- abandon de la recherche,
- déviations majeures au protocole (aspects réglementaires, critères d'éligibilité, critère de jugement principal,…).

8.7 CONTRAINTES LIEES A LA RECHERCHE ET INDEMNISATION EVENTUELLE DES PARTICIPANTS

Les prinicpales contraintes liées à cette recherche sont :

- Accepter de réaliser l'ensemble des visites et examens prévus au protocole ;

Compte tenu de la catégorie d'âge des particpants, aucune indemnisation n'est prévue.

9. GESTION DES ÉVÉNEMENTS INDÉSIRABLES ET DES FAITS NOUVEAUX

Les évènements indésirables / effets indésirables / incidents seront à déclarer aux différents circuits de vigilances sanitaires applicables à chaque produit ou pratique concernée (vigilance du soin, pharmacovigilance, matériovigilance, hémovigilance, cosmétovigilance…) en conformité avec la règlementation en vigueur.

Les déclarants doivent spécifier que le patient est inclus dans un essai clinique et identifier précisément l'essai clinique concerné.

Si l'investigateur a connaissance d'une atteinte à la sécurité des patients dans le cadre de la recherche, il doit en informer sans délai le promoteur.

10. ASPECTS STATISTIQUES

10.1. CALCUL DE LA TAILLE D'ÉTUDE

Dans le cadre de la thèse de doctorat en médecine du Dr Raphäelle Sarton, 21 enfants de la cohorte CHIMERE (parmi 30 enfants recontactés sur les 33 évalués sur le plan neuropsychologique vers l'âge de deux ans dans CHIMERE) ont été revus en 2015, soit vers l'âge de 10 ans, afin de dépister une déficience. Ces enfants ont passé des tests d'évaluation des fonctions neurocognitives, en comparaison d'une population de référence de 626 enfants. Les résultats montraient une performance moindre des enfants infectés, inférieure à -1DS sur les fonctions verbales et non verbales avec une performance équivalente sur les fonctions d'apprentissage de l'EDA (Évaluation des fonctions cognitives et Apprentissages de l'enfant), argument à l'appui d'une hypothèse unilatérale. Une moindre performance de –1DS entre le groupe « infectés » et le groupe « non infectés » nous parait donc une cible raisonnable à viser sur le QIT du WISC-V.

Si, compte tenu d'une attrition limitée à un tiers (prévision raisonnable compte tenu de la dernière évaluation de 2015), nous parvenons à reconvoquer ces 21 enfants infectés (ou à en reconvoquer 21 parmi les 30 pour lesquels le contact a été maintenu) et autant d'enfants d'un groupe non infectés (pas de pathologie connue, QIT moyen attendu de 100), il faudrait, pour mettre en évidence une différence cliniquement pertinente de QIT de 15 points (1 DS) entre deux groupes appariés, avec une deviation standard de cette différence de 30 points (2DS), au risque alpha de 5%, 42 enfants pour obtenir une puissance statistique de 93% en hypothèse unilatérale confirmatoire (différence entre les moyennes des deux groupes > 0) et de 88% en hypothèse bilatérale sans a priori (différence entre les moyennes des deux groupes ≠ 0).

Les deux simulations de puissance obtenue avec 42 enfants pour une difference observée comprise entre 5 et 15 points, une deviation standard double de la difference observée, au risque alpha de 5%, pour une distribution du QIT présumée normale des enfants réunionnais, sont présentées en annexe, en hypothèse unilatérale (**Annexe 1A**) puis en hypothèse bilatérale (**Annexe 1B**) avec pour éventualité principale, le scénario énonce plus haut sur-ligné en jaune. Une simulation semblable sans connaissance de la nature de la distribution conférerait des puissances très proches de 94% et 90%.

Ainsi, avec une attrition prévisible d'un tiers par rapport aux effectifs observés dans la cohorte CHIMERE dix ans auparavant nous pourrons compter dans le groupe exposé 7 cas d'encéphalite/encéphalopathie, 14 cas de prostration (forme non encéphalitique), soit un nombre total de 21 enfants à évaluer sur le plan ophtalmique, neuropsychologique et comportemental

Ces effectifs permettront également de détecter au risque alpha de 5%, avec une puissance comprise entre 26% et 65%, une différence cliniquement pertinente attendue de 15 points de QIT (moyenne attendue de 90 pour le groupe prostration, 75 pour le groupe encéphalite, avec une deviation standard variant entre 15 et 30 points dans chaque groupe sur l'échelle WISC-V).

Dans les mêmes conditions, d'autres différences cliniquement pertinentes sur plusieurs critères de jugement secondaires seront observées. Quelques exemples pour les 4 indices du WISC-V et ses 8 subtests, pour les questionnaires SDQ, la note T du VABS-II, ou la différence des redoublants vous en sont donnés à tire indicatif dans le Tableau II ci dessous.

Tableau II. Puissances obtenues pour un risque α de 5%, une deviation standard variant du simple au double de l'écart type présumé avec 7 cas d'encéphalites/encéphalopathies et 14 cas de prostrations en hypothèse unilatérale (performance meilleure en cas de prostration)

<table>
<tr><td></td><td colspan="3">Indices WISC-V</td><td colspan="4">Subtests du WISC-V</td></tr>
<tr><td></td><td>QIT</td><td colspan="2">ICV, IVS, IRF, IMT, IVT</td><td colspan="2">SIM, VOC, COM</td><td>CUB, IDC, MAT</td><td>MCH, SLC</td></tr>
<tr><td>Encéphalite</td><td>75</td><td colspan="2">75</td><td colspan="2">5</td><td>5</td><td>5</td></tr>
<tr><td>Prostration</td><td>90</td><td colspan="2">90</td><td colspan="2">10</td><td>9</td><td>9</td></tr>
<tr><td>Ecart type</td><td>15</td><td colspan="2">15</td><td colspan="2">3</td><td>3</td><td>3</td></tr>
<tr><td>Puissance</td><td>26-65%</td><td colspan="2">26-65%</td><td colspan="2">52-96%</td><td>38-85%</td><td>38-85%</td></tr>
<tr><td></td><td colspan="5">Questionnaire SDQ</td><td>VABS-II</td><td>Scolarité</td></tr>
<tr><td></td><td>Difficultés totales</td><td>THADA</td><td>Problèmes de conduite</td><td>Symptomes émotionnels</td><td>Comportement antisocial</td><td>Note T</td><td>% de Redoublants*</td></tr>
<tr><td>Encéphalite</td><td>20</td><td>6</td><td>8</td><td>8</td><td>6</td><td>70</td><td>30</td></tr>
<tr><td>Prostration</td><td>30</td><td>8</td><td>10</td><td>9</td><td>9</td><td>85</td><td>5</td></tr>
<tr><td>Ecart type</td><td>5</td><td>1</td><td>2</td><td>1</td><td>2</td><td>15</td><td>-</td></tr>
<tr><td>Puissance</td><td>65-99%</td><td>65-99%</td><td>26-65%</td><td>26-65%</td><td>45-92%</td><td>26-65%</td><td>39%</td></tr>
</table>

Simulations effectuées à partir de tests T de Student, sauf * par test de Fisher exact

10.2. MÉTHODES STATISTIQUES EMPLOYÉES

10.2.1 PLAN D'ANALYSE

Un plan d'analyse détaillé sera établi et fera l'objet d'une validation par le conseil scientifique de l'étude. Ses modifications ultérieures devront intervenir avant levée d'insu sur la base de données et seront validées par le conseil scientifique. Toutes ces analyses seront réalisées au CIC-EC de La Réunion, sur le logiciel Stata 14. Les tests statistiques seront appliqués avec une formulation bilatérale ou unilatérale le cas échéant (dans les analyses de sous groups notamment pour différencier encéphalites/encéphalopathies et prostrations) et un risque α de 5 %. Dans ce cas, une méthode corrective (Bonferroni, Duncan) sera appliquée au besoin pour prendre en compte la multiplicité des tests effectués et maintenir le risque d'erreur de première espèce à 5 %.

10.2.2 CRITERES DE JUGEMENT QUANTITATIFS

Analyse univariée: la distribution de chaque score (moyenne, écart-type, quartiles) sera décrite dans chaque groupe d'exposition (exposés et non exposés) et pour chaque forme clinique de l'infection (encéphalite/ prostration).

Analyse bivariée : comparaison des moyennes de chaque critère de jugement continu selon les groupes d'expostion (exposé versus non exposé) tenant compte de l'appariement par des tests des rangs signés de Wilcoxon; puis au sein du groupe exposé pour chaque type d'infections (encéphalite/ prostration) par des tests de non paramétriques de Mann-Whitney, vue la faiblesse des effectisfs.

Le test alpha de Cronbach sera utilisé pour déterminer la cohérence interne des échelles du WISC-5 et des autres différents scores. Des tests de corrélation seront faits au besoin entre les différentes échelles.

10.2.3 CRITERES DE JUGEMENT QUALITATIFS

Analyse bivariée : comparaison des proportions de chaque critère de jugement catégoriel selon les groupes d'expostion (exposé versus non exposé) tenant compte de l'appariement par des

tests de Mac Nemar; puis au sein du groupe exposé pour chaque type d'infections (encéphalite/ prostration) par des tests de non paramétriques de Fisher Exact, vue la faiblesse des effectisfs.

Les enfants du groupe non exposé qui auront une sérologie positive en raison d'une infection postnatale et leur paire seront exclus de l'analyse, toute infection à chikungunya pouvant se compliquer d'une atteinte neurologique susceptible de perturber le neurodéveloppement, notamment quand l'infection est survenue dans les 6 premiers mois, celle-ci pouvant se compliquer d'encéphalite ou d'encéphalopathie (Gérardin P et al, Neurology 2016).

Des interactions avec les facteurs socio-familiaux seront recherchées pour voir si ces facteurs modifient l'effet de l'exposition sur le neurodéveloppement. Des analyses stratifiées sur le facteur socio-familial modificateur d'effet pourront être réalisées.

Un plan d'analyse détaillé sera défini et fera l'objet d'une validation par le Conseil Scientifique de l'étude. Les modifications ultérieures devront intervenir avant la levée d'insu sur la base de données et seront systématiquement validées par le Conseil Scientifique.

11. SURVEILLANCE DE LA RECHERCHE

11.1 CONSEIL SCIENTIFIQUE

11.1.1 Composition

Ce conseil sera composé de tous les médecins investigateurs, de médecins cliniciens, de chercheurs expérimentés dans le domaine des maladies infectieuses et émergentes.

- Investigateur principal: Dr Raphaëlle Sarton
- Investigateurs associés : Dr Brahim Boumahni
- Neuropsychologues du CAMSP
- Pédiatres invités :
- Méthodologiste : Dr Patrick Gérardin
- Représentant du promoteur (Chef de projet DRCI).

11.1.2 Rythme des réunions

Le conseil scientifique se réunira systématiquement au démarrage, puis au moins une fois par trimestre, jusqu'à la fin de l'étude (cf. calendrier de l'étude **8.1**).

11.1.3 Rôle du conseil scientifique

Le conseil scientifique aura pour mission de superviser la recherche, c'est à dire:

- Prendre toute décision importante à la demande de l'investigateur principal concernant la bonne marche de la recherche et le respect du protocole,
- Vérifier le respect de l'éthique,
- Informer le Centre de Méthodologie et de Gestion des données et les partenaires scientifiques invités de l'état d'avancement de la recherche, des problèmes éventuels, des résultats déjà disponibles.
- Décider de toute modification pertinente du protocole nécessaire à la poursuite de la recherche, notamment :
  - Des mesures facilitant le recrutement dans la recherche (inclusions),
  - Les amendements au protocole avant leur présentation au CPP,
  - L'éventualité d'une analyse intermédiaire,
  - La discussion des résultats et la stratégie de publications.

Le Conseil Scientifique pourra proposer de prolonger ou d'interrompre la recherche en cas de rythme d'inclusion trop lent, d'un trop grand nombre de perdus de vue (évaluation incomplète), de violations majeures du protocole ou bien pour des raisons médicales ou administratives.

A l'issu des réunions, l'investigateur principal informera le promoteur des décisions arrêtées. Les décisions concernant un amendement majeur ou une modification de budget devront être approuvées par le promoteur.

12. DROITS D'ACCES AUX DONNEES ET DOCUMENTS SOURCE

12.1. ACCÈS AUX DONNÉES

L'acceptation de la participation au protocole implique que les investigateurs mettront à disposition les documents et données individuelles strictement nécessaires au suivi, au contrôle de qualité et à l'audit de la recherche, à la disposition des personnes ayant un accès à ces documents conformément aux dispositions législatives et réglementaires en vigueur.

12.2. DONNÉES SOURCE

Ensemble des informations figurant dans des documents originaux, ou dans des copies authentifiées de ces documents, relatif aux examens cliniques, aux observations ou à d'autres activités menées dans le cadre d'une recherche et nécessaires à la reconstitution et à l'évaluation de la recherche. Les documents dans lesquels les données sources sont enregistrées sont appelés les documents sources.

12.3. CONFIDENTIALITÉ DES DONNÉES

Conformément aux dispositions législatives en vigueur, les personnes ayant un accès direct aux données source prendront toutes les précautions nécessaires en vue d'assurer la confidentialité des informations relatives aux médicaments expérimentaux, aux recherches, aux personnes qui s'y prêtent et notamment en ce qui concerne leur identité ainsi qu'aux résultats obtenus. Ces personnes, au même titre que les investigateurs eux-mêmes, sont soumises au secret professionnel.

Pendant la recherche ou à son issue, les données recueillies sur les personnes qui s'y prêtent et transmises au promoteur par les investigateurs (ou tous autres intervenants spécialisés) seront rendues anonymes. Elles ne doivent en aucun cas faire apparaître en clair les noms des personnes concernées ni leur adresse.

Le promoteur s'assurera que chaque personne qui se prête à la recherche a donné son accord par écrit pour l'accès aux données individuelles la concernant et strictement nécessaires au contrôle de qualité de la recherche.

13. CONTRÔLE ET ASSURANCE QUALITÉ

13.1. CONSIGNES POUR LE RECUEIL DES DONNEES

Toutes les informations requises par le protocole doivent être consignées sur les cahiers d'observation et une explication doit être apportée pour chaque donnée manquante. Les données doivent être recueillies au fur et à mesure qu'elles sont obtenues, et transcrites dans ces cahiers de façon nette et lisible.
Les données seront recueillies sur cahier d'observation électronique.

13.2. CONTRÔLE QUALITÉ

Un attaché de recherche clinique mandaté par le promoteur visite de façon régulière chaque centre investigateur, lors de la mise en place de la recherche, une ou plusieurs fois en cours de recherche selon le rythme des inclusions et en fin de recherche. Lors de ces visites, et conformément au plan de monitorage basé sur le risque (participant, logistique, impact, ressources), les éléments suivants seront revus :

- consentement éclairé,
- respect du protocole de la recherche et des procédures qui y sont définies,

Toute visite fera l'objet d'un rapport de monitorage par compte-rendu écrit.

13.3. GESTION DES DONNÉES

Les données seront recueillies sur un cahier d'observation papier par l'investigateur principal et les investigateurs associés, l'ophtalmologiste et la neuropsychologue.
La saisie informatique sera assurée de façon hebdomadaire par les investigateurs en simple saisie sur un masque de saisie numérisé Clinsight® avec des contrôles à la saisie, et contrôlée par le méthologiste en charge du projet.
Le masque de saisie Clinsight® sera réalisé par le (la) data-manager du CIC 1410 du CHU de la Réunion L'accès à la base de données sera sécurisé (accès par identifiant et mot de passe personnels).
Un contrôle de qualité de la saisie sera effectué sur 20% des dossiers tirés au sort.
Si le contrôle s'avère de mauvaise qualité, une double saisie sera alors effectuée.
Le processus de gel/dégel des données sera réalisé conformément à la procédure mise en place au CIC 1410.

13.4. AUDIT ET INSPECTION

Un audit peut être réalisé à tout moment par des personnes mandatées par le promoteur et indépendantes des personnes menant la recherche. Il a pour objectif de vérifier la sécurité des participants et le respect de leurs droits, le respect de la réglementation applicable et la fiabilité des données.

Une inspection peut également être diligentée par une autorité compétente (ANSM pour la France ou EMA dans le cadre d'un essai européen par exemple).

L'audit, aussi bien que l'inspection, pourront s'appliquer à tous les stades de la recherche, du développement du protocole à la publication des résultats et au classement des données utilisées ou produites dans le cadre de la recherche.

Les investigateurs acceptent de se conformer aux exigences du promoteur en ce qui concerne un audit et à l'autorité compétente pour une inspection de la recherche.

14. CONSIDÉRATIONS ÉTHIQUES ET RÉGLEMENTAIRES

Le promoteur et l'(es) investigateur(s) s'engagent à ce que cette recherche soit réalisée en conformité avec la loi n°2012-300 du 5 mars 2012 relative aux recherches impliquant la personne humaine, ainsi qu'en accord avec les Bonnes Pratiques Cliniques (I.C.H. version 4 du 9 novembre 2016 et décision du 24 novembre 2006) et la déclaration d'Helsinki (qui peut être retrouvée dans sa version intégrale sur le site http://www.wma.net).

La recherche est conduite conformément au présent protocole. Hormis dans les situations d'urgence nécessitant la mise en place d'actes thérapeutiques précis, l'(es) investigateur(s) s'engage(nt) à respecter le protocole en tous points en particulier en ce qui concerne le recueil du consentement et la notification et le suivi des événements indésirables graves.

Cette recherche a reçu l'avis favorable du Comité de Protection des Personnes (CPP) de *nom du CPP*.

Le CHU de La Réunion, promoteur de cette recherche, souscrira un contrat d'assurance en responsabilité civile auprès de la SHAM conformément aux dispositions du code de la santé publique.

Les données enregistrées à l'occasion de cette recherche font l'objet d'un traitement informatisé à du CHU de La Réunion dans le respect de la loi n°78-17 du 6 janvier 1978 relative à l'informatique, aux fichiers et aux libertés modifiée par la 2018-493 du 20 juin 2018.

Cette recherche entre dans le cadre de la « Méthodologie de référence » (MR-001) en application des dispositions de l'article 54 alinéa 5 de la loi du 6 janvier 1978 modifiée relative à l'information, aux fichiers et aux libertés. Ce changement a été homologué par décision du 5 janvier 2006, mise à jour le 21 juillet 2016. Le CHU de La Réunion a signé un engagement de conformité à cette « Méthodologie de référence ».

- Cette recherche sera enregistrée sur le site http://clinicaltrials.gov/

MODIFICATIONS AU PROTOCOLE

Toute modification substantielle, c'est à dire toute modification de nature à avoir un impact significatif sur la protection des personnes, sur les conditions de validité et sur les résultats de la recherche, sur la qualité et la sécurité des produits expérimentés, sur l'interprétation des documents scientifiques qui viennent appuyer le déroulement de la recherche ou sur les modalités de conduite de celle-ci, fait l'objet d'un amendement écrit qui est soumis au promoteur ; celui-ci doit obtenir, préalablement à sa mise en œuvre, un avis favorable du CPP.

Les modifications non substantielles, c'est à dire celles n'ayant pas d'impact significatif sur quelque aspect de la recherche que ce soit, sont communiquées au CPP à titre d'information.

Toutes les modifications sont validées par le promoteur, et par tous les intervenants de la recherche concernés par la modification, avant soumission au CPP. Cette validation peut nécessiter la réunion de tout comité constitué pour la recherche. .

Toutes les modifications au protocole doivent être portées à la connaissance de tous les investigateurs qui participent à la recherche. Les investigateurs s'engagent à en respecter le contenu.

Toute modification qui modifie la prise en charge des participants ou les bénéfices, risques et contraintes de la recherche fait l'objet d'une nouvelle note d'information et d'un nouveau formulaire de consentement dont le recueil suit la même procédure que celle précitée.

15. CONSERVATION DES DOCUMENTS ET DES DONNEES RELATIFS A LA RECHERCHE

Les documents suivants relatifs à cette recherche sont archivés par l'investigateur conformément aux Bonnes Pratiques Cliniques :

- pour une durée de 15 ans suivant la fin de la recherche *(recherches portant sur des médicaments, des dispositifs médicaux ou des dispositifs médicaux de diagnostic in vitro ou recherches ne portant pas sur un produit mentionné à l'article L.5311-1 du code de la santé publique),*

- Le protocole et les modifications éventuelles au protocole
- Les cahiers d'observation (copies)
- Les dossiers source des participants ayant signé un consentement
- Tous les autres documents et courriers relatifs à la recherche

- pour une durée de 30 ans suivant la fin de la recherche

- L'exemplaire original des consentements éclairés signés des participants

Tous ces documents sont sous la responsabilité de l'investigateur pendant la durée réglementaire d'archivage.
Aucun déplacement ou destruction ne pourra être effectué sans l'accord du promoteur. Au terme de la durée réglementaire d'archivage, le promoteur sera consulté pour destruction. Toutes les données, tous les documents et rapports pourront faire l'objet d'audit ou d'inspection.

16. RAPPORT FINAL

Dans un délai d'un an suivant la fin de la recherche ou son interruption, un rapport final sera établi et signé par le promoteur et l'investigateur. Ce rapport sera tenu à la disposition de l'autorité compétente. Le promoteur transmettra au CPP et, le cas échéant, à l'ANSM les résultats de la recherche sous forme d'un résumé du rapport final dans un délai d'un an après la fin de la recherche.

17. REGLES RELATIVES À LA PUBLICATION

17.1. COMMUNICATIONS SCIENTIFIQUES

L'analyse des données fournies par les centres investigateurs est réalisée par le CIC 1410 du CHU de La Réunion. Cette analyse donne lieu à un rapport écrit qui est soumis au promoteur, qui transmettra au Comité de Protection des Personnes et à l'autorité compétente.

Toute communication écrite ou orale des résultats de la recherche doit recevoir l'accord préalable de l'investigateur coordonnateur et, le cas échéant, de tout comité constitué pour la recherche.

L'investigateur coordonnateur/principal s'engage à mettre à disposition du public les résultats de la recherche aussi bien négatifs et non concluants que positifs.

La publication des résultats principaux mentionne le nom du promoteur, de tous les investigateurs ayant inclus ou suivi des participants dans la recherche, des méthodologistes, biostatisticiens et data managers ayant participé à la recherche, des vigilants ayant participé à l'analyse de la sécurité des participants, des membres du (des) comité(s) constitué(s) pour la recherche la source de financement. Il sera tenu compte des règles internationales d'écriture et de publication (*The Uniform Requirements for Manuscripts* de l'ICMJE, avril 2010).

17.2. COMMUNICATION DES RÉSULTATS AUX PARTICIPANTS

Conformément à la loi n°2002-303 du 4 mars 2002, les participants sont informés, à leur demande, des résultats globaux de la recherche.

17.3. CESSION DES DONNÉES

La gestion des données est assurée par le CIC 1410 du CHU de La Réunion. Les conditions de cession de tout ou partie de la base de données de la recherche sont décidées par le promoteur de la recherche et font l'objet d'un contrat écrit.

REFERENCES BIBLIOGRAPHIQUES

Alvik A, Aalen OO, Lindemann R. 2013. “Early Fetal Binge Alcohol Exposure Predicts High Behavioral Symptom Scores in 5.5-Year-Old Children.” *Alcoholism: Clinical and Experimental Research* 37 (11): 1954–62. doi:10.1111/acer.12182.

Aubry P. 2012. “Arboviroses Tropicales.” 1–14. http://medecinetropicale.free.fr/cours/arboviroses.pdf

Bachelier D, Cogne G. 2017. “Le bilan avec le WISC-V et ses outiles complémentaires: Guide pratique pour l'évaluation.” Ed: Dunod, Paris. 224 pp.

Bale Jr JF. 2012 “Cytomegalovirus infections.” *Seminars in Pediatric Neurology* 19(3):101-106. doi: 10.1016/j.spen.2012.02.008.

Bolk J, Farooqi A, Hafström M, Åden U, Serenius F. 2018. “Developmental Coordination Disorder and Its Association With Developmental Comorbidities at 6.5 Years in Apparently Healthy Children Born Extremely Preterm.” *JAMA Pediatrics* 1–10. doi:10.1001/jamapediatrics.2018.1394.

Braham S, Kacem IH, Mlika S, Moalla Y, Walha A, Gaddour N, Ayadi H, Ghribi F. 2015. “Impulsivité et Problèmes d’Internalisation et d’Externalisation Chez l’Adolescent.” *Encephale* 41 (3): 215–220. doi:10.1016/j.encep.2014.03.003.

Broström L, Vollmer B, Bolk J, Eklöf E, Ådén U. 2018. “Minor Neurological Dysfunction and Associations with Motor Function, General Cognitive Abilities, and Behaviour in Children Born Extremely Preterm.” *Developmental Medicine & Child Neurology*, no. 1: 1–7. doi:10.1111/dmcn.13738.

Boumahni B, Bintner M. 2012. Devenir à 5ans des infections materno-foetales à chikungunya. "*Médecine Tropicale*" Vol 72 spécial Chikungunya 94–96.

Calba C, Guerbois-Galla M, Franke F, Jeannin C, Auzet-Caillaud M, Grard G, Pigaglio L, Deccoppet A, Weicherfing J, Savaill MC, Munoz-Rivieiro M, Chaud P, Cadiou B, Ramalli L, Fournier P, Noël H, de Lamballerie X, Paty MC, Leparc-Goffart I. 2017 “Preliminary report of an autochtonous chikungunya outbreak in France, July to September 2017.” *Eurosurveillance* 22(39):17-00647. doi:10.2807/1560-7917.ES.2017.22.39.17-00647

Contopoulos-Ioannidis D, Newman-Lindsay S, Chow C, LaBeaud AD. 2018. “Mother-to-child transmission of Chikungunya virus: a systematic review and meta-analysis.” 10.1371/journal.pntd.0006510.” *PLoS Neglected Tropical Diseases* 12 (6): 1–20. doi:10.1371/journal.pntd.0006510.

Couderc T, Chrétien F, Schilte C, Disson O, Madly B, Guivel-Benhassine F, Touret Y, Barau G, Cayet N, Schuffenecker I, Desprès P, Arenzana-Seisdedos F, Michault A, Albert ML, Lecuit M. 2008. "A mouse model for Chikungunya: young age and inefficient type-1 interferon signaling are risk factors for severe disease" *PLoS Pathogens* 4(2): e29. doi:10.1371/journal.ppat.0040029

Couderc T, Khandoudi N, Grandadam M, Visse C, Gangneux N, Bagot S, Prost JF, Lecuit M. 2009. “Prophylaxis and Therapy for Chikungunya Virus Infection.” *The Journal of Infectious Diseases* 200 (4): 516–23. doi:10.1086/600381.

Das T, Jaffar-Bandjee MC, Hoarau JJ, Krejbich-Troto P, M Denizot M, Li-Pat-Yuen G, Sahoo R, Guiraud P, Ramful D, Robin S, Alessandri JL, Gaüzère BA, Gasque P. 2010. "Chikungunya fever: CNS infection and pathogenesis of a re-emerging arbovirus." *Progress in Neurobiology* 91(2): 121-129.

Das T, Hoarau JJ, Jaffar-Bandjee MC, Maquart M, Gasque P. 2015. "Multifaceted innate immune responses engaged by astrocytes, microglia and resident dendritic cells against chikungunya neuroinfection." *Journal of General Virology* 96(Pt 2): 294-310.

de Bildt A, Kraijer SD, Sparrow S, Minderaa R. 2005. “Adaptive Functioning and Behaviour Problems in Relation to Level of Education in Children and Adolescents with Intellectual Disability.” *Journal of Intellectual Disability Research* 49 (9): 672–681. doi:10.1111/j.1365-2788.2005.00711.x

des Portes V, Rolland A, Velazquez-Dominguez J, Peyric E, Cordier MP, Gaucherand P, Massardier J, Massoud M, Curie A, Pellot AS, Rivier R, Lacalm A, Clement A, Ville D, Guibaud L. 2018. “Outcome of Isolated Agenesis of the Corpus Callosum: A Population-Based Prospective Study.” *European Journal of Paediatric Neurology* 22 (1): 82–92. doi:10.1016/j.ejpn.2017.08.003.

Economopoulou A, Dominguez M, Helynck B, Sissoko D, Wichmann O, Quenel P, Germonneau P, Quatresous I. 2009. “Atypical Chikungunya Virus Infections: Clinical Manifestations, Mortality and Risk Factors for Severe Disease during the 2005-2006 Outbreak on Réunion.” *Epidemiology and Infection* 137 (4): 534–541. doi:10.1017/S0950268808001167.

Escobar M, Nieto AJ, Loaiza-Osorio S, Barona JS, Rosso F. 2017. “Pregnant Women Hospitalized with Chikungunya Virus Infection, Colombia, 2015.” *Emerging Infectious Diseases* 23 (11): 1777–1783. doi:10.3201/eid2311.170480.

Farooqi A, Adamsson M, Serenius F, Hãgglöf B. 2016. “Executive Functioning and Learning Skills of Adolescent Children Born at Fewer than 26 Weeks of Gestation.” *PLoS ONE* 11 (3): 1–20. doi:10.1371/journal.pone.0151819.

Fritel X, Rollot O, Gérardin P, Gaüzère BA, Bideault J, Lagarde L, Dhuime B, Orvain E, Cuillier F, Ramful D, Sampériz S, Jaffar-Bandjee MC, Michault A, Cotte L, Kaminski M, Fourmaintraux A, the Chikungunya-Mère-Enfant team. 2010. "Chikungunya virus infection during pregnancy, Reunion, France, 2006." *Emerging Infectious Diseases* 16(3): 418-425. doi: 10.3201/eid1603.091403.

Furtado EF, Roriz ST. 2016. “Inattention and Impulsivity Associated with Prenatal Alcohol Exposure in a Prospective Cohort Study with 11-Years-Old Brazilian Children.” *European Child and Adolescent Psychiatry* 25 (12). Springer Berlin Heidelberg: 1327–1335. doi:10.1007/s00787-016-0857-y.

Gérardin P, Guernier V, Perrau J, Fianu A, Le Roux K, Grivard P, Michault A, de Lamballerie X, Flahault A, Favier F. 2008. “Estimating Chikungunya Prevalence in La Réunion Island Outbreak by Serosurveys: Two Methods for Two Critical Times of the Epidemic.” *BMC Infectious Diseases* 8 (1): 99. doi:10.1186/1471-2334-8-99.

Gérardin P, Barau G, Michault A, Bintner M, Randrianaivo H, Choker G, Lenglet Y, Touret Y, Bouveret A, Grivard P, Le Roux K, Blanc S, Schuffenecker I, Couderc T, Arenzana-Seisdedos F, Lecuit M, Robillard PY. 2008. “Multidisciplinary Prospective Study of Mother-to-Child Chikungunya Virus Infections on the Island of La Reunion.” 5 (3):e60. doi: 10.1371/journal.pmed.0050060.

Gérardin P, Fianu A, Malvy D, Mussard C, Boussaïd K, Rollot O, Michault A, Gaüzere BA, Bréart G, Favier F. 2011. “Perceived Morbidity and Community Burden after a Chikungunya Outbreak: The TELECHIK Survey, a Population-Based Cohort Study.” *BMC Medicine* 9 (1): 1–11. doi:10.1186/1741-7015-9-5.

Gérardin P, Sampériz S, Ramful D, Boumahni B, Bintner M, Alessandri JL, Carbonnier M, Tiran-Rajaoefera I, Beullier G, Boya I, Noormahomed T, Okoi J, Rollot O, Cotte L, Jaffar-Bandjee MC, Michault A, Favier F, Kaminski M, Fourmaintraux A, Fritel X. 2014. “Neurocognitive Outcome of Children Exposed to Perinatal Mother-to-Child Chikungunya Virus Infection: The CHIMERE Cohort Study on Reunion Island.” Edited by Ann M. Powers. *PLoS Neglected Tropical Diseases* 8 (7): e2996. doi:10.1371/journal.pntd.0002996.

Gérardin P, Couderc T, Bintner M, Tournebize P, Renouil M, Lémant J, Boisson V, Borgherini G, Staikowsky F, Shramm F, Lecuit M, Michault A; Encephalchik Study group. 2016 "Chikungunya virus-associated encephalitis: a cohort study on La Réunion island." *Neurology* 86(1): 94-102. doi: 10.1212/WNL.0000000000002234.

Goldberg MR, Dill CA, Shin JY, Nguyen VN. 2009. “Reliability and Validity of the Vietnamese Vineland Adaptive Behavior Scales with Preschool-Age Children.” *Research in Developmental Disabilities* 30 (3): 592–602. doi:10.1016/j.ridd.2008.09.001.

Goodman A, Goodman R. 2012. “Strengths and Difficulties Questionnaire Scores and Mental Health in Looked after Children.” *British Journal of Psychiatry* 200 (5): 426–427. doi:10.1192/bjp.bp.111.104380.

Hoarau JJ, Jaffar-Bandjee MC, Krejbich-Trotot P, Das T, Li-Pat-Yuen G, Dassa B, Denizot M, Guichard E, Ribera A, Henni T, Tallet F, Moiton MP, Gauzère BA, Bruniquet S, Jaffar-Bandjee Z, Morbidelli P, Martigny G, Jolivet M, Gay F, Grandadam M, Tolou H, Vieillard V, Debré P, Autran B, Gasque P. 2010. "Persistent chronic inflammation and infection by chikungunya arthritogenic alphavirus in spite of a robust host immune response" *Journal of Immunology* 184(10): 5914-527. doi: 10.4049/jimmunol.0900255.

Lamblin D, Maillard T, Provost C, Ricquebourg M. 2008. “Table Ronde Prévention de l’Ensemble Des Troubles Causés Par l’Alcoolisation Fœtale à La Réunion [Fetal Alcohol Spectrum Disorder Prevention in Reunion Island.]” *Archives de Pédiatrie* 15(5): 513–515. doi: 10.1016/S0929-693X(08)71816-5.

Landgren M, Svensson L, Stromland K, Andersson-Gronlund M. 2010. “Prenatal Alcohol Exposure and Neurodevelopmental Disorders in Children Adopted From Eastern Europe.” *Pediatrics* 125 (5): e1178–1185. doi:10.1542/peds.2009-0712.

Lenglet Y, Barau G, Robillard PY, Randrianaivo H, Michault A, Bouveret A, Gérardin P, Boumahni B,

Touret Y, Kauffmann E, Schuffenecker I, Gabriele M, Fourmaintraux A. 2006. "Infection À Chikungunya Chez La Femme Enceinte et Risque de Transmission Materno-Fœtale. [Chikungunya infection in pregnancy: Evidence for intrauterine infection in pregnant women and vertical transmission in the parturient. Survey of the Reunion Island outbreak]." *Journal de Gynécolologie, Obstétrique et Bioliogie de la Reproduction (Paris)* 35 (6): 578–583.

Lim SM, van den Ham HJ, Oduber M, Martina E, Zaaraoui-Boutahar F, Roose JM, van Ijcken WFJ, Osterhaus A, Andeweg AC, Koroka P, Martina B. 2017. "Transcriptomic analyses reveal differential gene expression of immune and cell death pathways in the brains of mice infected with West Nile virus and Chikungunya virus." *Frontiers in Microbiology* 17: 8: 1556. doi: 10.3389/fmicb.2017.01556.

Malvy D, Ezzedine K, Mamani-Matsuda M, Autran B, Tolou H, Receveur MC, Pistone T, Rambert J, Moynet D, Mossalayi D. 2008." Destructive arthritis in a patient with chikungunya virus infection and persistent specific IgM antibodies." *BMC Infectous Diseases* 9: 200. doi: 10.1186/1471-2334-9-200.

Mehta R, Gérardin P, de Brito CAA, Soares CN, Ferreira MLB, Solomon T. 2018 "The neurological complications of chikungunya virus: a systematic review." *Review of Medical Virology* 28(3): e1978. doi: 10.1002/rmv.1978.

Miner JJ, Daniels BP, Shreta B, Proenca-Modena JL, Lew ED, Lazear HM, Gorman MJ, Lemke G, Klein RS, Diamond MS. "The TAM receptor Mertk protects against neuroinvasice viral infection by maintaining blood-brain barrier." 2015 *Nature Medicine* 21(12):1464-1472. doi: 10.1038/nm.3974.

Morrow CE, Culbertson JL, Accornero VH, L Xue, Anthony JC, and Bandstra ES. 2006. "Learning Disabilities and Intellectual Functioning in School-Aged Children with Prenatal Cocaine Exposure." *Developmental Neuropsychology* 30 (3): 905–931. http://search.ebscohost.com/login.aspx?direct=true&db=cin20&AN=106166929&site=ehost-live.

Nakagawa K, Kadoya M, Matsumoto H, Moriuchi H, Ikewaki K, Kaida K. 2018. ["A 15-year-old girl with congenital cytomegalovirus infection presenting with sensorineural hearing impairment and cerebral white matter lesions but no intellectual disability."]. Rinsho Shinkeigaku 58(5):320-323. doi: 10.5692/clinicalneurol.cn-001164.

Niclasen J, Nybo-Andersen AM, Teasdale TW, Strandberg-Larsen K. 2014. "Prenatal Exposure to Alcohol, and Gender Differences on Child Mental Health at Age Seven Years." *Journal of Epidemiology and Community Health* 68 (3): 224–232. doi:10.1136/jech-2013-202956.

Platt DJ, Smith AM, Arora N, Diamond MS, Coyne CB, Miner JJ. 2018. "Zika virus-related neurotropic flaviruses infect human placental explants and cause fetal demise." *Science and Translational Medicine* 10(426): eaao7090. doi: 10.1126/scitranslmed.aao7090.

Pereira L, Petit M, Fong A, Tsuge M, Tabata T, Fang-Hoover K, Maidji E, Zydek M, Zhou Y, Inoue N, Loghavi S, Pepkowitz S, Kauvar LM, Ogunuemy D. 2014. "Intrauterine growth restriction caused by underlying congenital cytomegalovirus infection." *Journal of Infectious Diseases* 209(10):1573-1584. doi: 10.1093/infdis/jiu019.

Poletti M. 2014. "WISC-IV Intellectual Profiles in Italian Children With Specific Learning Disorder and Related Impairments in Reading, Written Expression, and Mathematics." *Journal of Learning Disabilities* 49 (3): 320–335. doi:10.1177/0022219414555416.

Powell JR, Tabachnick WJ. 2013. "History of Domestication and Spread of Aedes Aegypti--a Review." *Memórias Do Instituto Oswaldo Cruz* 108 (August): 11–17. doi:10.1590/0074-0276130395.

Rajput N, McKinlay C, Purdie G, Filipovska J, Battin M, Patel H, Tuohy P. 2018. "Community-Based Screening to Detect School Readiness Problems in Very Preterm Children." *Journal of Paediatrics and Child Health* 54 (3): 238–246. doi:10.1111/jpc.13707.

Ramful D, Carbonnier M, Pasquet M, Bouhmani B, Ghazouani J, Noormahomed T, Beullier G, Attali T, Sampériz S, Fourmaintraux A, Alessandri JL. 2007. "Mother-to-Child Transmission of Chikungunya Virus Infection." *The Pediatric Infectious Disease Journal* 26 (9): 811–815. doi:10.1097/INF.0b013e3180616d4f.

Renault P, Solet JL, Sissoko D, Balleydier E, Larrieu S, Filleul L, Lassalle C, Thiria J, Rachou E, de Valk H, Ilef D, Ledrans M, Quatresous I, Quenel P, Pierre V. 2007. "A Major Epidemic of Chikungunya Virus Infection on Réunion Island." *American Journal of Tropical Medicine and Hygiene* 77 (4): 727–731. doi:77/4/727

Rezza G, Nicoletti L, Angelini R, Romi R, Finarelli AC, Panning M, Cordioli P, Fortuna C, Boros S, Magurano F, Silvi G, Angelini P, Dottori M, Ciufolini MG, Majori GC, Cassone A; CHIKV study group. 2007. "Infection with Chikungunya Virus in Italy: An Outbreak in a Temperate Region."

Lancet 370 (9602): 1840–1846. doi:10.1016/S0140-6736(07)61779-6.

Robillard PY, Boumahni B, Gérardin P, Michault A, Fourmaintraux A, Schuffenecker I, Carbonnier M, Djémili S, Choker G, Roge-Wolter M, Barau G. 2006. “Transmission Verticale Materno-Fœtale Du Virus Chikungunya. [Vertical maternal fetal transmission of the chikungunya virus. Ten cases among 84 pregnant women].” *La Presse Médicale* 35 (5): 785–788. doi:10.1016/S0755-4982(06)74690-5.

Ross, R W. 1956. “The Newala Epidemic III. the Virus: Isolation, Pathogenic Properties and Relationship To the Epidemic.” *Journal of Hygiene (London)* 177–191. doi:10.1017/S0022172400044442.

Selvam S, Thomas T, Shetty P, Zhu J, Raman V, Khanna D, Mehra R, Kurpad AV, Srinivasan K. 2016. “Norms for Developmental Milestones Using VABS-II and Association with Anthropometric Measures among Apparently Healthy Urban Indian Preschool Children.” *Psychological Assessment* 28 (12): 1634–1645. doi:10.1037/pas0000295.

Schilte C, Couderc T, Chrétien F, Sourrisseau M, Gagneux N, Guivel-Benhassine F, Kraxner A, Tschopp J, Higgs S, Michault A, Arenzana-Seisdedos F, Colonna M, Peduto L, Schwartz O, Lecuit M, Albert ML. 2010. *"Type 1 IFN controls chikungunya virus via its action on non hematopoietic cells." Journal of Experimental Medicine* 207(2):429-442. doi: 10.1084/jem.20090851.

Serenius F, Ewald U, Farooqi A, Fellman V, Hafström M, Hellgren K, Maršál K, Ohlin A, Olhager E, Stjernqvist K, Strömberg B, Ådén U, Källén K; Extremely Preterm Infants in Sweden Study Group. 2016. “Neurodevelopmental Outcomes among Extremely Preterm Infants 6.5 Years after Active Perinatal Care in Sweden.” *JAMA Pediatrics* 170 (10): 954–963. doi:10.1001/jamapediatrics.2016.1210.

Serreau R, Maillard T, Verdier R, Bouchara L, Catteau C, Hervé C, Fourmaintraux A, Lamblin D, Lesure JF, Jacqz-Aigrain E. 2002. “Étude Clinique et Prévalence Du Syndrome d’alcoolisation Fœtale Pris En Charge Dans Les Établissements Médicosociaux de l’île de La Réunion. [Clinical study and prevalence of fetal alcohol syndrome in medico-social institutions of the Reunion Island].” *Archives de Pediatrie* 9 (1): 14–20. doi:10.1016/S0929-693X(01)00688-1.

Sparrow SS, Balla SA, Cicchetti DV, Doll EA. 2005. “Vineland-II: Vineland adpative behavior scales: survey forms manual.” Ed: Circle Pines, Minnesota. AGS Publishing. 330 pp.

Tandale BV, Sathe PS, Arankalle VA, Wadia RS, Kulkarni R, Shah SV, Shah SK, Sheth JK, Sudeep AB, Tripathy AS, Mishra AC. 2009. “Systemic Involvements and Fatalities during Chikungunya Epidemic in India, 2006.” *Journal of Clinical Virology* 46(2): 145–149. doi:10.1016/j.jcv.2009.06.027.

Touret Y, Randrianaivo H, Michault A, Schuffenecker I, Kauffmann E, Lenglet Y, Barau G, Fourmaintraux A. 2006. “Transmission Materno-Fœtale Précoce Du Virus Chikungunya. [Early maternal-fetal transmission of the Chikungunya virus]. ” *La Presse Médicale* 35 (11): 1656–1658. doi:10.1016/S0755-4982(06)74874-6.

Trentini F, Poletti P, Baldacchino F, Drago A, Montarsi F, Capelli G, Rizzoli A. 2018. “The Containment of Potential Outbreaks Triggered by Imported Chikungunya Cases in Italy : A Cost Utility Epidemiological Assessment of Vector Control Measures,” no. January: 1–9. doi:10.1038/s41598-018-274.43-9.

ANNEXES

ANNEXE 1A

Simulation n°1. Distribution normale, différences des moyennes supérieure à 0 (hypothèse unilatérale)

PASS 15.0.3 09/09/2018 14:44:37 1

Tests for Paired Means

Numeric Results for Wilcoxon Test (Normal Distribution)
Null Hypothesis: Mean of Paired Differences = 0, Alternative Hypothesis: Mean of Paired Differences > 0
Known standard deviation (Normal distribution z-test).

Power	N	Alpha	Beta	Mean of Paired Differences	S	Effect Size
0,93542	42	0,05000	0,06458	5,0	10,0	0,500
0,47460	42	0,05000	0,52540	5,0	20,0	0,250
0,27734	42	0,05000	0,72266	5,0	30,0	0,167
1,00000	42	0,05000	0,00000	10,0	10,0	1,000
0,93542	42	0,05000	0,06458	10,0	20,0	0,500
0,67844	42	0,05000	0,32156	10,0	30,0	0,333
1,00000	42	0,05000	0,00000	15,0	10,0	1,500
0,99903	42	0,05000	0,00097	15,0	20,0	0,750
0,93542	42	0,05000	0,06458	15,0	30,0	0,500

References
Machin, D., Campbell, M., Fayers, P., and Pinol, A. 1997. Sample Size Tables for Clinical Studies, 2nd Edition. Blackwell Science. Malden, MA.
Zar, Jerrold H. 1984. Biostatistical Analysis (Second Edition). Prentice-Hall. Englewood Cliffs, New Jersey.
Al-Sunduqchi, Mahdi S. 1990. Determining the Appropriate Sample Size for Inferences Based on the Wilcoxon Statistics. Ph.D. dissertation under the direction of William C. Guenther, Dept. of Statistics, University of Wyoming, Laramie, Wyoming.

Report Definitions
Power is the probability of rejecting a false null hypothesis.
N is the number of pairs drawn from the population.
Alpha is the probability of rejecting a true null hypothesis.
Beta is the probability of accepting a false null hypothesis.
Mean of Paired Differences is the mean difference at which the power and sample size are determined.
S is the standard deviation of the paired differences for the population. It is assumed to be known, and the normal distribution z-test is used in place of the t-test.
Effect Size, |Mean of Paired Differences|/Sigma, is the relative magnitude of the effect under the alternative.

Summary Statements
A sample size of 42 achieves 94% power to detect a mean of paired differences of 5,0 with a known standard deviation of differences of 10,0 and with a significance level (alpha) of 0,05000 using a one-sided Wilcoxon test assuming that the actual distribution is normal.

Dropout-Inflated Sample Size

Dropout Rate	Sample Size N	Dropout-Inflated Enrollment Sample Size N'	Expected Number of Dropouts D
20%	42	53	11

Tests for Paired Means

Definitions

Dropout Rate (DR) is the percentage of subjects (or items) that are expected to be lost at random during the course of the study and for whom no response data will be collected (i.e. will be treated as "missing").
N is the evaluable sample size at which power is computed (as entered by the user). If N subjects are evaluated out of the N' subjects that are enrolled in the study, the design will achieve the stated power.
N' is the total number of subjects that should be enrolled in the study in order to end up with N evaluable subjects, based on the assumed dropout rate. N' is calculated by inflating N using the formula N' = N / (1 - DR), with N' always rounded up. (See Julious, S.A. (2010) pages 52-53, or Chow, S.C., Shao, J., and Wang,
H. (2008) pages 39-40.)
D is the expected number of dropouts. D = N' - N.

Chart Section

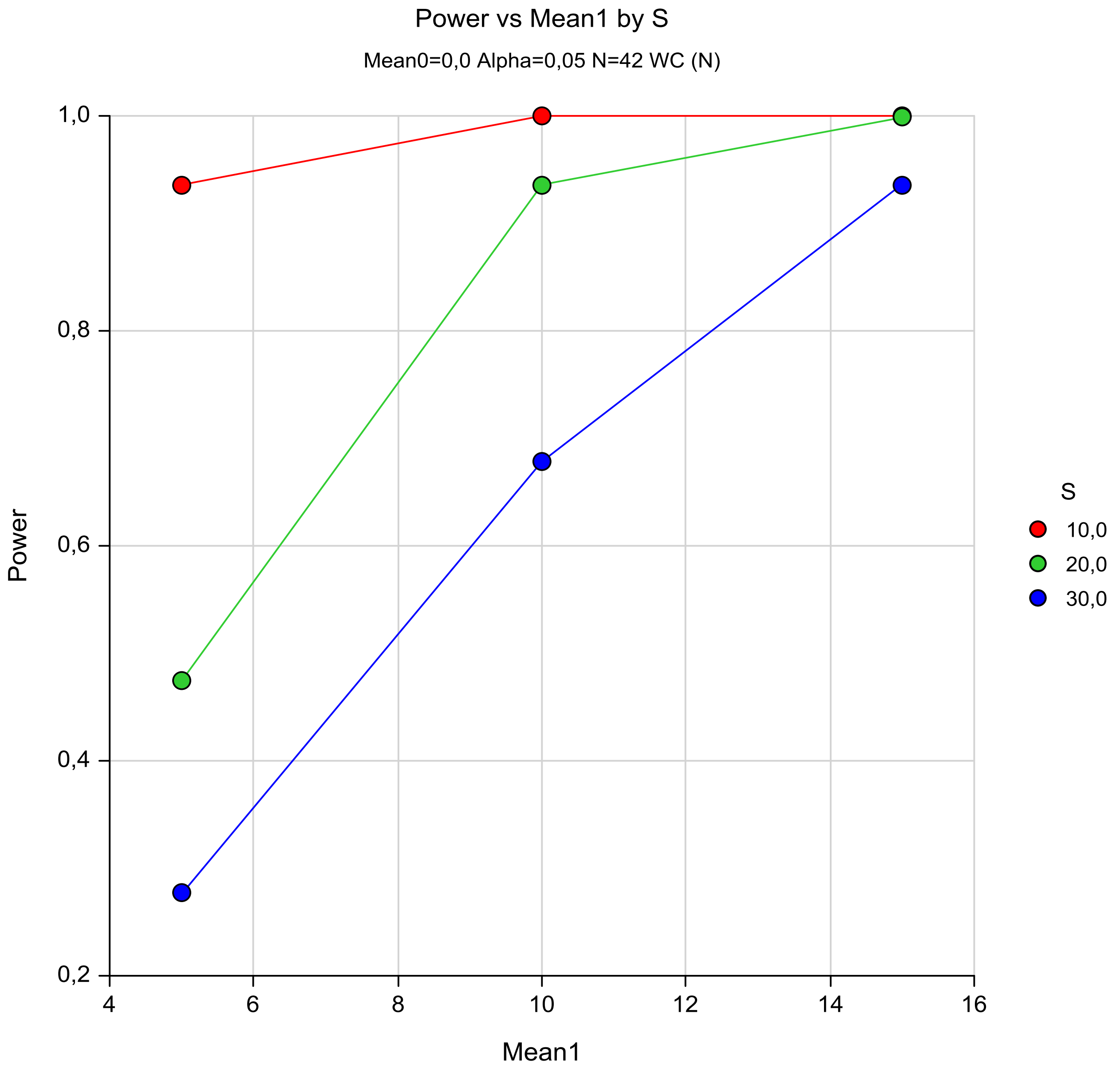

Tests for Paired Means

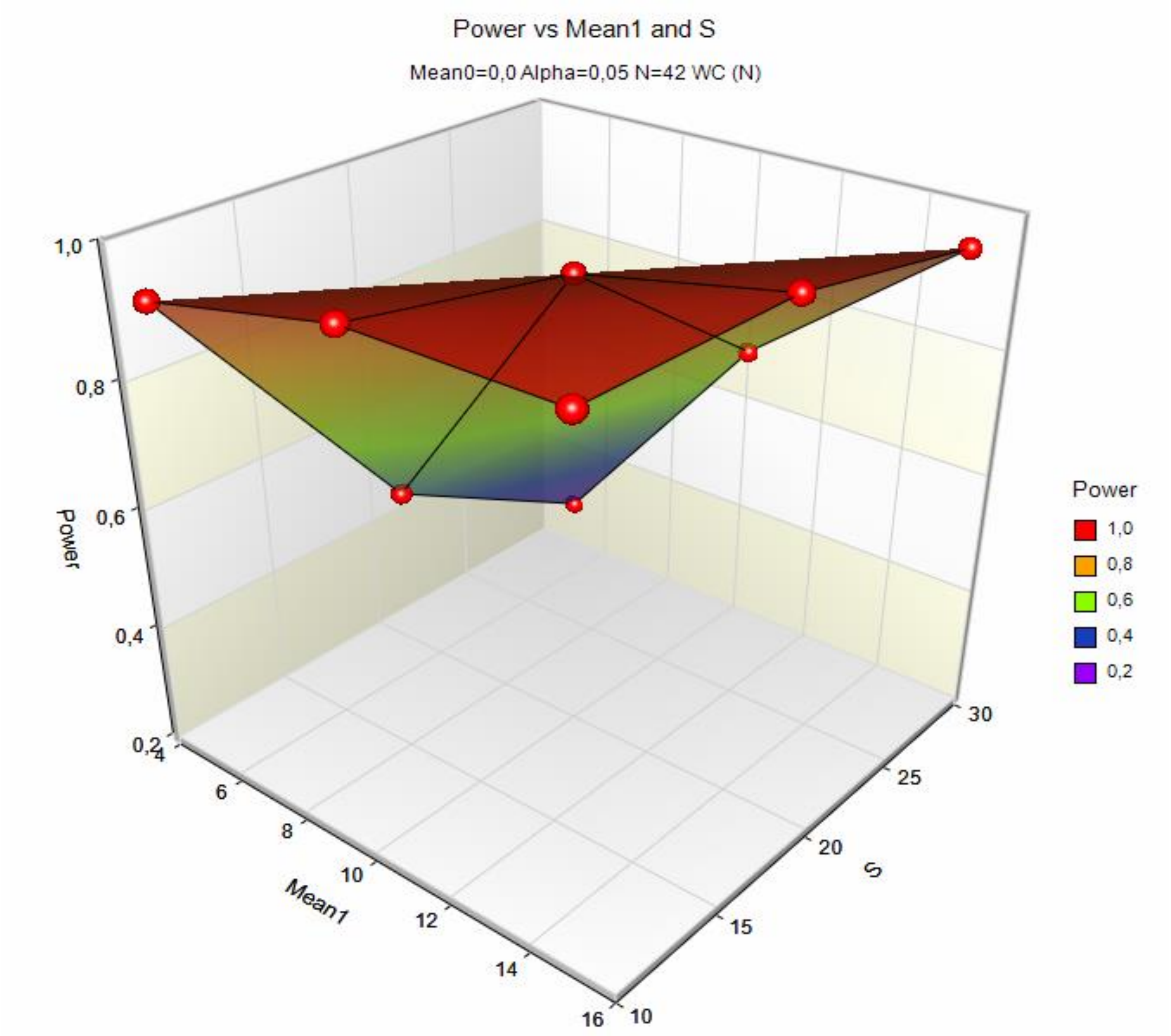

Tests for Paired Means

Procedure Input Settings

Autosaved Template File
G:\Users\patrick.gerardin\Documents\PASS 15\Procedure Templates\Autosave\Tests for Paired Means - Autosaved 2018_9_9-14_44_39.t155

Design Tab

Solve For:	Power
Alternative Hypothesis:	Ha: Mean of Paired Diffs > 0
Nonparam. Adj. (Wilcoxon Test):	Normal
Population Size:	Infinite
Alpha:	0,05
N (Sample Size):	42
Mean of Paired Differences (Alternative):	5 10 15
S (SD of Paired Differences):	10 20 30
Known Standard Deviation	Checked

ANNEXE 1B

Simulation n°2. Distribution normale, différences des moyennes non nulles sans préjuger du sens (hypothèse bilatérale)

PASS 15.0.3 09/09/2018 14:39:49 1

Tests for Paired Means

Numeric Results for Wilcoxon Test (Normal Distribution)
Null Hypothesis: Mean of Paired Differences = 0, Alternative Hypothesis: Mean of Paired Differences ≠ 0
Known standard deviation (Normal distribution z-test).

Power	N	Alpha	Beta	Mean of Paired Differences	S	Effect Size
0,88538	42	0,05000	0,11462	5,0	10,0	0,500
0,35261	42	0,05000	0,64739	5,0	20,0	0,250
0,18379	42	0,05000	0,81621	5,0	30,0	0,167
0,99999	42	0,05000	0,00001	10,0	10,0	1,000
0,88538	42	0,05000	0,11462	10,0	20,0	0,500
0,55894	42	0,05000	0,44106	10,0	30,0	0,333
1,00000	42	0,05000	0,00000	15,0	10,0	1,500
0,99731	42	0,05000	0,00269	15,0	20,0	0,750
0,88538	42	0,05000	0,11462	15,0	30,0	0,500

References
Machin, D., Campbell, M., Fayers, P., and Pinol, A. 1997. Sample Size Tables for Clinical Studies, 2nd Edition. Blackwell Science. Malden, MA.
Zar, Jerrold H. 1984. Biostatistical Analysis (Second Edition). Prentice-Hall. Englewood Cliffs, New Jersey.
Al-Sunduqchi, Mahdi S. 1990. Determining the Appropriate Sample Size for Inferences Based on the Wilcoxon
Statistics. Ph.D. dissertation under the direction of William C. Guenther, Dept. of Statistics, University of Wyoming, Laramie, Wyoming.

Report Definitions
Power is the probability of rejecting a false null hypothesis.
N is the number of pairs drawn from the population.
Alpha is the probability of rejecting a true null hypothesis.
Beta is the probability of accepting a false null hypothesis.
Mean of Paired Differences is the mean difference at which the power and sample size are determined.
S is the standard deviation of the paired differences for the population. It is assumed to be known, and the normal distribution z-test is used in place of the t-test.
Effect Size, |Mean of Paired Differences|/Sigma, is the relative magnitude of the effect under the alternative.

Summary Statements
A sample size of 42 achieves 89% power to detect a mean of paired differences of 5,0 with a known standard deviation of differences of 10,0 and with a significance level (alpha) of 0,05000 using a two-sided Wilcoxon test assuming that the actual distribution is normal.

Dropout-Inflated Sample Size

Dropout Rate	Sample Size N	Dropout-Inflated Enrollment Sample Size N'	Expected Number of Dropouts D
20%	42	53	11

Tests for Paired Means

Definitions

Dropout Rate (DR) is the percentage of subjects (or items) that are expected to be lost at random during the course of the study and for whom no response data will be collected (i.e. will be treated as "missing").
N is the evaluable sample size at which power is computed (as entered by the user). If N subjects are evaluated out of the N' subjects that are enrolled in the study, the design will achieve the stated power.
N' is the total number of subjects that should be enrolled in the study in order to end up with N evaluable subjects, based on the assumed dropout rate. N' is calculated by inflating N using the formula N' = N / (1 - DR), with N' always rounded up. (See Julious, S.A. (2010) pages 52-53, or Chow, S.C., Shao, J., and Wang,
H. (2008) pages 39-40.)
D is the expected number of dropouts. D = N' - N.

Chart Section

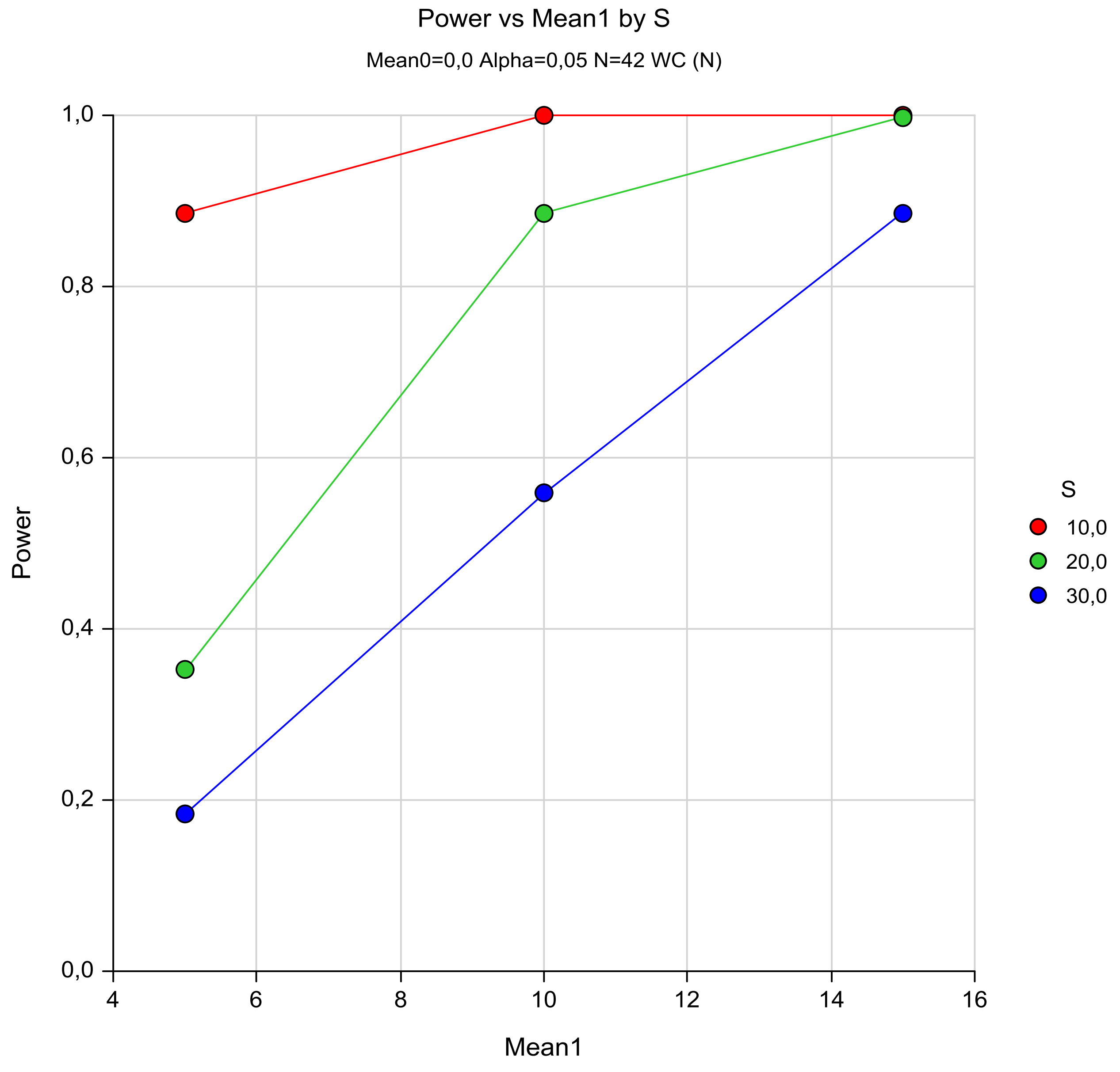

Tests for Paired Means

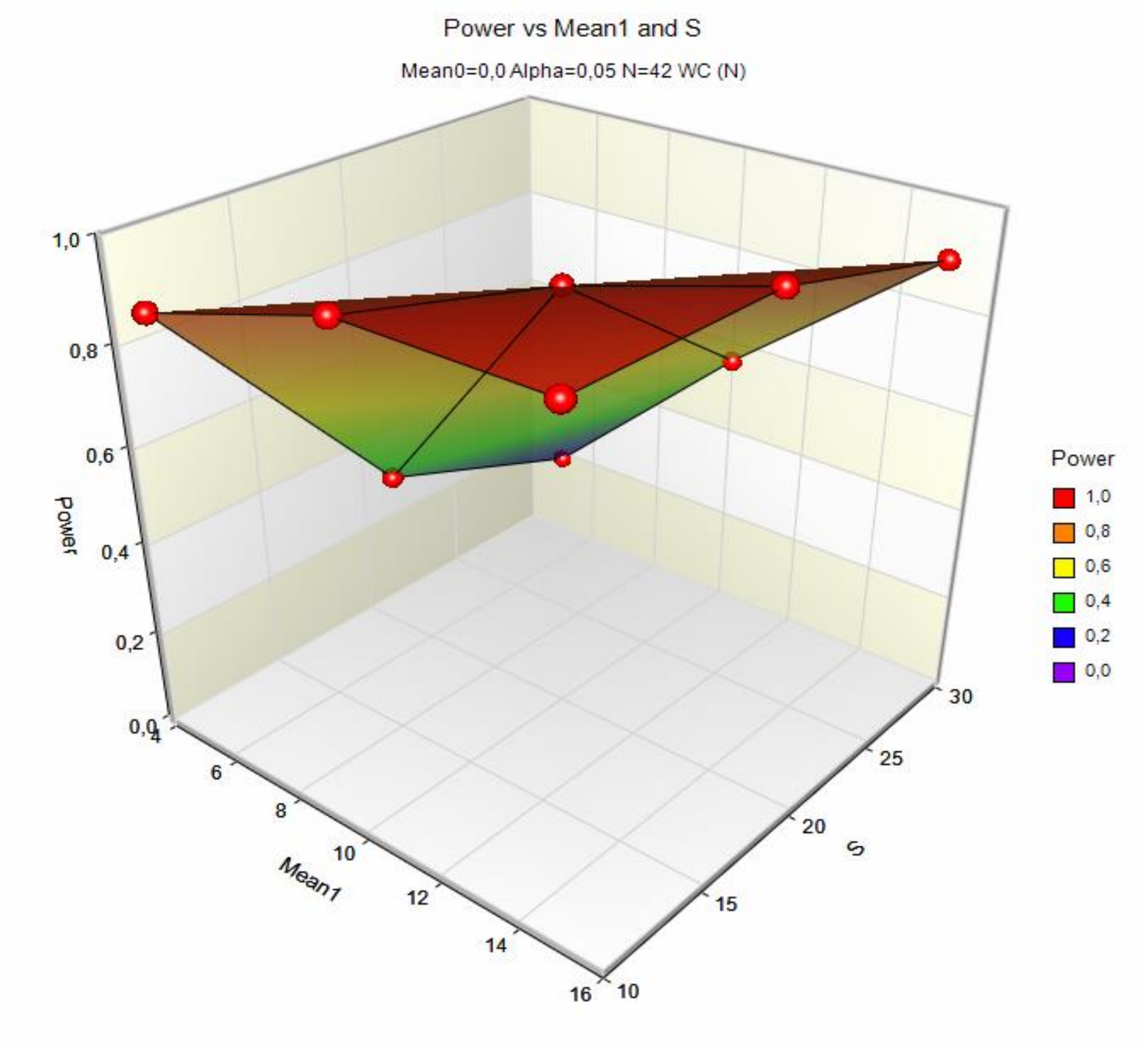

Tests for Paired Means

Procedure Input Settings

Autosaved Template File
G:\Users\patrick.gerardin\Documents\PASS 15\Procedure Templates\Autosave\Tests for Paired Means - Autosaved 2018_9_9-14_39_51.t155

Design Tab

Solve For:	Power
Alternative Hypothesis:	Ha: Mean of Paired Diffs ≠ 0
Nonparam. Adj. (Wilcoxon Test):	Normal
Population Size:	Infinite
Alpha:	0,05
N (Sample Size):	42
Mean of Paired Differences (Alternative):	5 10 15
S (SD of Paired Differences):	10 20 30

Known Standard Deviation Checked
